# Selective prebiotic conversion of pyrimidine and purine anhydronucleosides into Watson-Crick base-pairing *arabino*-furanosyl nucleosides in water

**DOI:** 10.1038/s41467-018-06374-z

**Published:** 2018-10-04

**Authors:** Samuel J. Roberts, Rafał Szabla, Zoe R. Todd, Shaun Stairs, Dejan-Krešimir Bučar, Jiří Šponer, Dimitar D. Sasselov, Matthew W. Powner

**Affiliations:** 1 Department of Chemistry, University College London, 20 Gordon Street, London, WC1H 0AJ UK; 2 Institute of Physics, Polish Academy of Sciences, Al. Lotników 32/46, PL-02668 Warsaw, Poland; 3 Institute of Biophysics of the Czech Academy of Sciences, Královopolská 135, 61265 Brno, Czech Republic; 4 Harvard-Smithsonian Center for Astrophysics, Department of Astronomy, Harvard University, 60 Garden Street, Cambridge, MA 02138 USA

## Abstract

Prebiotic nucleotide synthesis is crucial to understanding the origins of life on Earth. There are numerous candidates for life’s first nucleic acid, however, currently no prebiotic method to selectively and concurrently synthesise the canonical Watson–Crick base-pairing pyrimidine (C, U) and purine (A, G) nucleosides exists for any genetic polymer. Here, we demonstrate the divergent prebiotic synthesis of arabinonucleic acid (ANA) nucleosides. The complete set of canonical nucleosides is delivered from one reaction sequence, with regiospecific glycosidation and complete furanosyl selectivity. We observe photochemical 8-mercaptopurine reduction is efficient for the canonical purines (A, G), but not the non-canonical purine inosine (I). Our results demonstrate that synthesis of ANA may have been facile under conditions that comply with plausible geochemical environments on early Earth and, given that ANA is capable of encoding RNA/DNA compatible information and evolving to yield catalytic ANA-zymes, ANA may have played a critical role during the origins of life.

## Abstract (web summary)

Arabinonucleic acid (ANA) Watson-Crick base-pair with RNA/DNA and can evolve to display enzyme-like function. Here, the authors now identify a prebiotic pathway that yields the complete set of Watson-Crick base-pairing purine and pyrimidine ANA nucleosides.

## Introduction

**Fig. 1:**
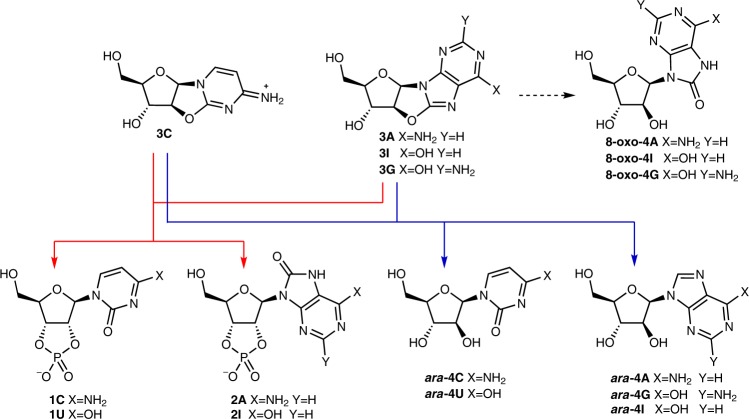
Divergent prebiotic nucleotides synthesis. Red arrows: Previous work; a prebiotic pathway to cytidine-2′,3′-cyclic phosphate (**1C**), uridine-2′,3′-cyclic phosphate (**1U**), 8-oxo-adenosine-2′,3′-cyclic phosphate (**2A**) and 8-oxo-inosine-2′,3′-cyclic phosphate (**2I**)^16,22^. Dashed arrow: Hydrolysis of 8,2′-anhydropurines (**3A**, **3I** and **3G**) is not observed, which provides chemical differentiation from 2,2′-anhydropyrimidine (**3C**) that readily hydrolyses to β-*arabino*-adenosine (***ara*-4C**). Blue arrows: This work; a prebiotic pathway to β-*arabino*-cytidine (***ara*-4C**), β-*arabino*-uridine (***ara*-4U**), β-*arabino*-adenosine (***ara*-4A**), β-*arabino*-inosine (***ara*-4I**) and β-*arabino*-guanosine (***ara*-4G**)

The synthesis of the complete set of canonical Watson–Crick base-pairing nucleosides [adenosine (A), cytidine (C), guanosine (G) and uridine (U)] under conditions that do not violate the accepted plausible geochemical environments on early Earth is an essential step towards elucidating the origins of life on Earth^1–3^. However, while many 'plausible' nucleoside candidates have been suggested to have played a role at the origins of life^4–6^, the concurrent prebiotic synthesis of a complete set of nucleoside monomers remains an unresolved challenge for any of the proposed genetic polymers [e.g., ribonucleic acid (RNA), arabinonucleic acid (ANA), threonucleic acid (TNA) and pyranosyl-ribonucleic acid (pRNA)]^3,5,7–24^. Accordingly, we set out to elucidate chemical reactions that could address this problem. We have previously reported a prebiotic synthesis of pyrimidine ribonucleotides **1C** and **1U**^16^. More recently, we reported the divergent synthesis of **1C**, **1U** and 8-oxo-purine ribonucleotides **2A** and **2I** (Fig. 1, red arrows)^22^. However, no divergent prebiotic synthesis of pyrimidine and purine nucleoside monomers bearing the canonical Watson–Crick base-pairing nucleobases has yet been elucidated^7,21–23^.

ANA displays many properties that make it an attractive candidate for the first genetic polymer of life. ANAs can equilibrate between helix and stem-loop structures, which mimic DNA and RNA, respectively^25^. ANA can form a complementary Watson–Crick base-paired duplex with RNA^26,27^, and can be readily transcribed (from DNA) and reverse transcribed (to DNA)^28^. Additionally, Holliger and co-workers recently evolved catalytic ANA-zymes that can achieve RNA phosphodiester cleavage^6^. Notably, the ANA phosphodiester backbone is also far more resistant to hydrolysis than its RNA analogue^26^.

Anhydrocytidine (**3C**), a key intermediate in our previously reported prebiotic pyrimidine synthesis^16^, undergoes hydrolysis at near neutral pH (≥6.5)^22^ to quantitatively afford *arabino*-cytidine (***ara*-4C**; Fig. 1, blue arrow). This facile hydrolysis suggests that a simple prebiotic synthesis of *arabino*-nucleotides may be achievable. Importantly, the synthesis of a complete set of arabinosides requires differential reactivity between the purine and pyrimidine precursors **3A**/**3G** and **3C** (Fig. 1), respectively^16,22^. Pyrimidine ***ara*-4C** can be accessed by direct hydrolysis of **3C**, whereas hydrolysis of **3A** and **3G** would furnish 8-oxo-purines (**8-oxo-4A** and **8-oxo-4G**; Fig. 1, dashed arrow)^22,29^ rather than the desired purine nucleosides ***ara*-4A** and ***ara*-4G**. Therefore, it is of note that purine precursors **3A**^22^, **3I** and **3G** are highly resistant to alkaline hydrolysis, even in extremely alkaline (pH >12) solutions. Accordingly, we viewed this subtle, yet pronounced, difference in reactivity as an ideal source for chemical differentiation that could be exploited while building the canonical nucleobases on a preformed furanosyl-sugar scaffold. We envisaged sulfur—a critical element in the development of divergent ribonucleoside syntheses^22^, with widespread use in prebiotic chemistry^22,30–35^—would hold the key to site-selective purine reduction.

Here, we demonstrate a divergent route to synthesise a complete set of canonical (A, G, C and U) nucleosides from one plausibly prebiotic reaction sequence. Interestingly, photochemical reduction of (intermediate) 8-mercaptopurines is observed to be highly efficient for the desired canonical purines (A and G), but not the non-canonical purine inosine (I). The facile prebiotic synthesis of ANA indicates that it may have played an important role during the origins of life, and the selective photochemical reduction of 8-mercaptopurines provides a physical mechanism for prebiotic nucleobase selection en route to the Watson–Crick base-pairing nucleosides.

## Results

### 8-Mercaptopurine synthesis

**Fig. 2:**
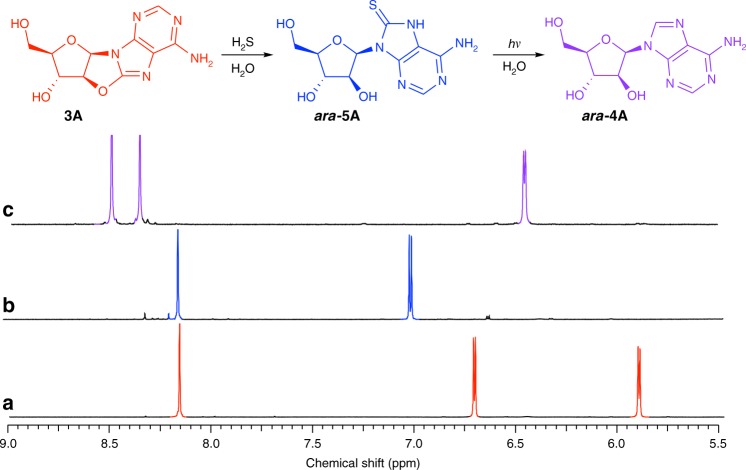
Prebiotic synthesis of *arabino*-adenosine (***ara*-4A**). ^1^H NMR spectra (600 MHz, 9:1 H_2_O/D_2_O, 25 °C, *δ* = 5.5–9.0 ppm) showing: **a** anhydroadenosine (**3A**; red). **b** Crude 8-mercapto-*arabino*-adenosine (***ara*-5A**; blue) observed upon incubation of anhydroadenosine (**3A**; 35.7 mM) and H_2_S (670 mM, 60 °C, 7 d, pH 7). **c** Crude *arabino*-adenosine (***ara*-4A**; purple) after irradiation (*λ* = 300 nm) of 8-mercapto-*arabino*-adenosine (***ara*-5A**; 2 mM, room temperature, 16 h, pH 7)

We suspected that addition of hydrogen sulfide (H_2_S) to **3A**, **3G** and **3I** in water would selectively introduce sulfur at the C8-carbon atom, and consequently direct regiospecific reduction of the canonical purine nucleobases on the preformed sugar scaffold (Fig. 1, blue arrows). Previously, Ikehara and Ogiso established that **3A** reacts with liquid H_2_S in pyridine at 100 °C (sealed in a steel tube) to afford 8-mercapto-*arabino*-adenosine (***ara***-**5A**)^36^. However, **3A**, **3G** and **3I** were remarkably stable to alkaline hydrolysis (Supplementary Figs. 1 and 2). Therefore, we began our investigation by exploring the mild, plausibly prebiotic, aqueous thiolysis of the prebiotic purine precursor **3A**^22,33,35^. Incubation of H_2_S (670 mM) with **3A** (35.7 mM) in water (pH 7, 60 °C, 7 d) afforded remarkably clean conversion of **3A** to ***ara***-**5A** (73%; Fig. 2, Table 1 and Supplementary Fig. 3).

**Table 1:** Percentage conversions for nucleoside reactions

Conversion	H_2_S	Conversion	254	300	H_2_O_2_
**3A**→***ara*-5A**	73%	***ara*-5A**→***ara*-4A**	70%	66%	85%
—	—	**β-*ribo*-5A**→**β-*ribo*-4A**	—	70%	—
**3G**→***ara*-5G**	83%	**3G**→→***ara*-4G**	—	59%^a^	89%
—	—	**β-*ribo*-5G**→**β-*ribo*-4G**	—	80%	—
**3I**→***ara*-5I**	78%	***ara*-5I**→***ara*-4I**	—	15%	90%
—	—	**β-*ribo*-5I**→**β-*ribo*-4I**	—	10%	—
**3C**→***ara*-4C**	91%^b^	—	—	—	—
**α-*ribo*-3C**→**α-*ribo*-4C**	52%^c^	—	—	—	—
**3C**→***ara*-6C**	31%^d^	***ara*-6C**→**3C**	—	—	Quant.
**α-*ribo*-3C**→**α-*ribo*-6C**	84%^d,35^	**α-*ribo*-6C**→**α-*ribo*-3C**	—	—	Quant.
—	—	**β-*ribo*-6C**→**β-*ribo*-4C**	—	—	Quant.
—	—	**β-*ribo*-6C**→**β-*ribo*-9**	—	—	76%^e^
***ara*-4C**→***ara*-7U**	40%^f^	***ara*-7U**→***ara*-4U**	8%	4%	78%
**α-*ribo*-4C**→**α-*ribo*-7U**	63%^f^	**α-*ribo*-7U**→**α-*ribo*-4U**	—	—	93%
**β-*ribo*-4C**→**β-*ribo*-7U**	16%^f^	**β-*ribo*-7U**→**β-*ribo*-4U**	—	—	78%

Conversions were directly determined by ^1^H NMR (600 MHz) spectroscopy in the crude product mixture. Conversion observed upon: H_2_S: reaction with H_2_S (20 equiv.) in water (pH 7, 60 °C, 7 d); 254: irradiation at *λ* = 254 nm in water (38 °C, pH 6.5, 16 h); 300: irradiation at *λ* = 300 nm in water (38 °C, pH 6.5, 16 h); H_2_O_2_: reaction with H_2_O_2_ (3 equiv.) in water (pH 7, room temperature)

^a^22 h irradiation

^b^Conversion at room temperature. Conversion of ***ara*-4C** to ***ara*-7U** occurs at 60 °C over 7 d to afford a mixture of ***ara*-4C**/***ara*-7U** (1.5:1) in 95% combined conversion

^c^Unoptimised hydrolysis observed after 7 d incubation with H_2_S (20 equiv.) in water (60 °C, pH 7), observed alongside 39% hydrolysis of **α-*ribo*-4C** to **α-*ribo*-7U** (91% combined **α-*ribo*-4C** + **α-*ribo*-7U**)

^d^Conversion in formamide with H_2_S (4 equiv.)

^e^Buffered at pH 3 with glycine; **β-*ribo*-9** observed alongside partial hydrolysis to **β-*ribo*-4C** (24**%**)

^f^Unoptimised thiolysis observed after 7 d incubation with H_2_S (20 equiv.) at 60 °C and pH 7 to investigate comparative rates of cytidine C4-thiolysis. Residual starting material ***ara*-4C** (52%), **α-*ribo*-4C** (37%) and **β-*ribo*-4C** (84%), respectively, accounted (>90%) for the residual mass balance

Intrigued by the remarkably clean, high-conversion thiolysis of **3A**, we next investigated the thiolysis (pH 7, 60 °C, 7 d) of **3G** and **3I**. We observed ***ara***-**5G** (83%) and ***ara***-**5I** (78%; 71% isolated yield after 5 d) (Table 1 and Supplementary Figs. 4 and 5). Next, 1:1 **3A**/**3I** was subjected to thiolysis and gave a high conversion to both ***ara***-**5A** (66%) and ***ara***-**5I** (75%) (Supplementary Fig. 6). Even under these mild, aqueous conditions, highly efficient thiolysis of **3A**, **3G** and **3I** was observed, demonstrating that sulfur can be regioselectively introduced to the C8-carbon atom of purines under plausibly prebiotic conditions.

### Photochemical purine reduction

For purine reduction, we initially investigated the effect of UV light on 8-mercaptopurines **5**, because the atmosphere of the early Earth lacked an ozone layer^37^, allowing UV light (*λ* > 204 nm) to irradiate Earth’s surface^38,39^. We envisaged that UV irradiation of ***ara***-**5A** would lead to π–π* excitation, followed by C–S bond fragmentation to afford an N-heterocyclic carbene tautomer of ***ara***-**4A**. Pleasingly, when ***ara***-**5A** (2.00 mM, pH 7) was irradiated (*λ* = 300 or 254 nm) in water we observed extremely clean conversion to ***ara***-**4A** in 66% and 70%, respectively, after 16 h (Fig. 2 and Supplementary Figs. 7–9). Notably, ***ara***-**4A** was the only nucleoside product observed after irradiation. Irradiation (*λ* = 300 nm) of the inosine analogue ***ara***-**5I** gave *arabino*-inosine ***ara***-**4I** (Supplementary Fig. 10). However, upon complete consumption of ***ara***-**5I**, the observed conversion to ***ara***-**4I** (15%) was significantly lower than for ***ara***-**4A** (66%) (Table 1). Next, the two-step thiolysis/irradiation sequence was investigated for **3G**. Anhydronucleoside **3G** (5.85 mM) was thiolysed (H_2_S, pH 7, 60 °C, 7 d) and then irradiated (*λ* = 300 nm). The reaction was monitored until complete consumption of the intermediate (***ara***-**5G**) was observed (Supplementary Fig. 11). Once again, we observed a good conversion (59% after 22 h; Table 1) to the desired product *arabino*-guanosine (***ara***-**4G**). Irradiation of 1:1 ***ara***-**5A**/***ara***-**5I** confirmed the disparity between the conversions observed for the reduction to yield the canonical nucleobases A and G and wobble base-pairing I: 62% and 13% conversion to ***ara***-**4A** and ***ara***-**4I**, respectively, was observed (Supplementary Fig. 12). Notably, the irradiation (*λ* = 300 or 254 nm) of 1:1 ***ara***-**4A**/***ara***-**4I** (Supplementary Fig. 13) demonstrated equal product stability with respect to UV irradiation (e.g., 73% each after 16 h at *λ* = 300 nm), and therefore did not account for the intriguing differential percentage conversion observed between the canonical (A and G) and non-canonical (I) 8-mercapto-nucleosides. Subsequently, we irradiated the *ribo*-mercaptopurines ***ribo***-**5A**, ***ribo***-**5G** and ***ribo***-**5I**, and observed highly efficient photo-reduction of the A and G *ribo*-nucleosides, but poor yielding reduction of the I *ribo*-nucleoside (Table 1 and Supplementary Figs. 14–16).

### Quantum chemical studies

The observed difference in the photo-reduction of **5A** and **5G** compared to **5I** cannot be directly connected to any remarkable differences in their UV-absorption features (Supplementary Figs. 117–119). However, quantum chemical studies [ADC(2)]^40,41^ and femtosecond transient absorption spectroscopy (FTAS) suggest that after initial UV excitation these purines undergo different singlet-to-triplet decay pathways.

**Fig. 3:**
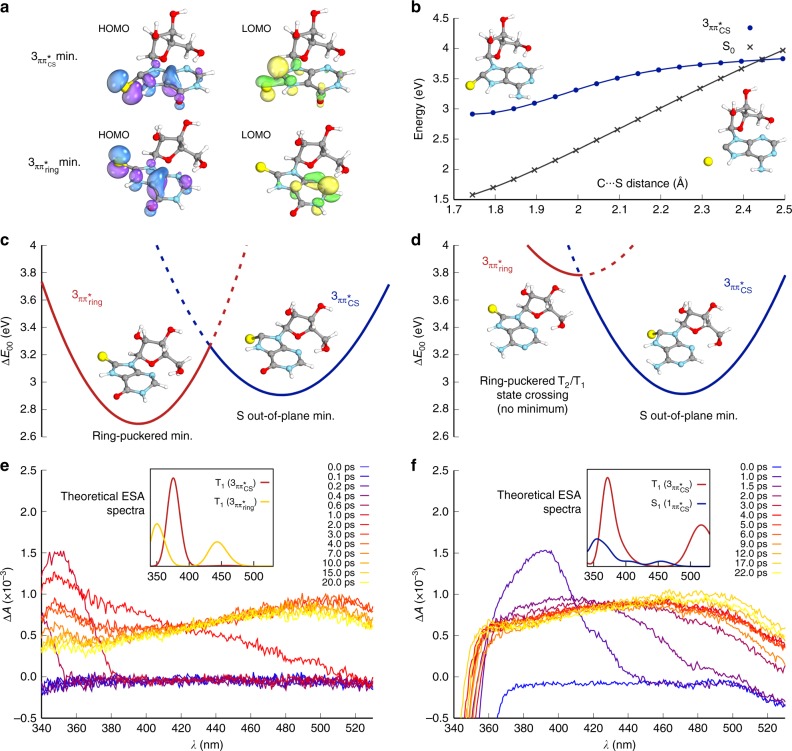
Photochemical properties of ***ara*-5I** and ***ara*-5A**. **a** Molecular orbitals for T_1_ states (^3^ππ_CS_* and ^3^ππ_ring_*) of ***ara*-5I**. **b** Calculated potential energy profile of ***ara*-5A** T_1_^3^ππ_CS_* state C–S bond homolysis furnishing triplet sulfur atom and singlet C8-carbene tautomer of ***ara*-4A**. Blue line = T_1_-state energy, black line = ground-state energy. The minimum-energy path along the C–S distance (Å) was obtained by calculation at the ADC(2)/cc-pVTZ level of theory. **c** Calculated ***ara*-5I** triplet excited state T_1_ topography. Parabolas fitted to calculated T_1_ minima energies and optimised T_2_/T_1_ minimum-energy state crossing, which represents the transition state between these minima. **d** Calculated ***ara*-5A** triplet excited state T_1_ topography. No ring-puckered minimum was found on the T_1_ hypersurface due to the higher ^3^ππ_ring_* state energy. **e**–**f** FTAS recorded between *λ* = 340 and 530 nm with *λ* = 290 nm excitation (pump) pulses. **e** FTAS of ***ara*-5I** recorded between *λ* = 340 and 530 nm with *λ* = 290 nm excitation (pump) pulses. Inset: Calculated excited state absorption spectra for the two possible configurations of ***ara*-5I**. **f** FTAS of ***ara*-5A** recorded between *λ* = 340 and 530 nm with *λ* = 290 nm excitation (pump) pulses. Inset: Calculated excited state absorption spectra for the singlet and triplet states of ***ara***-**5A**

UV excitation of mercaptopurines (**5**) was calculated to result in the population of low-lying excited singlet states (ππ_CS_*, ππ_RING_* and nπ_CS_*), which all exhibit significant spin–orbit coupling (SOC) with the triplet manifold (Supplementary Table 2 and Supplementary Discussion). Therefore, these singlet states are expected to undergo efficient intersystem crossing (ISC) to populate the triplet excited states of **5**, as noted for other thionucleosides and thionucleobases^42–45^. Two important triplet minima were identified in our calculations (Fig. 3). First, the T_1_(^3^ππ_CS_*) triplet minimum (Fig. 3a), which leads to C8-thiocarbonyl elongation (≈0.1 Å) and localisation of unpaired electrons on the *sp*^3^-hybridised C8-carbon and sulfur atoms. Second, the T_1_(^3^ππ_RING_*) triplet minimum (Fig. 3a), which leads to pyrimidine ring puckering and localisation of unpaired electrons on the partially *sp*^3^-hybridised C2- and C5-carbon atoms. The ***ara***-**5A** ^3^ππ_ring_* triplet is significantly higher in energy than the ***ara***-**5A** ^3^ππ_CS_* triplet; consequently, population of the ^3^ππ_CS_* triplet is predicted to be the dominant pathway during ISC following UV excitation of ***ara***-**5A** (Fig. 3d). Accordingly, we expect that C8-photo-reduction is triggered in the ^3^ππ_CS_* triplet state of 8-mercaptopurines **5**. In contrast to ***ara***-**5A**, which is expected to only populate the ^3^ππ_CS_* triplet state, ***ara***-**5I** and ***ara***-**5G** are predicted to populate both triplet states (^3^ππ_CS_* and ^3^ππ_ring_*). Optimisation of the mercaptopurine excited state geometries indicated that the T_1_ hypersurface of ***ara***-**5I** (Fig. 3c) and ***ara***-**5G** (Supplementary Fig. 51) are similar in topography to those reported for thiopyrimidine nucleobases^46^. Consequently, we expected ***ara***-**5I** and ***ara***-**5G** to exhibit similar UV excitation behaviour to thiopyrimidines, which are observed to undergo two competing excited state decay pathways^46^.

Short excited state lifetimes are expected for the redox active T_1_(^3^ππ_CS_*) triplet states, due to their very large SOC values (***ara***-**5A**: 99.5 cm^−1^; ***ara***-**5I**: 81.4 cm^−1^) and low (0.23 eV) state-crossing barrier to the electronic ground state (S_0_). Conversely, the ***ara***-**5I** T_1_(^3^ππ_RING_*) triplet state exhibits a very low SOC value (1.44 cm^−1^) and is predicted to be much longer lived. Moreover, the ***ara***-**5I** T_1_(^3^ππ_RING_*) triplet minimum is estimated to be 0.2 eV lower in energy than the redox active ***ara***-**5I** T_1_(^3^ππ_CS_*) triplet minimum, and predominant population of the non-redox active T_1_(^3^ππ_RING_*) triplet minimum is expected following UV excitation of ***ara***-**5I** because these two states are only separated by a moderate (0.55 eV) energy barrier.

Upon first inspection it might seem surprising that, like ***ara***-**5I**, both triplet states can be populated in ***ara***-**5G** given its observed efficient photo-reduction. However, the ***ara***-**5G** T_1_(^3^ππ_RING_*) and T_1_(^3^ππ_CS_*) triplet minima are calculated to be nearly isoenergetic, and therefore these minima are expected to easily interconvert. Accordingly, efficient photo-reduction of ***ara***-**5G** is thought to occur by continual repopulation of the redox active ***ara***-**5G** T_1_(^3^ππ_CS_*) triplet minimum.

### Femtosecond transient absorption spectroscopy

We next sought to verify our theoretical predictions by FTAS for ***ara***-**5I** (Fig. 3e) and ***ara***-**5A** (Fig. 3f). The absorbance (*λ* = 380–450 nm) observed in the first picosecond following ***ara***-**5A** excitation matches the position and structure of the excited state absorption (ESA) spectrum simulated from the S_1_(^1^ππ_CS_*) singlet minimum of ***ara***-**5A** (Fig. 3f, inset). Initial singlet (^1^ππ_CS_*) state population following photo-excitation is also consistent with our calculated vertical excitation energies (Supplementary Table 1), which indicate that *λ* = 290–300 nm excitation would primarily populate this state (Fig. 3e). At probe time delays >2 ps after incident excitation of ***ara***-**5A**, the absorbance evolved to cover a broader range of probe wavelengths, and the FTAS spectrum exhibits two characteristic features near *λ* = 360 and 480 nm. Although these features are partly covered by the bleaching bands, they match the simulated ***ara***-**5A** T_1_(^3^ππ_CS_*) triplet state ESA spectrum (Fig. 3f, inset), which indicates population of this triplet state. The observed excited state lifetime of ***ara***-**5A** (≈70 ns at *λ* = 510 nm) is also consistent with the predicted efficient photo-relaxation of this triplet state, which is characterised by high SOC with the electronic ground state.

Although excitation at *λ* = 290 nm could potentially populate both ***ara***-**5I** singlet states (i.e., ^1^ππ_CS_* and ^1^ππ_ring_*), we did not observe any features in the FTAS spectrum that would correspond to the ^1^ππ_CS_* singlet state. No significant change in absorbance was recorded during the first 0.4 ps (Fig. 3e). We were unable to simulate the ESA spectrum for the ***ara***-**5I** S_1_(^1^ππ_ring_*) singlet state (its minimum-energy geometry coincided with the S_1_/S_0_ conical intersection), but we anticipate that population of this singlet state dominates the 0.4 ps immediately after ***ara***-**5I** excitation. The emergence of a sharp band at *λ* = 350 nm between 0.6 and 1.0 ps was assigned to population of the redox active T_1_(^3^ππ_CS_*) triplet state, which could be efficiently accessed from the initial S_1_(^1^ππ_ring_*) singlet state because of the molecular orbital change associated with an S_1_ → T_1_ transition^47^. The redox active ***ara***-**5I** T_1_(^3^ππ_CS_*) triplet minimum is observed to be efficiently depopulated over the next 6 ps. The excited state population transfers to the lower-energy ***ara***-**5I** T_1_(^3^ππ_ring_*) triplet minimum (Fig. 3e), which is confirmed by the long excited state lifetime (≈12 μs at *λ* = 440 nm) and the two characteristic bands (*λ*_max_ = 350 and 490 nm) in the ***ara***-**5I** FTAS measurements, which are both consistent with the simulated ESA spectrum for the redox inactive triplet state (Fig. 3e, inset). The estimated excited state lifetime associated with the T_1_(^3^ππ_ring_*) triplet state is thought to be sufficient to enable bimolecular reactions and, consequently, the photochemical degradation pathways in ***ara***-**5I**, which are not observed for ***ara***-**5A** or ***ara***-**5G**. Photo-reduction appears to correlate directly with population of the T_1_(^3^ππ_CS_*) triplet state for ***ara***-**5A**, and we anticipate that the same triplet state would be responsible for photo-reduction in ***ara***-**5I** and ***ara***-**5G**. In particular, the observed short-lived population of the ***ara***-**5I** T_1_(^3^ππ_CS_*) triplet state (0.6–7.0 ps) likely explains the poor photo-reduction observed for this nucleoside.

It appears probable that C–S bond homolysis in ***ara***-**5A**, initiated by population of the T_1_(^3^ππ_CS_*) triplet minimum, would furnish the singlet C8-carbene tautomer of purine ***ara***-**4A** (Fig. 3b). Our calculations indicate that C–S bond homolysis requires 0.9 eV to liberate the triplet sulfur atom. Excitation at *λ* = 300 nm would excite ***ara***-**5A** ~1.2 eV above the T_1_(^3^ππ_CS_*) triplet minimum, which would be sufficient to promote efficient photo-reduction. In principle, C–S bond homolysis (Fig. 3b) would be reversible, however tautomerisation of the intermediate C8-carbene to ***ara***-**4** would prevent reformation of ***ara***-**5**.

### 8-Mercaptopurine oxidation

Encouraged by the photochemical reduction of ***ara***-**5A**, ***ara***-**5G** and ***ara***-**5I**, we investigated how to increase the efficiency of purine reduction. Simplicity and prebiotic plausibility led us to consider hydrogen peroxide (H_2_O_2_) in nucleobase reduction^48–50^. In our hands, the reported reaction of H_2_O_2_ and ***ara***-**5A** in acidic methanol^36^ gave ***ara***-**4A** (50% after 16 h, Supplementary Fig. 17). Pleasingly, oxidative disproportionation of ***ara***-**5A** (50 mM) and ***ara***-**5I** (50 mM) under plausibly prebiotic conditions (H_2_O_2_ (3 equiv), pH 7, water, room temperature, 3 h) afforded excellent conversion to ***ara***-**4A** (85%) and ***ara***-**4I** (90%), respectively (Table 1 and Supplementary Figs. 18 and 19). Reaction of 1:1 ***ara***-**5A**/***ara***-**5I** afforded ***ara***-**4A** (88%) and ***ara***-**4I** (91%) (Supplementary Fig. 20). Next, we investigated the one-pot thiolysis/oxidation of **3G**. Incubation of **3G** (35 mM) with H_2_S (714 mM) in water (pH 7, 60 °C, 7 d) then addition of H_2_O_2_ (375 µmol; pH 7, room temperature, 2 h) furnished ***ara***-**4G** (89%) (Table 1 and Supplementary Fig.  21).

### Pyrimidine thiolysis

**Fig. 4:**
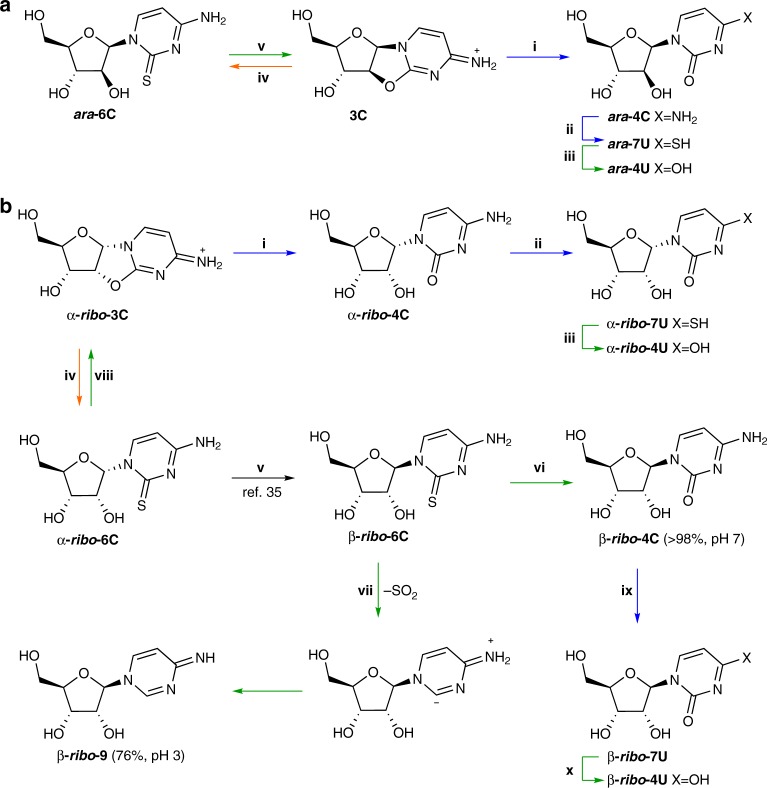
Thiolysis and oxidation of pyrimidine nucleosides. **a** i. H_2_S thiolysis of *arabino*-anhydrocytidine (**3C**) in water (pH 7) furnished β-*arabino*-cytidine (***ara*-4C**; quant.) rapidly; ii. ***ara*-4C** then undergoes slow addition of H_2_S to afford 4-thio-β-*arabino*-uridine (***ara*-7U**). iii. H_2_O_2_ oxidation of ***ara*-7U** in water (pH 7) furnished *arabino*-uridine (***ara*-4U**, 78%). iv. Thiolysis of **3C** in formamide yielded 2-thio-β-*arabino*-cytosine (***ara*-6C**, 31%). v. H_2_O_2_ oxidation of ***ara*-6C** in water (pH 7) furnished **3C** (quant.). **b** i–ii. H_2_S thiolysis of *ribo*-anhydrocytidine (α**-*ribo***-**3C**) in water (pH 7) furnished α-*ribo*-cytidine (α**-*ribo***-**4C**), which then undergoes addition of H_2_S to afford 4-thio-α-*ribo*-uridine (α**-*ribo***-**7U**) in 91% combined conversion. iii. H_2_O_2_ oxidation of α**-*ribo***-**7U** in water (pH 7) furnished α-*ribo*-uridine (α**-*ribo*-4U**, 93%). iv–v. Sutherland and co-workers reported the synthesis of 2-thio-β-*ribo*-cytidine (β-***ribo*-6C**) by thiolysis of *ribo*-anhydrocytidine (α**-*ribo***-**3C**) in formamide, followed by aqueous irradiation (*λ* = 254 nm)^35^. vi. H_2_O_2_ oxidation of 2-thio-β-*ribo*-cytosine (β**-*ribo*-6C**) furnished canonical ribonucleoside β-cytidine (β**-*ribo*-4C**, quant.) between pH 7 and 9. vii. Conversely, H_2_O_2_ oxidation of β**-*ribo*-6C** predominately furnished pyridimide 4-amino-pyrimidine-riboside (β**-*ribo*-9**, 76%) at pH 3. viii. Due to the proximity of the nucleobase and C2′-hydroxyl moieties, H_2_O_2_ oxidation of α**-*ribo*-6C** furnished α**-*ribo*-3C** (quant.). ix–x. The sequential reaction of H_2_S and H_2_O_2_ with β**-*ribo*-4C** in water (pH 7) furnished β**-*ribo*-4U**

Having demonstrated the efficient conversion of **3A**, **3G** and **3I** to *arabino*-purines ***ara***-**4A**, ***ara***-**4G** and ***ara***-**4I**, respectively, we next investigated the reactivity of pyrimidine precursor **3C** under comparable conditions. Sutherland and co-workers reported that the reaction of α-*ribo*-anhydrocytidine (**α**-***ribo***-**3C**) with sodium hydrogen sulfide (NaHS) in formamide at 50 °C yields 2-thio-α-cytidine (**α**-***ribo***-**6C**; 84%; Fig. 4b.iv)^35^. Similarly, upon submitting **3C** to these conditions (NaHS, formamide, 50 °C), we observed conversion to ***ara***-**4C** (24%) and 2-thio-*arabino*-cytidine (***ara*-6C**; 31%) (Fig. 4a.iv; Supplementary Fig. 24). Conversely, incubation of **3C** (28 mM) with H_2_S (0.14 M) in water (pH 7, 60 °C) yielded mostly ***ara*-4C** (68%) alongside some conversion of ***ara*-4C** to 4-thio-*arabino*-uridine (***ara***-**7U**; 22%) after 2 d (Fig. 4a.i–ii; Supplementary Fig. 25); <5% ***ara*-6C** was observed. Further conversion of ***ara*-4C** to ***ara*-7U** was observed upon prolonged incubation (57:38 ***ara*-4C**/***ara*-7U** after 7 d; Fig. 4a.ii). Although in formamide the addition of NaHS to **3C** furnishes ***ara*-6C**, in water rapid hydrolysis of **3C** to ***ara*-4C** occurs, which subsequently undergoes slow nucleophilic substitution at the C4-carbon atom to furnish ***ara*-7U**.

The observed C4-thiolysis of ***ara*-4C** to ***ara*-7U** is not limited to the β-*arabino*-stereochemistry shown in Fig. 4a.ii. Submitting **α-*ribo*-3C** (28 mM) to the same aqueous thiolysis conditions afforded α-*ribo-*cytidine (**α**-***ribo*-4C**, 52%; Fig. 4b.ii) and 4-thio-α-*ribo-*uridine (**α**-***ribo*-7U**, 39%; Fig. 4b.ix). Incubation of ***ara*-4C**, **β**-***ribo*-4C** and **α**-***ribo*-4C** with H_2_S (pH 7) led to partial (unoptimised) conversion to the 4-thiouridines ***ara*-7U** (7 d, 40%), **β-*ribo*-7U** (7 d, 16%) and **α-*ribo*-7U** (8 d, 63%), respectively (Table 1), alongside recovered cytidine starting material [***ara***-**4C** (52%), **β-*ribo*-4C** (84%) and **α-*ribo*-4C** (37%)] (Supplementary Figs. 28–30). We were surprised to observe slower C4-thiolysis for the canonical *trans*-1′,2′-isomer (**β-*ribo*-4C**) than for the *cis*-1′,2′-isomers (**α**-***ribo*-4C** and ***ara*-4C**). Simultaneous thiolysis of 1:1 ***ara*-4C**/**β-*ribo*-4C** verified this rate difference; after 7 d more ***ara*-7U** (52%) than **β-*ribo*-7U** (18%) was observed (Supplementary Fig. 31), indicating the relative nucleobase/C2′-OH orientation affects the rate of nucleophilic substitution at the distal C4-position of cytidine nucleotides. Finally, we incubated pyrimidine **3C** and purine **3A** (1:1) with H_2_S (pH 7, 60 °C, 7 d). Gratifyingly, ***ara*-4C** (70%), ***ara*-7U** (30%) and ***ara*-5A** (71%) were cleanly furnished (Supplementary Fig. 27).

### Pyrimidine oxidation

An efficient protocol to convert ***ara*-7U** to ***ara*-4U** would indicate that nucleosides ***ara*-4C** and ***ara*-4U** could be readily generated in comparable yields from thiolysis of **3C** alongside conversion of **3A**/**3G** to ***ara*-4A**/***ara*-4G**, respectively. We envisioned that the thiocarbonyl moiety of ***ara*-7U** would be readily oxidised, however we suspected that oxidation would activate the pyrimidine nucleobase to hydrolysis (rather than reduction as observed for the purines). To test our hypothesis, we incubated ***ara*-7U** (50 mM), **β-*ribo*-7U** (50 mM) and α**-*ribo*-7U** (50 mM) with H_2_O_2_ (0.15 m) in water (pH 7, room temperature, 3.5 h). Each reaction proceeded smoothly to give the respective uridine (***ara*-4U** (78%); **β-*ribo*-4U** (78%); α**-*ribo*-4U** (93%); Table 1, Fig. 4 and Supplementary Figs. 38, 40–41), which validated our prediction. We next investigated thiolysis and selective hydrolysis in a one-pot reaction: **3C** (35.7 mM) was incubated with H_2_S (0.71 M) in water (pH 7, 60 °C, 7 d) and then H_2_O_2_ (375 µmol) was added. We observed concomitant formation of ***ara*-4C** (62%) and ***ara*-4U** (25%) (Supplementary Fig. 39).

The reactions of 2-thiocytidines ***ara*-6C** and α-***ribo*-6C** with H_2_O_2_ (150 mM) in water (pH 7, room temperature, 2 h) cleanly regenerated anhydrocytidines **3C** (82%; Fig. 4a.v) and α**-*ribo*-3C** (80%; Fig. 4b.viii), respectively, demonstrating a clear switch in reactivity relative to ***ara*-7U** due to the proximity of the C2′-hydroxyl and thiocarbonyl moieties. Therefore, it is of note that canonical cytidine **β-*ribo*-6C** has an *anti*-1′,2′-disposition, and we expected to observe hydrolysis during H_2_O_2_ oxidation of **β-*ribo*-6C**. As expected, incubation of **β-*ribo*-6C** (38.4 mM) with H_2_O_2_ (232 mM) in phosphate buffer (pH 7–9, room temperature, 7 h) afforded a quantitative conversion to **β-*ribo*-4C** (Fig. 4b.vi; Supplementary Fig. 44). Interestingly, buffering the oxidation at pH 3 with glycine afforded **β-*ribo*-4C** (24%) alongside 4-amino-pyrimidine-riboside (**β-*ribo*-9**; 76%; Fig. 4b.vii; Supplementary Fig. 44). It is likely that cytidine protonation switches on C2-reduction by promoting access to the carbene intermediate required for reduction (Fig. 4b.vii)^51^. This hypothesis is supported by the simultaneous oxidation of ***ara*-7U** and **β-*ribo*-6C** with H_2_O_2_ (pH 3, glycine buffer, room temperature), which affords **β-*ribo*-4C** (20%) and **β-*ribo*-9** (72%) from **β-*ribo*-6C**, but exclusively ***ara*-4U** (92%) from ***ara*-7U** (Supplementary Fig. 45).

It is particularly interesting, with respect to the origins of life, that purine C8-reduction is facile and quantitative at neutral pH, whereas pyrimidine C2-reduction is only observed at low pH. Consequently, the reaction of **β-*ribo*-6C** with H_2_O_2_ at neutral pH furnishes canonical **β-*ribo*-4C** cleanly, but protects against, and even reverses, the formation of non-canonical ***ara*-6C** and **α-*ribo*-6C**. This provides a facile and selective method to convert **β-*ribo*-6C**^35^ to canonical nucleoside **β-*ribo*-4C**.

### Concomitant purine and pyrimidine synthesis

**Fig. 5:**
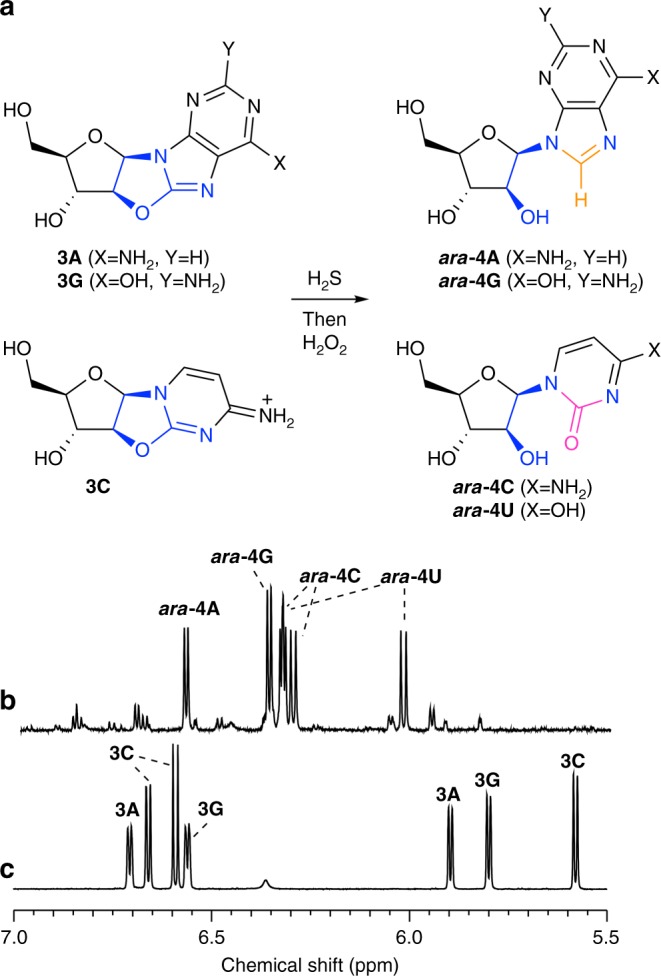
Concomitant synthesis of four Watson–Crick base-pairing nucleosides. **a** One-pot reaction of an equimolar mixture of anhydroadenosine (**3A**), anhydroguanosine (**3G**) and anhydrocytidine (**3C**) with H_2_S (20 equiv., pH 7) in water, then H_2_O_2_ (20 equiv., pH 7) in water affords adenosine ***ara*-4A** (53%), guanosine ***ara*-4G** (62%), cytidine ***ara*-4C** (47%) and uridine ***ara*-4U** (35%). The isourea moieties (blue) of the anhydropurines (**3A/G**) and anhydropyrimidine (**3C**) were differentiated in situ to afford the canonical Watson–Crick base-pairing nucleobase moieties regiospecifically on a furanosyl-sugar scaffold, by reduction (orange) and hydrolysis (magenta), respectively. **b** ^1^H NMR spectrum (600 MHz, 9:1 H_2_O/D_2_O, 25 °C, *δ* = 5.5–7.0 ppm) showing the prebiotic synthesis of cytidine ***ara*-4C**, uridine ***ara*-4U**, adenosine ***ara*-4A** and guanosine ***ara*-4G** following the sequential addition of H_2_S and H_2_O_2_ to anhydrocytidine **3C**, anhydroadenosine **3A** and anhydroguanosine **3G** in water. **c** ^1^H NMR spectrum (600 MHz, 9:1 H_2_O/D_2_O, 25 °C, *δ* = 5.5–7.0 ppm) of substrates anhydrocytidine **3C**, anhydroadenosine **3A** and anhydroguanosine **3G** in water

To form information-rich ANA nucleic acid oligomers, all four Watson–Crick base-pairing nucleosides (***ara*-4A**, ***ara*-4C**, ***ara*-4G** and ***ara*-4U**) need to accrue at the same time in the same environment, ideally from the same set of chemical reactions. We reacted 1:1:1 **3C**, **3A** and **3G** with H_2_S (pH 7, 60 °C; Supplementary Fig. 46), and after 7 d we observed ***ara*-5A** (65%), ***ara*-5G** (62%), ***ara*-4C** (55%) and ***ara*-7U** (35%). Subsequent incubation with H_2_O_2_ (pH 7, room temperature, 2 h) gave the desired Watson–Crick base-pairing products ***ara*-4A** (53%), ***ara*-4G** (62%), ***ara*-4C** (47%) and ***ara*-4U** (35%) without purification or isolation of intermediate products (Fig. 5). Thus, we achieved a plausibly prebiotic divergent synthesis of Watson–Crick base-pairing nucleosides, and have marked ANA as a likely candidate for the nucleic acid of early evolution.

## Discussion

Through the reaction of pyrimidine and purine precursors (**3C** and **3A**, respectively), which can be accessed divergently from a single prebiotic substrate^16,22^, we have elucidated an efficient plausibly prebiotic method to simultaneously form *arabino*-pyrimidine and *arabino*-purine nucleosides. Both the purine and pyrimidine nucleosides are delivered with regiospecific glycosylation on a furanose-specific arabinose sugar moiety. The reaction of H_2_S in water unlocks a purine-specific C8-reduction, by either UV irradiation or H_2_O_2_ oxidation; H_2_O_2_ oxidation is especially high yielding, however, UV irradiation caused selective destruction of ***ara*-5I**/**β-*ribo*-5I** over ***ara*-5A**/**β-*ribo*-5A** and ***ara*-5G**/**β-*ribo*-5G**. The remarkable purity of the *arabino*-purines delivered by either route is highly encouraging, but the direct mechanism for canonical purine nucleobases selection by UV light provides a mechanism (based in the physical properties of the purine bases) for nucleobase selection prior to their incorporation into nucleic acid biopolymers at the origins of life.

In water H_2_S also undergoes selective, but slow, addition to the cytidine C4-position, and—when coupled with oxidative hydrolysis—this provides a selective mechanism to convert cytidines to uridines. Importantly, H_2_S did not convert A/G to I/xanthine (X) demonstrating another remarkable, but essential, reactivity difference between the canonical nucleosides.

Although our focus has been on developing new chemistry, not on assessing geochemical boundary conditions, we note that the availability of H_2_S and H_2_O_2_ are both plausibly prebiotic, and although their sequential reaction implicates different redox states this is not geochemically implausible. The entire planet is at redox disequilibrium, and layered redox gradients are geochemically common. Outgassing volatile compounds (from Earth’s interior) are likely to have played a critical role in determining prebiotic chemistry, and local redox environments (for example, due to volcanic outgassing, meteorite impacts, photo-oxidation and atmospheric water dissociation and hydrogen escape) are expected to provide significant variation from the global average^52^. H_2_S is produced through a distinct mechanism from H_2_O_2_, thus discrete redox zones could be readily established. The oxidation state of early Earth (Hadean) magmas are not well constrained, however, zircons that pre-date the known rock record suggest average oxygen fugacities may have been similar to the present-day conditions^53^. Accordingly, sulfide-leaching and H_2_S-outgassing would have occurred on the early Earth, with sulfur outgassing rates as high as 10^11.5^ cm^−2^ s^−1^ possible during major volcanogenic emplacement of basaltic plains^54^. Equally, photo-dissociation generates H_2_O_2_ from water, and H_2_O_2_ is a likely key environmental oxidant prior to global oxidation^37,50,55^. It seems reasonable to suppose that distinct (redox) reactivity could be controlled by geochemical localisation and the different physicochemical processes that yield and accumulate feedstock molecules. Geochemical H_2_S outgassing and atmospheric H_2_O_2_ production are well suited to this specific localisation. Moreover, it has recently been proposed that H_2_O_2_ can accumulate within ices in an anoxic atmosphere^56^. Melting H_2_O_2_-rich ices could augment H_2_O_2_ delivery into aqueous environments and, in principle, could help to provide a mechanism for the sequential delivery of H_2_S and H_2_O_2_ into, for example, a flowing stream system or pool. Although the reactions of H_2_S and H_2_O_2_ must occur in sequence to achieve purine reduction by the described oxidative mechanism, it is of note that the purine anhydronucleotides (**3A** and **3G**) were observed to be stable to H_2_O_2_ oxidation and the reduced purines (***ara***-**4A** and ***ara***-**4G**) were observed to be stable to H_2_S addition. Therefore, cycling material between these redox conditions (or across this redox gradient) is not considered to be problematic for this chemistry; indeed, a one-pot two-step H_2_S/H_2_O_2_ reduction has been demonstrated. Importantly, photochemical mercaptopurine reduction does not require two redox states, only H_2_S and UV light (at *λ* = 250–300 nm), both of which are expected to be in adequate (simultaneous) supply on the early Earth when sulfur outgassing rates are less than 10^11.5^ cm^−2^ s^−1^. The localised enhancement in H_2_S can be achieved in surface hydrothermal systems with shallow water reservoirs (to allow UV penetration)^54^. In terms of the UV-light dose environment, we note that in the laboratory only six of the available sixteen lamps in the Rayonet irradiation chamber were employed, which corresponds to 39,000 erg s^−1^ cm^−2^ and 30,900 erg s^−1^ cm^−2^ at *λ* = 254 nm and 300 nm, respectively. The integrated surface flux (*λ* = 200–300 nm) delivered to the Earth by the early Sun is estimated to be about 2700 erg s^−1^ cm^−2^ (and within roughly an order of magnitude of the experimental apparatus), which provides ample flux to achieve the desired transformations and preserve prebiotic plausibility^39^. Crucially, the two distinct mercaptopurine **5** reductions demonstrate, not only the specific value of sulfur in prebiotic nucleoside synthesis, but also, more generally, the value of chemical redundancy. Two mechanisms for mercaptopurine reduction that operate under different conditions but both furnish the same purine products renders the overall transformation more robust, which may be especially important to consider in the context of the origins of life. Chemical redundancy is highly likely to improve pathway or network robustness, which may be an essential feature of sustained proto-metabolism in a (geo)chemically fluctuating environment^3,18^.

The different reactions observed between **3C** and **3A/G** with H_2_S is ideally suited to concomitant synthesis of the canonical nucleobases on preformed sugar scaffolds. Although the *arabino*-stereochemistry is not found in extant genetics, it is highly plausible that ANA could have been a precursor to RNA in early life or that early co-evolution of mixed RNA/ANA systems could have been superseded by RNA/DNA systems. The simplicity and efficiency of the synthesis of A, C, G and U arabinosides indicates that further investigations into the synthesis of **3G** and the potential for ANA and RNA co-evolution are both warranted.

## Methods

### General procedure A. Thiolysis

Sodium hydrosulfide or disodium sulfide nonahydrate (20 equiv.) was dissolved in H_2_O/D_2_O (9:1) at pH 7. Nucleoside(s) (1 equiv.) was added and incubated at pH 7 and 60 °C. The reaction progress was monitored periodically by NMR spectroscopy. Upon completion, the reaction was cooled to room temperature and sparged of hydrogen sulfide with nitrogen or argon gas. The solution was adjusted to pH 6.5 and analysed by NMR spectroscopy.

### General procedure B. UV irradiation

A degassed aqueous solution of nucleoside(s) (2.00 mM, pH 6.5) was irradiated in a Rayonet reactor (SNE Ultraviolet Co.) housing six RPR-2537A or RPR-3000A lamps (with principal emission at *λ* = 254 nm and *λ* = 300 nm, respectively) at 38 °C under an argon atmosphere. After irradiation, the reaction was allowed to cool to room temperature, and then lyophilised. The lyophilisate was dissolved in D_2_O (500 µL) and analysed by NMR spectroscopy. A solution of potassium hydrogen phthalate (0.100 M, 50.0 µL, 5.00 µmol in D_2_O) was added as an internal NMR standard and NMR spectra were reacquired.

### General procedure C. Nucleoside oxidation

Nucleoside(s) (1.0 equiv., 50 mM) and potassium hydrogen phthalate (0.2 equiv.) were dissolved in H_2_O/D_2_O (9:1) or buffer solution. The pH of the solution was adjusted to pH 7 and NMR spectra were acquired. Hydrogen peroxide (30% w/w solution in H_2_O) was added and NMR spectra were periodically acquired at pH 7.

### Computational methods

The vertical excitation energies, excited state geometries and excited state harmonic vibrational frequencies of ***ara*-5I**, ***ara*-5A** and ***ara*-5G** were computed by using the algebraic diagrammatic construction to the second-order method [ADC(2)]^40,41,57^, and the cc-pVTZ basis set. The MP2/cc-pVTZ method was used to optimise the corresponding ground-state geometries. Spin–orbit coupling matrix elements were calculated at the CASPT2/SA-CASSCF(10,9)/cc-pVTZ-DK level^58^, including the second-order Douglas–Kroll–Hess transformation to account for scalar relativistic effects. Calculated ESA spectra were obtained by convolution of vertical excitation energies and oscillator strengths with normalised Gaussian functions (0.20 eV half-width). Vertical excitation energies necessary for the ESA spectra were calculated from the corresponding excited state minima and 11 excitations were taken into account in each case. ESA cross-sections were generated using the GaussSum programme^59^. Minimum-energy crossing point (MECPs) geometries were optimised with the in-house implementation of the method proposed by Levine, Coe and Martinez^60^. The energies and gradients in electronically excited states were computed at the ADC(2) level, whereas the corresponding properties for electronic ground states were obtained at the MP2 level in the MECP geometry optimisations^41^. The MECP optimisation steps were performed with the Broyden–Fletcher–Goldfarb–Shanno quasi-Newton scheme available in the internal optimiser of Turbomole 7.1^61^. All ADC(2) and MP2 calculations were performed with Turbomole 7.1, and Molcas 8.0^62^ was employed for all the CASPT2/SA-CASSCF calculations. Graphical representations of the molecular geometries and orbitals were generated with IboView^63^.

### Femtosecond transient absorption spectroscopy

FTAS experiments were performed with a Solstice Ace pulsed laser system (Spectra-Physics, Newport Co.), which produces 97 fs pulses at *λ* = 800 nm. To generate the white light continuum probe pulses (*λ* = 320–700 nm) in the Helios-Fire spectrometer (Ultrafast Systems, LLC), a fraction of the fundamental beam was focused on a thin CaF_2_ crystal. The *λ* = 290 nm excitation (pump) pulses were generated by passing the remainder of the fundamental beam through an optical parametric amplifier (TOPAS, Light Conversion, Ltd.). FTAS experiments of aqueous samples of ***ara***-**5A** (4 mM, pH 7.4) and ***ara***-**5I** (4 mM, pH 7.4) were performed in 2 mm optical path length quartz cuvettes (Starna Cells, Inc.). LabView Surface Xplorer software (Ultrafast Systems, LLC) was used to reduce the FTAS data, apply corrections for group velocity dispersion of the white light probe, and extract and analyse spectra and their time dependence.

### X-ray diffraction

All diffraction data were collected by using a four-circle Agilent SuperNova (Dual Source) single crystal X-ray diffractometer with a micro-focus CuK_α_ X-ray beam (*λ* = 1.54184 Å) and an Atlas CCD detector. The crystal temperature was controlled by using an Oxford Instruments Cryojet5. Unit cell determination, data reduction and analytical numeric absorption correction using a multifaceted crystal were carried out using the CrysAlisPro programme^64^. The crystal structures were solved with the ShelXS programme and refined by least squares on the basis of F^2^ with the ShelXL programme^65^. All non-hydrogen atoms were refined anisotropically by the full-matrix least-squares method. Hydrogen atoms affiliated with oxygen and nitrogen atoms were refined isotropically in positions identified by the difference Fourier map, or in geometrically constrained positions. Hydrogen atoms associated with carbon atoms were refined isotropically in geometrically constrained positions.

## Electronic supplementary material

Supplementary Information

Peer Review File

## Data Availability

The authors declare that data supporting the findings of this study are available within the paper and its Supplementary Information files and figures. X-ray crystallographic data were deposited at the Cambridge Crystallographic Data Centre (CCDC) under the following CCDC deposition numbers: 1586272 (***ara*****-5A**, Supplementary Fig. 120) and 1836032 (3′,3-anhydro-guanosine (**12**); the high pH isomerisation product of **3G**, Supplementary Fig. 121). These can be obtained free of charge from CCDC via https://www.ccdc.cam.ac.uk/structures/.
