# Supplementary material for: Selective prebiotic conversion of pyrimidine and purine anhydronucleosides into Watson-Crick base-pairing arabino-furanosyl nucleosides in water
Source: Nat Commun. 2018 Oct 4;9:4073. doi: 10.1038/s41467-018-06374-z (PMC6172253; doi:10.1038/s41467-018-06374-z)
Supplement: Supplementary file 1 — Supplementary Information [file 41467_2018_6374_MOESM1_ESM.pdf]

Supplementary Information for

Selective Prebiotic Conversion of Pyrimidine and Purine Anhydronucleosides into Watson-Crick Base Pairing *arabino*-Furanosyl Nucleosides in Water

Roberts et al.

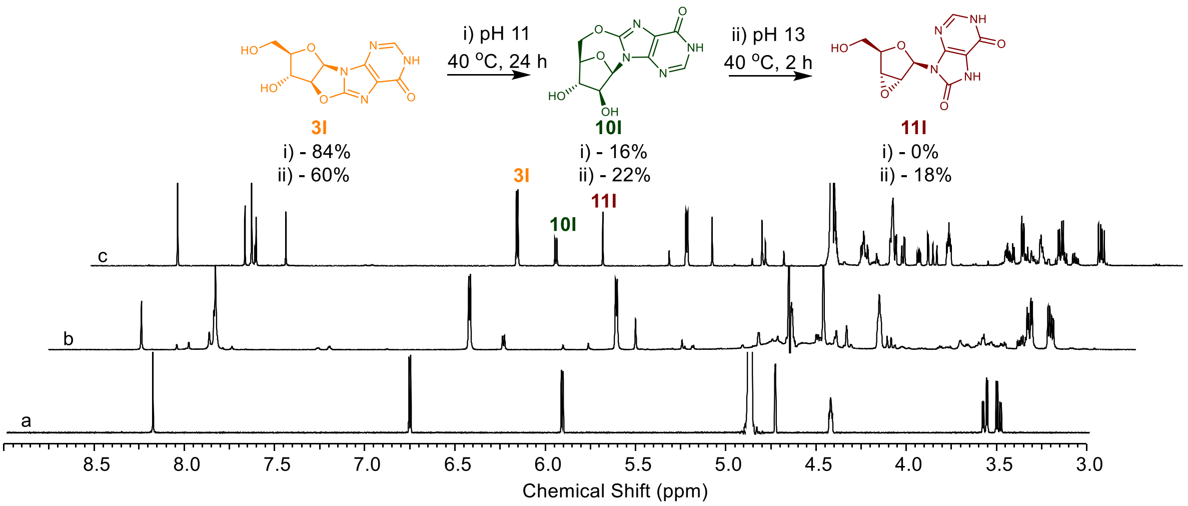

Supplementary Figure 1 | ^{1}H NMR spectra showing the isomerisation of 2′,8-anhydroinosine 3I (37.6 mM) in alkaline solution at 40 °C. a. *^{1}H NMR (600 MHz, {D_2O}, 3.0–9.0 ppm) spectrum showing the reaction starting material.* ***b.*** *^{1}H NMR (600 MHz, {H_2O/D_2O, 9:1}, noesygppr1d, 3.0–9.0 ppm) spectrum showing the products of* ***3I*** *(37.6 mM) incubated at pH 11 and 40 °C for 24 h.* ***c.*** *Spectrum showing the isomerisation products (spectrum b) following incubation at pH 13 and 40 °C for 2 h.*

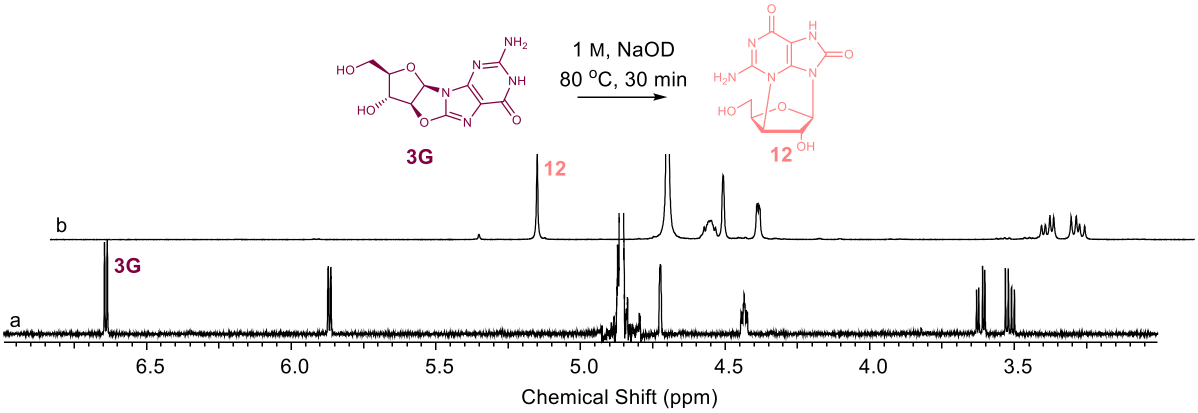

Supplementary Figure 2 | ^{1}H NMR (400 MHz, {D_2O}, 3.0–7.0 ppm) spectra showing the isomerisation of 2′,8-anhydroguanosine 12 (37.6 mM) in NaOD (1 M, D_2O) at 80 ℃ after 30 min

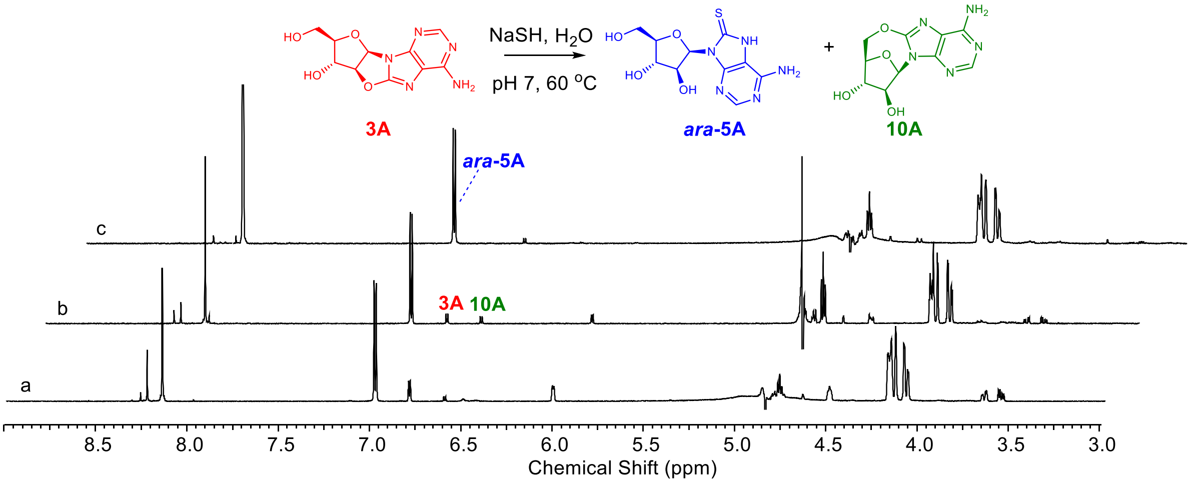

Supplementary Figure 3 | ^{1}H NMR (600 MHz, {H_2O/D_2O 9:1}, noesygppr1d, 3.0–9.0 ppm) spectra showing the reaction of 2′,8-anhydroadenosine 3A (35.7 mM) and NaSH (714 mM) at pH 7 and 60 °C over 7 d. a. *Spectrum showing the reaction products after 2 d.* ***b.*** *Spectrum showing the reaction products after 3 d.* ***c.*** *Spectrum showing the reaction products after 7 d.*

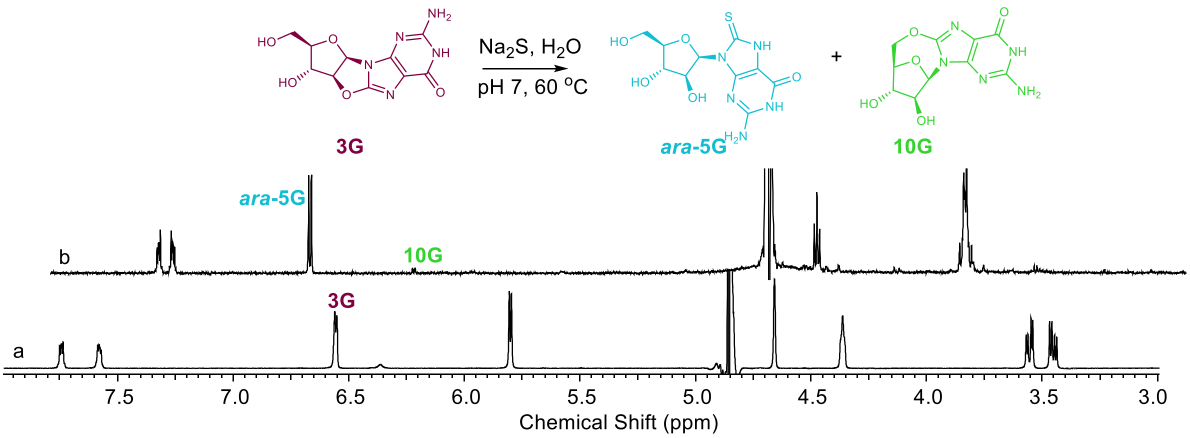

Supplementary Figure 4 | ^{1}H NMR (600 MHz, {H_2O/D_2O 9:1}, noesygppr1d, 3.0–8.0 ppm) spectra showing the reaction of 2′,8-anhydroguanosine 3G (35.7 mM) and potassium hydrogen phthalate 13 (7.14 mM; internal NMR standard) with Na_2S (714 mM) at pH 7 and 60 °C. a. *Spectrum showing the reaction starting materials.* ***b.*** *Spectrum showing the reaction products after 7d.*

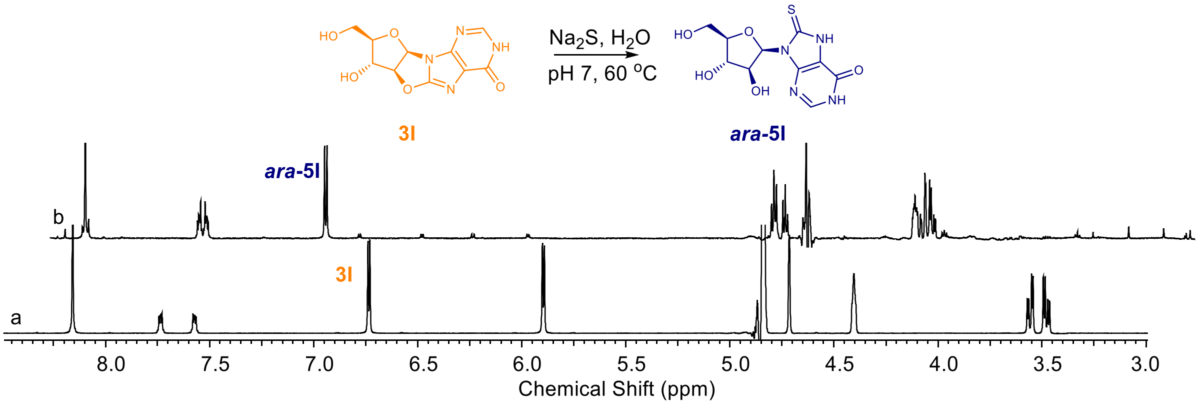

Supplementary Figure 5 | ^{1}H NMR (600 MHz, {H_2O/D_2O 9:1}, noesygppr1d, 3.0–8.5 ppm) spectra showing the reaction of 2′,8-anhydroinosine 3I (35.7 mM) and potassium hydrogen phthalate 13 (7.14 mM; internal NMR standard) with Na_2S (714 mM) at pH 7 and 60 °C for 7 d. a. *Spectrum showing the reaction starting materials.* ***b.*** *Spectrum showing the reaction products after 7 d.*

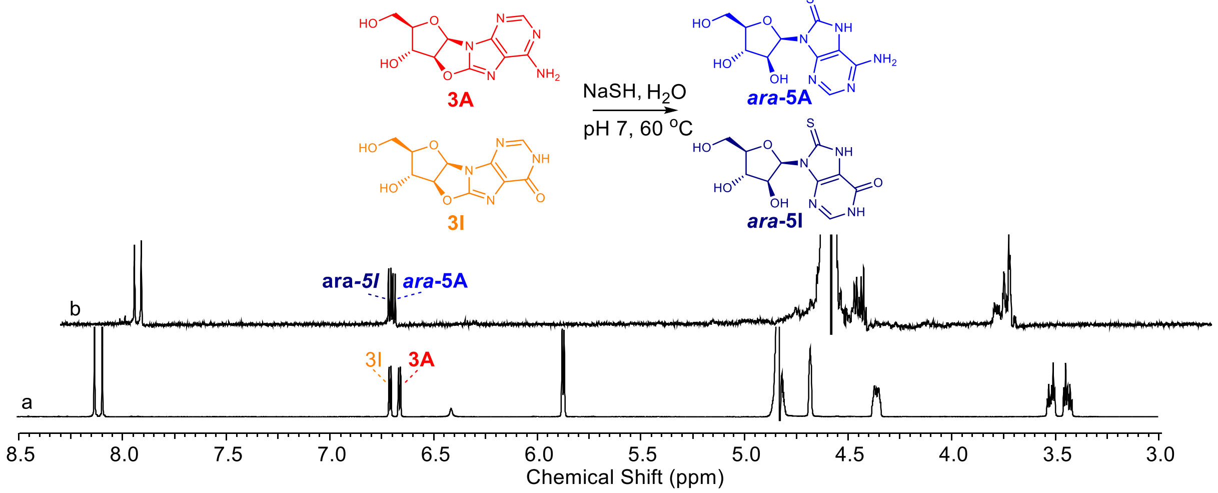

Supplementary Figure 6 | ^{1}H NMR (600 MHz, {H_2O/D_2O, 9:1}, noesygppr1d, 3.0–8.5 ppm) spectra showing the reaction of 2′,8-anhydroadenosine 3A (2.93 mM) and 2′,8-anhydroinosine 3I (2.93 mM) with NaSH (157 mM) at pH 7 and 60 °C for 7 d. a. *Spectrum showing the reaction starting materials.* ***b.*** *Spectrum showing the reaction products after 7 d.*

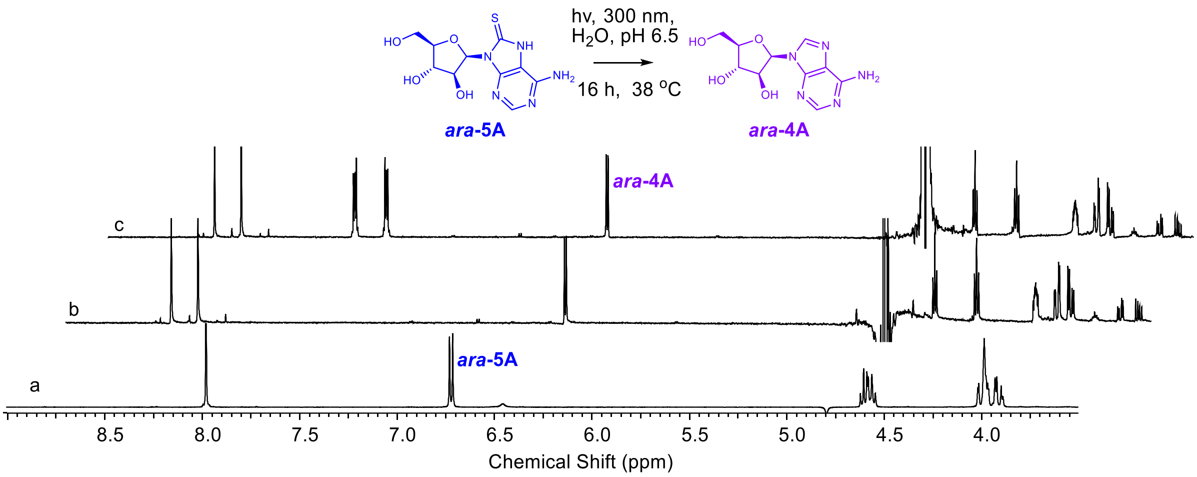

Supplementary Figure 7 | ^{1}H NMR spectra showing the irradiation (300 nm) at pH 6.5 and 38 °C of 8-mercapto-arabino-adenosine ara-5A (2 mM) for 16 h. a. *^{1}H NMR (600 MHz, {D_2O}, 3.5–9.0 ppm) spectrum showing the starting material.* ***b.*** *^{1}H NMR (600 MHz, {H_2O/D_2O, 9:1}, noesygppr1d, 3.5–9.0 ppm) spectrum showing the irradiation products after 16 h.* ***c.*** *Spectrum showing the irradiation products (spectrum b) after spiking with potassium hydrogen phthalate* ***13*** *(5.00 µmol; internal NMR standard).*

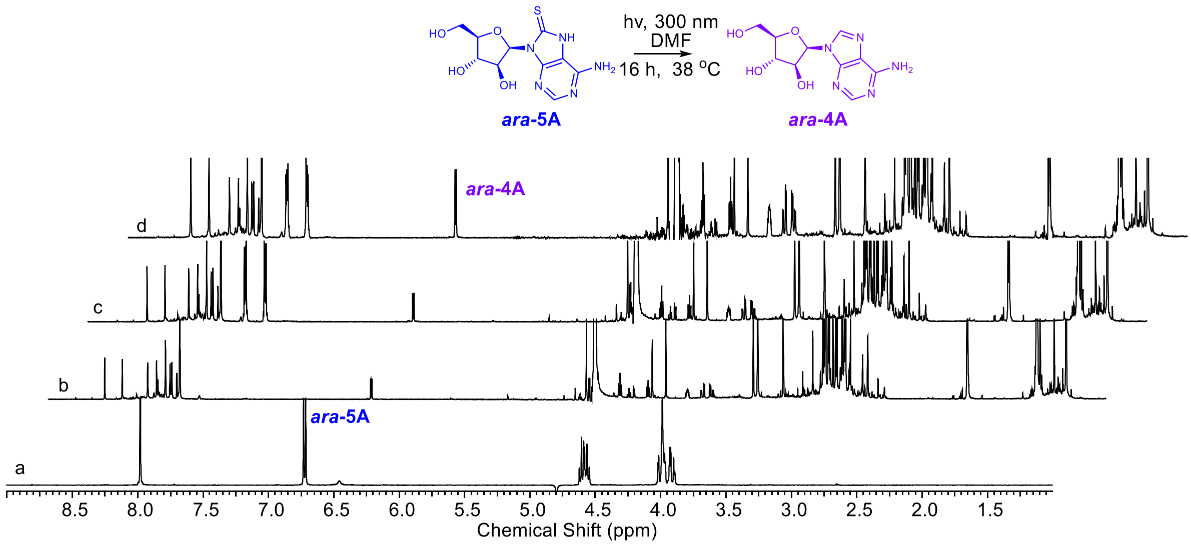

Supplementary Figure 8 | ^{1}H NMR (600 MHz, {H_2O/D_2O, 9:1}, noesygppr1d, 1.0–9.0 ppm) spectra showing the irradiation (300 nm) at 38 °C of 8-mercapto-arabino-adenosine ara-5A (2 mM) in DMF for 16 h. a. *Spectrum showing the starting material.* ***b.*** *Spectrum showing the irradiation products after 16 h.* ***c.*** *Spectrum showing the irradiation products (spectrum b) after spiking with potassium hydrogen phthalate* ***13*** *(5.00 µmol; internal NMR standard).* ***d.*** *Spectrum showing the irradiation products (spectrum c) after spiking with arabino-adenosine* ***ara-4A****.*

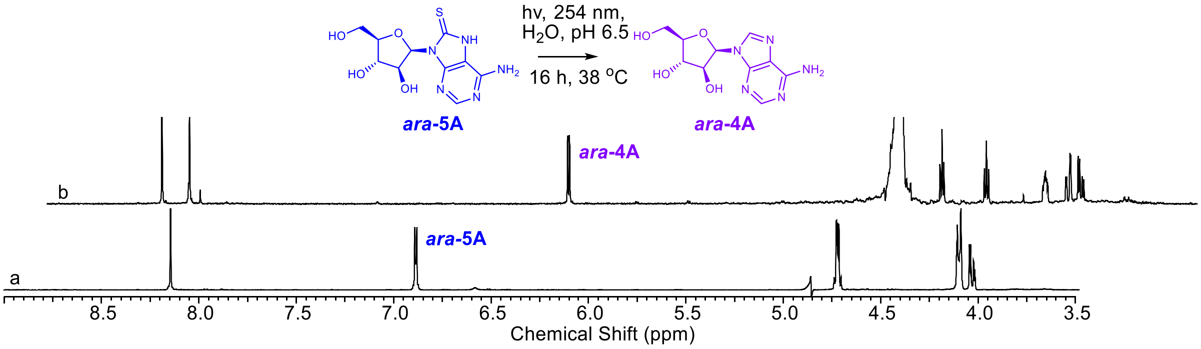

Supplementary Figure 9 | ^{1}H NMR (600 MHz, {D_2O}, noesygppr1d, 3.5–9.0 ppm) spectra showing the irradiation (254 nm) at pH 6.5 and 38 °C of 8-mercapto-arabino-adenosine ara-5A (2 mM) for 16 h in an Ace Glassware apparatus. a. *Spectrum showing the irradiation starting materials.* ***b.*** *Spectrum showing the irradiation products after 16 h.*

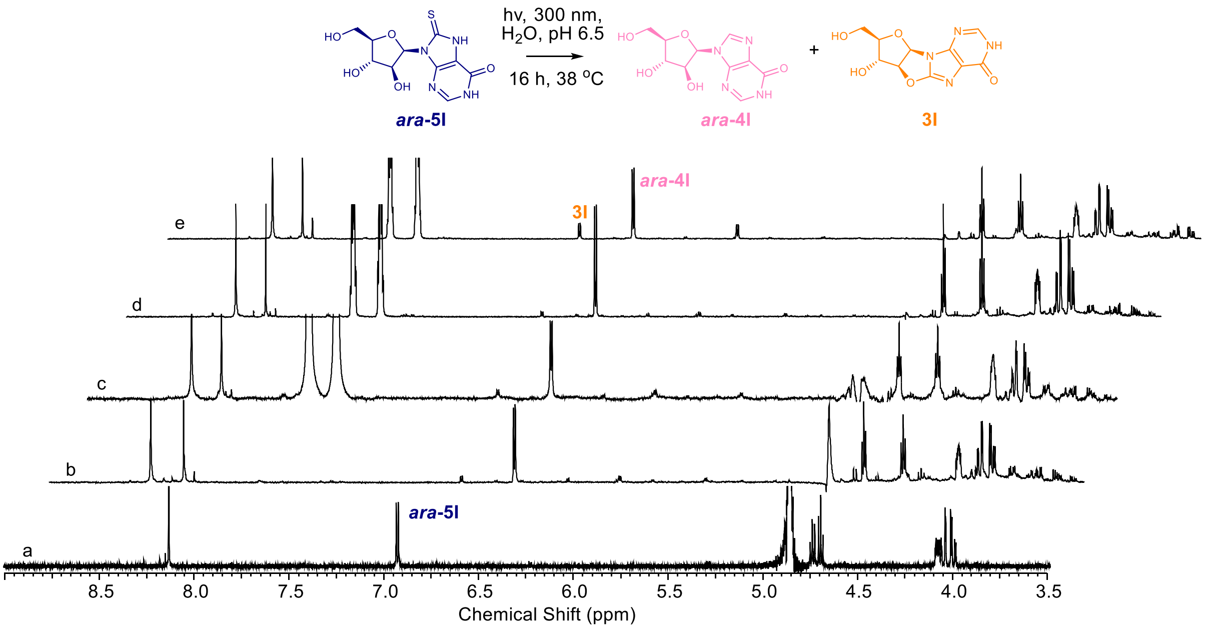

Supplementary Figure 10 | ^{1}H NMR spectra showing the irradiation (300 nm) at pH 6.5 and 38 °C of 8-mercapto-arabino-inosine ara-5I (2.00 mM) for 16 h. a. *^{1}H NMR (600 MHz, {D_2O}, 3.5–9.0 ppm) spectrum showing the starting material.* ***b.*** *^{1}H NMR (600 MHz, {D_2O}, noesygppr1d, 3.5–9.0 ppm) spectrum showing the irradiation products after 16 h.* ***c.*** *Spectrum showing the irradiation products (spectrum b) after spiking with potassium hydrogen phthalate* ***13*** *(5.00 µmol; internal NMR standard).* ***d.*** *Spectrum showing the irradiation products (spectrum c) after spiking with arabino-inosine* ***ara-4I***. ***e.*** *Spectrum showing the irradiation products (spectrum d) after spiking with 2′,8-anhydroinosine* ***3I***.

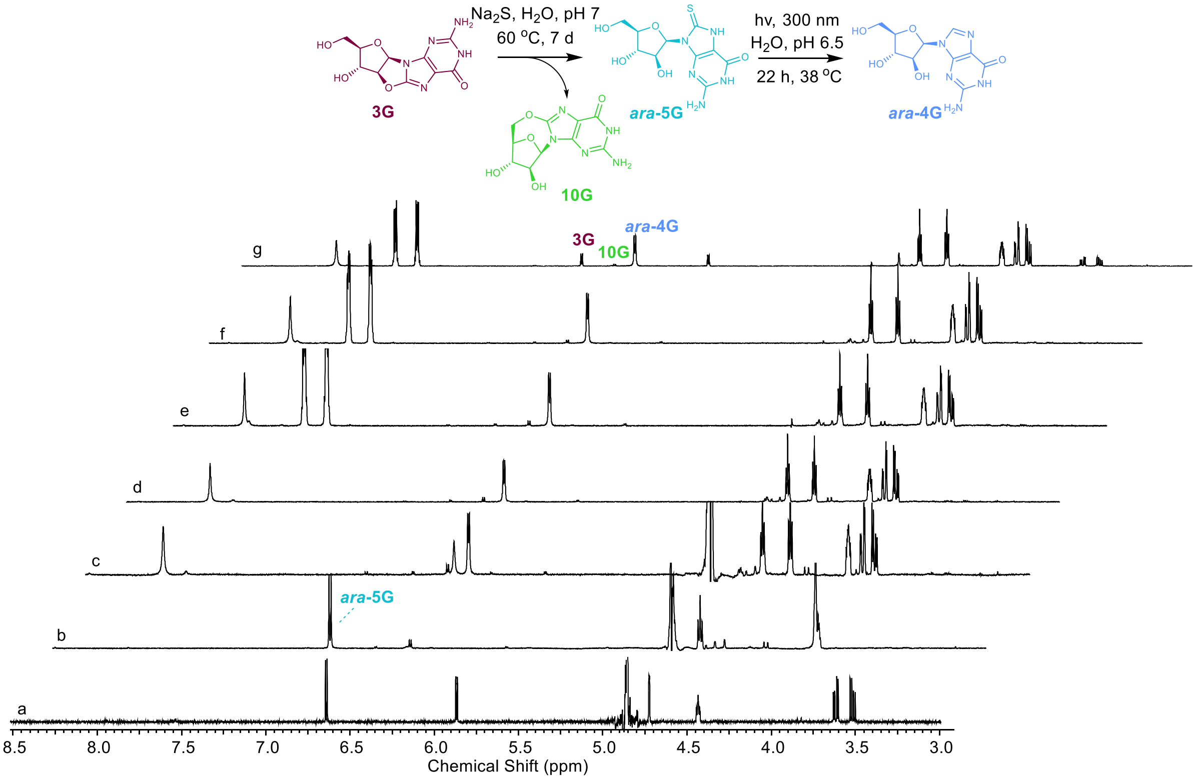

Supplementary Figure 11 | ^{1}H NMR (600 MHz, {D_2O}, noesygppr1d, 3.0–8.5 ppm) spectra showing the sequential reaction of 2′,8-anhydroguanosine 3G (5.85 mM) and Na_2S (157 mM) at pH 7 and 60 °C for 7 d followed by irradiation (300 nm) at pH 6.5 and 38 °C for 22 h. a. *Spectrum showing the reaction starting materials.* ***b.*** *Spectrum showing the thiolysis products after 7 d.* ***c.*** *Spectrum showing thiolysis products following irradiation for 16 h.* ***d.*** *Spectrum showing thiolysis products following irradiation for 22 h.* ***e.*** *Spectrum showing irradiation products (spectrum d) after spiking with potassium hydrogen phthalate* ***13*** *(5.00 µmol; internal NMR standard).* ***f.*** *Spectrum showing irradiation products (spectrum e) after spiking with arabino-guanosine* ***ara-4G***. ***g.*** *Spectrum showing irradiation products (spectrum e) after spiking with 2′,8-anhydroguanosine* ***3G***.

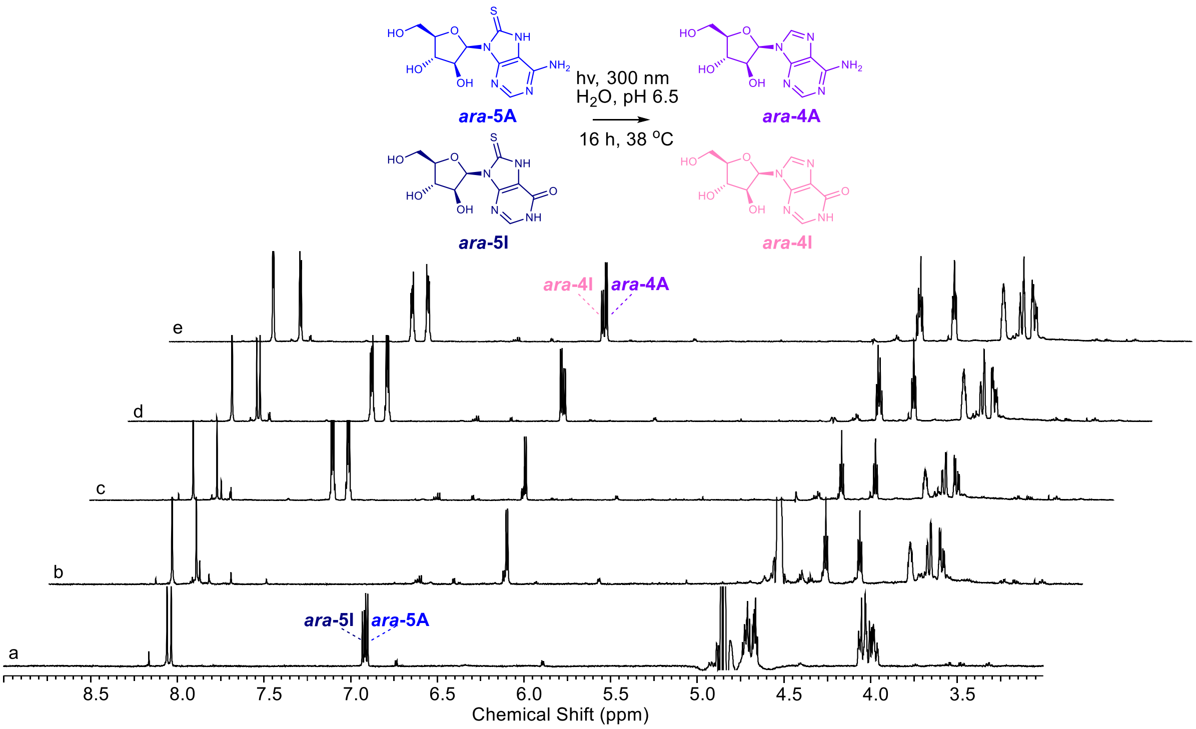

Supplementary Figure 12 | ¹H NMR spectra showing the simultaneous irradiation (300 nm) at pH 6.5 and 38 °C of 8-mercapto-arabino-inosine ara-5I (1 mM) and 8-mercapto-arabino-adenosine ara-5A (1 mM) for 16 h. a. *¹H NMR (600 MHz, {D₂O}, noesygppr1d, 3.0–9.0 ppm) spectrum showing the irradiation starting materials.* ***b.*** *¹H NMR (600 MHz, {H₂O/D₂O, 9:1}, noesygppr1d, 3.0–9.0 ppm) spectrum showing the irradiation products after 16 h.* ***c.*** *Spectrum showing the irradiation products (spectrum b) after spiking with potassium hydrogen phthalate* ***13*** *(5.00 µmol; internal NMR standard).* ***d.*** *Spectrum showing the irradiation products (spectrum c) after spiking with arabino-inosine* ***ara-4I****.* ***e.*** *Spectrum showing the irradiation products (spectrum d) after spiking with arabino-adenosine* ***ara-4A****.*

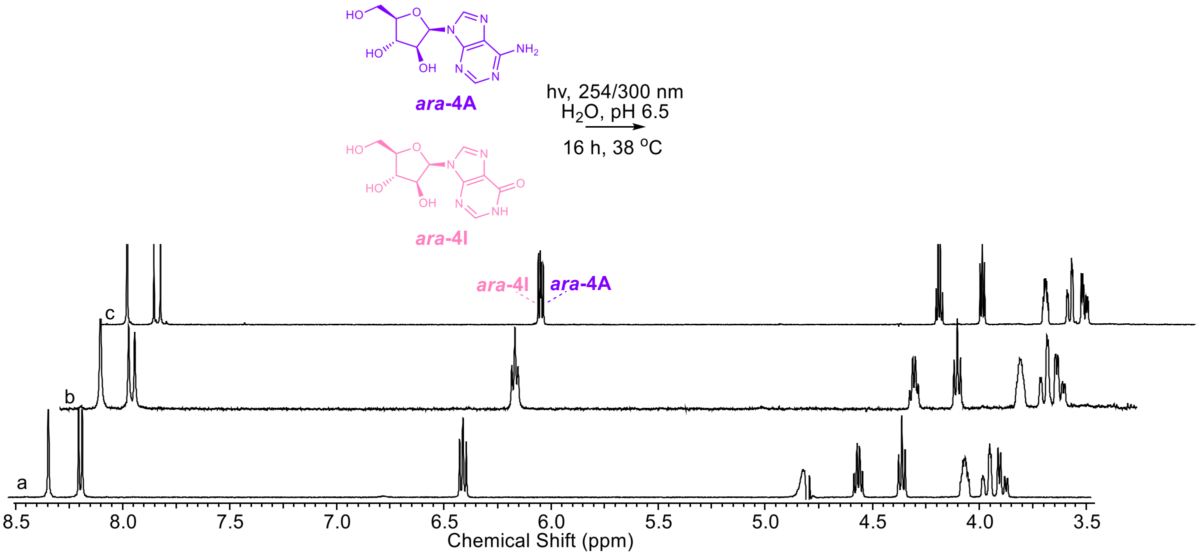

Supplementary Figure 13 | ¹H NMR (600 MHz, {H₂O/D₂O, 9:1}, noesygppr1d, 3.5–8.5 ppm) spectra showing the simultaneous irradiation (254 nm or 300 nm) at pH 6.5 and 38 °C of arabino-inosine ara-4I (1 mM) and arabino-adenosine ara-4A (1 mM) for 16 h. a. *Spectrum showing the starting material.* ***b.*** *Spectrum showing irradiation (254 nm) products after 16 h.* ***c.*** *Spectrum showing irradiation (300 nm) products after 16 h.*

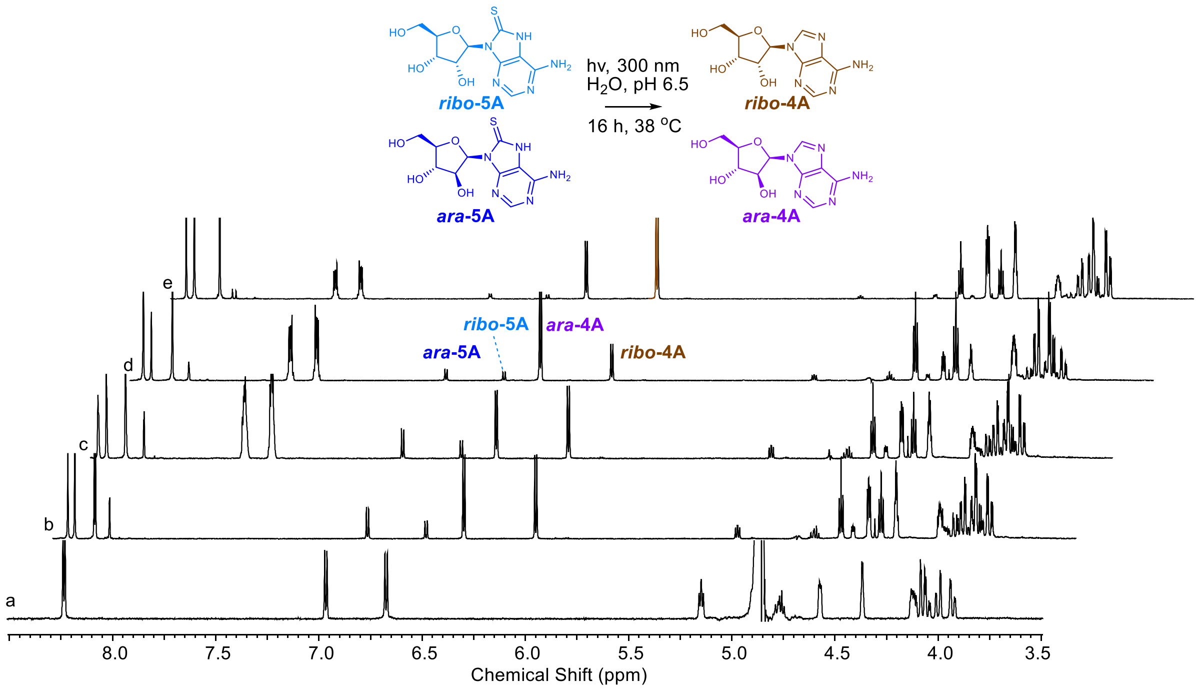

Supplementary Figure 14 | ^{1}H NMR spectra showing the simultaneous irradiation (300 nm) at pH 6.5 and 38 °C of 8-mercapto-arabino-adenosine ara-5A (1 mM) and 8-mercapto-ribo-adenosine ribo-5A (1 mM) for 16 h. a. *^{1}H NMR (600 MHz, {H_2O/D_2O, 9:1}, noesygppr1d, 3.5–8.5 ppm) spectrum showing the starting materials.* ***b.*** *^{1}H NMR (600 MHz, {D_2O}, noesygppr1d, 3.5–8.5 ppm) spectrum showing the irradiation products after 16 h.* ***c.*** *Spectrum showing the irradiation products (spectrum b) after spiking with potassium hydrogen phthalate* ***13*** *(5.00 µmol; internal NMR standard).* ***d.*** *Spectrum showing the irradiation products (spectrum c) after spiking with arabino-adenosine* ***ara-4A****.* ***e.*** *Spectrum showing the irradiation products (spectrum d) after spiking with ribo-adenosine* ***ribo-4A****.*

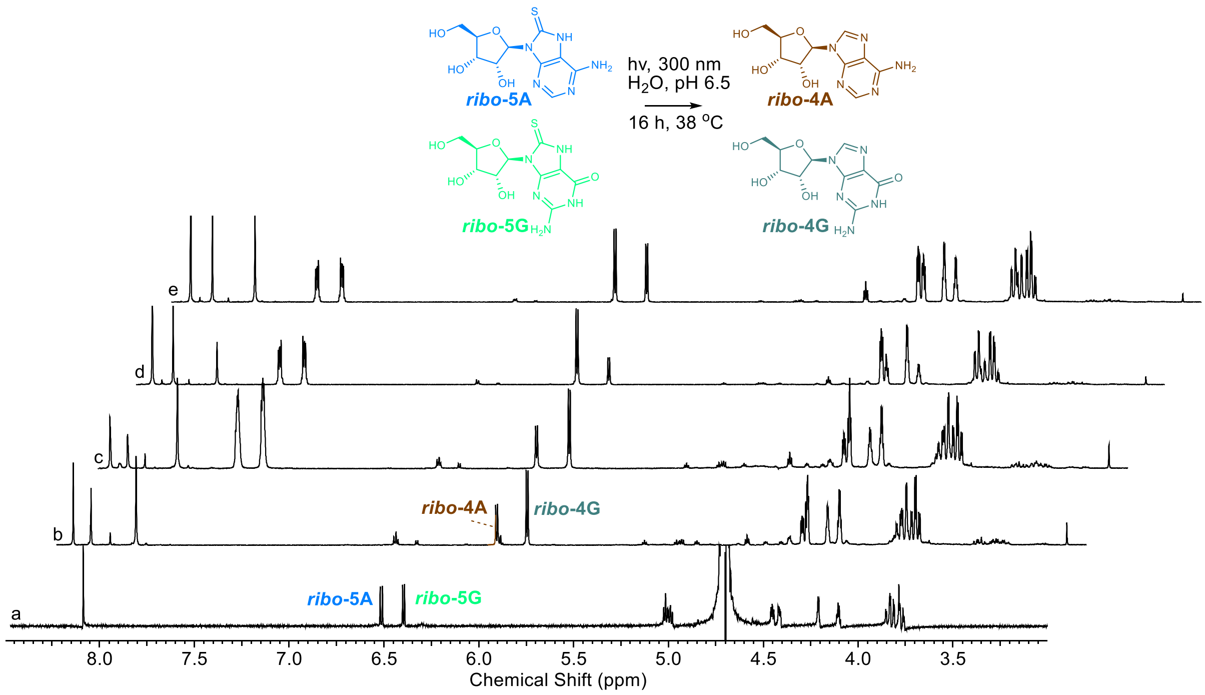

Supplementary Figure 15 | ^{1}H NMR spectra showing the simultaneous irradiation (300 nm) at pH 6.5 and 38 °C of 8-mercapto-ribo-guanosine ribo-5G (1 mM) and 8-mercapto-ribo-adenosine ribo-5A (1 mM) for 16 h. a. *^{1}H NMR (600 MHz, {H_2O/D_2O, 9:1}, noesygppr1d, 3.0–8.5 ppm) spectrum showing the starting materials.* ***b.*** *^{1}H NMR (600 MHz, {D_2O}, noesygppr1d, 3.0–8.5 ppm) spectrum showing the irradiation products after 16 h.* ***c.*** *Spectrum showing the irradiation products (spectrum b) after spiking with potassium hydrogen phthalate* ***13*** *(5.00 µmol; internal NMR standard).* ***d.*** *Spectrum showing the irradiation products (spectrum c) after spiking with ribo-adenosine* ***ribo-4A****.* ***e.*** *Spectrum showing the irradiation products (spectrum d) after spiking with ribo-guanosine* ***ribo-4G****.*

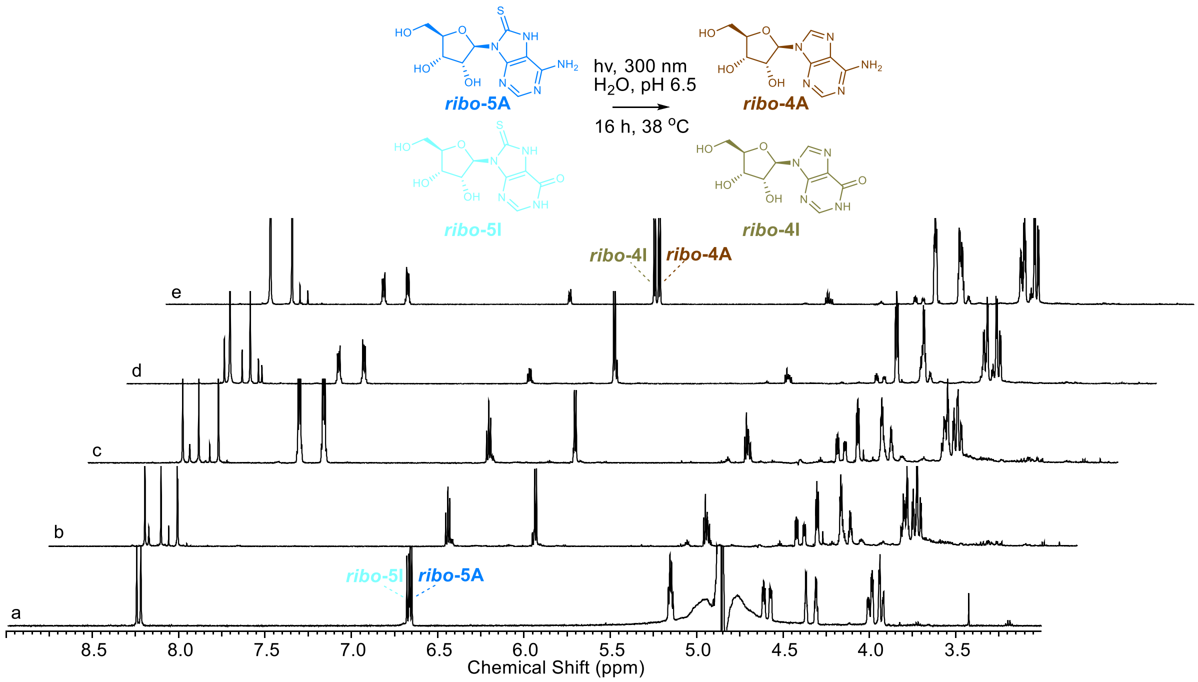

Supplementary Figure 16 | ^{1}H NMR spectra showing the simultaneous irradiation (300 nm) at pH 6.5 and 38 °C of 8-mercapto-ribo-inosine ribo-5I (1 mM) and 8-mercapto-ribo-adenosine ribo-5A (1 mM) for 16 h. a. *^{1}H NMR (600 MHz, {H_2O/D_2O, 9:1}, noesygppr1d, 3.0–9.0 ppm) spectrum showing the starting materials.* ***b.*** *^{1}H NMR (600 MHz, {D_2O}, noesygppr1d, 3.0–9.0 ppm) spectrum showing the irradiation products after 16 h.* ***c.*** *Spectrum showing the irradiation products (spectrum b) after spiking with potassium hydrogen phthalate* ***13*** *(5.00 µmol; internal NMR standard).* ***d.*** *Spectrum showing the irradiation products (spectrum c) after spiking with ribo-inosine* ***ribo-4I****.* ***e.*** *Spectrum showing the irradiation products (spectrum d) after spiking with ribo-adenosine* ***ribo-4A****.*

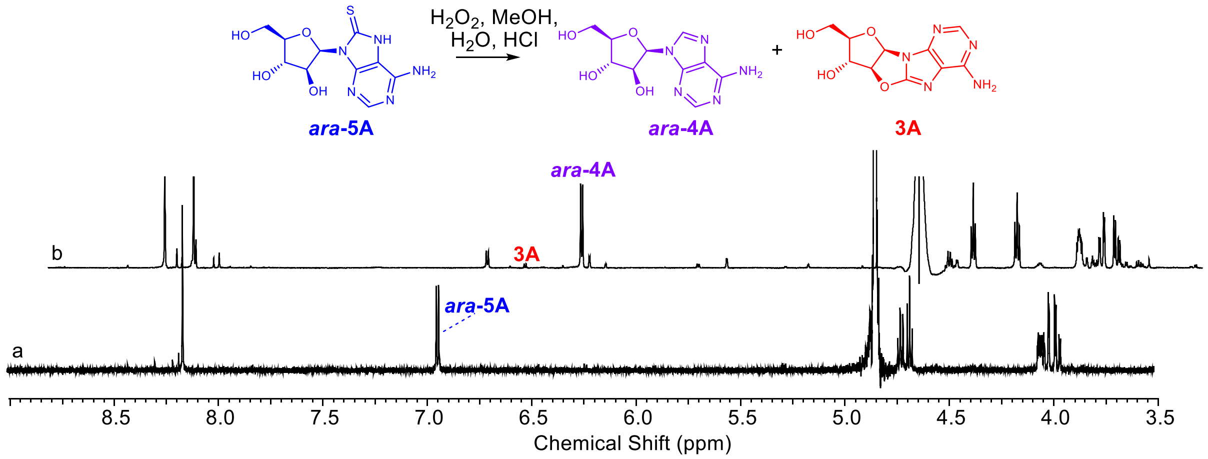

Supplementary Figure 17 | ^{1}H NMR spectra showing the reaction of 8-mercapto-arabino-adenosine ara-5A (40.0 mM) with H_2O_2 (157 mM) in H_2O, MeOH and HCl (4.00 µM) at room temperature. a. *^{1}H NMR (600 MHz, {D_2O}, 3.5–9.0 ppm) spectrum showing the reaction starting material.* ***b.*** *^{1}H NMR (600 MHz, {H_2O/D_2O, 9:1}, noesygppr1d, 3.5–9.0 ppm) spectrum showing the reaction products after 16 h.*

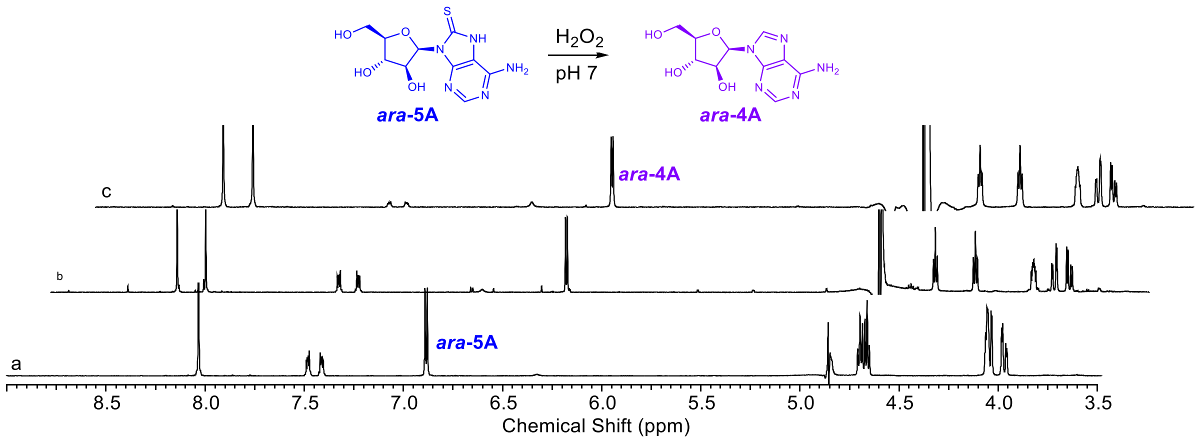

Supplementary Figure 18 | ^1H NMR (600 MHz, {H_2O/D_2O, 9:1}, noesygppr1d, 3.5–9.0 ppm) spectra showing the reaction of 8-mercapto-arabino-adenosine ara-5A (50.0 mM) and potassium hydrogen phthalate 13 (10.0 mM; internal NMR standard) with H_2O_2 (150 mM) at pH 7 and room temperature. a. *Spectrum showing the reaction starting material.* ***b.*** *Spectrum showing the reaction products after 2 h then being adjusted to pH 7 and incubated for 1 h further at room temperature.* ***c.*** *Spectrum showing reaction products (spectrum b) after spiking with arabino-adenosine* ***ara-4A****.*

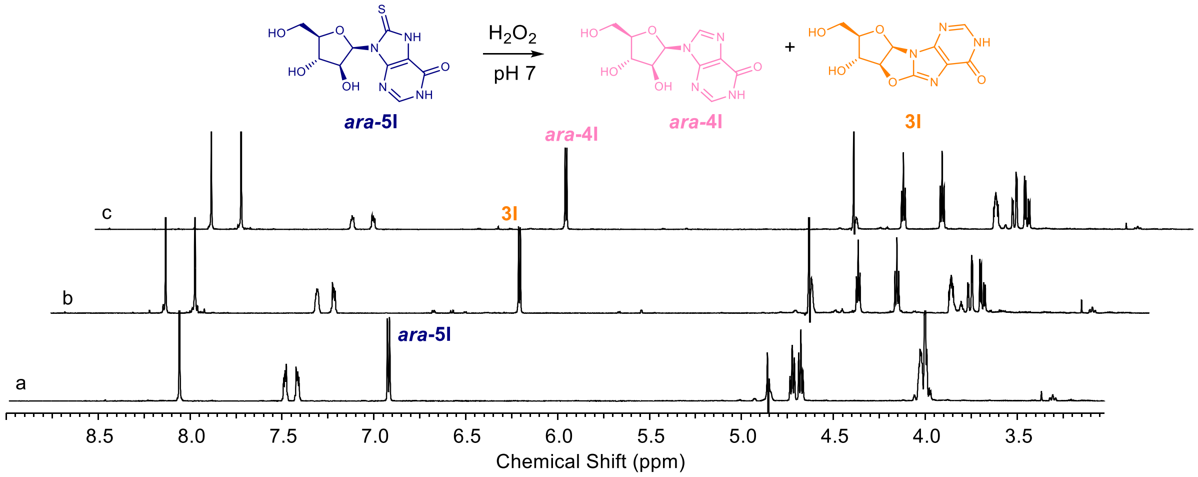

Supplementary Figure 19 | ^1H NMR (600 MHz, {H_2O/D_2O, 9:1}, noesygppr1d, 3.0–9.0 ppm) spectra showing the reaction of 8-mercapto-arabino-inosine ara-5I (50.0 mM) and potassium hydrogen phthalate 13 (10.0 mM; internal NMR standard) with H_2O_2 (150 mM) at pH 7 and room temperature. a. *Spectrum showing the reaction starting material.* ***b.*** *Spectrum showing the reaction products after 1 h then being adjusted to pH 7 and incubated for 1 h further at room temperature.* ***c.*** *Reaction products (spectrum b) after spiking with arabino-inosine* ***ara-4I****.*

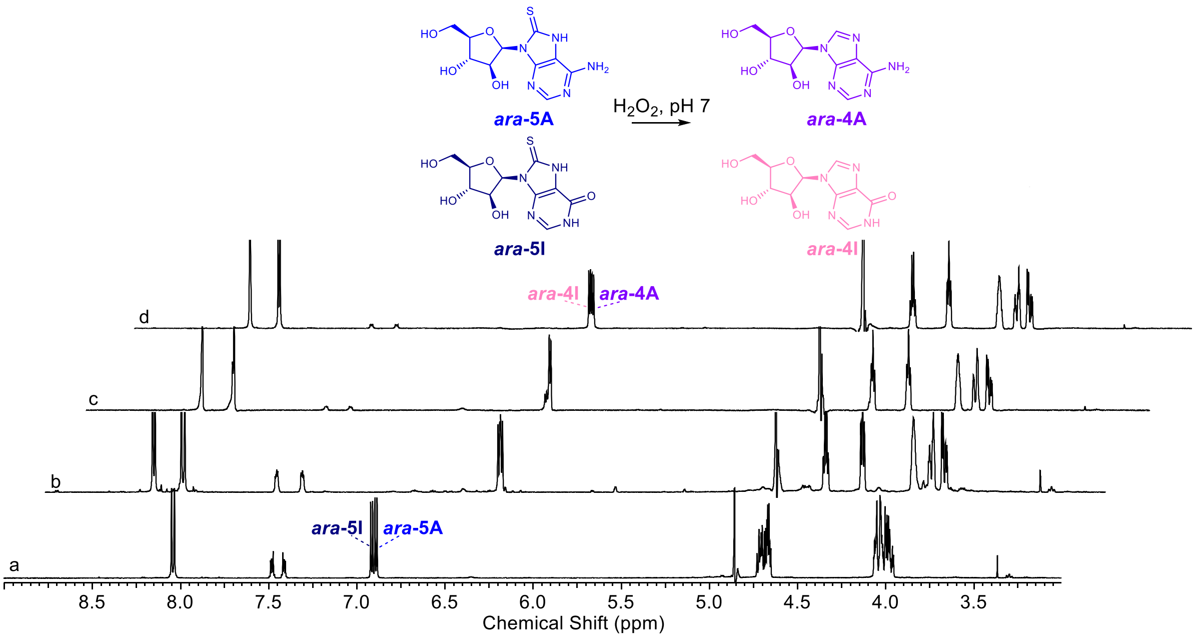

Supplementary Figure 20 | ^{1}H NMR (600 MHz, {H_2O/D_2O, 9:1}, noesygppr1d, 3.0–9.0 ppm) spectra showing the simultaneous reaction of 8-mercapto-arabino-adenosine ara-5A (25.0 mM), 8-mercapto-arabino-inosine ara-5I (25.0 mM) and potassium hydrogen phthalate 13 (10.0 mM; internal NMR standard) with H_2O_2 (150 mM) at pH 7 and room temperature. a. *Spectrum showing the reaction starting materials.* ***b.*** *Spectrum showing the reaction products after 1 h then being adjusted to pH 7 and incubated for 1 h further at room temperature.* ***c.*** *Spectrum showing the reaction products (spectrum b) after spiking with arabino-adenosine* ***ara-4A****.* ***d.*** *Spectrum showing the reaction products (spectrum c) after spiking with arabino-inosine* ***ara-4I****.*

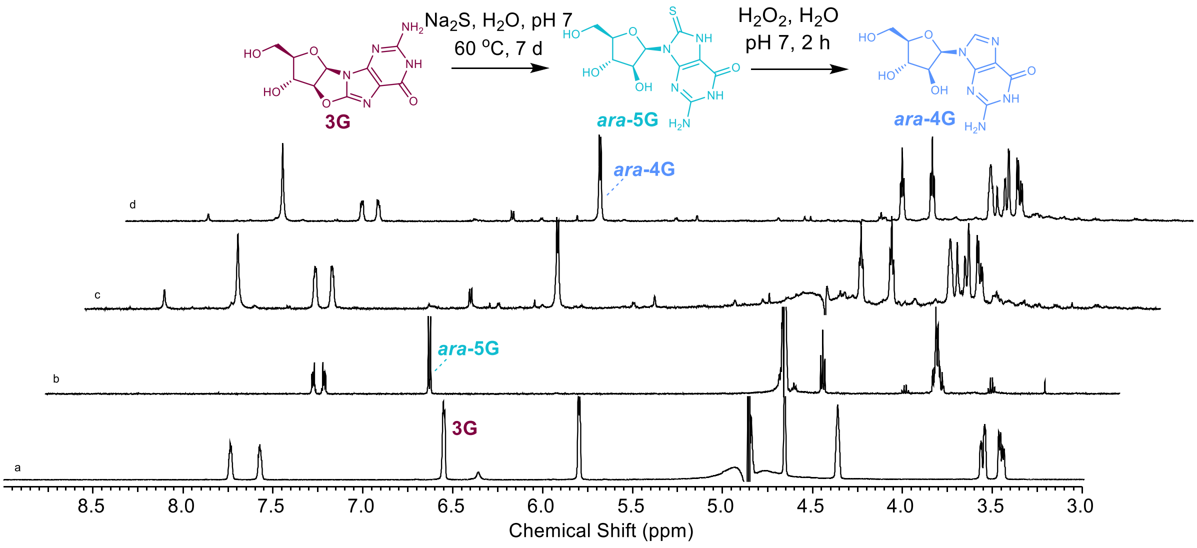

Supplementary Figure 21 | ^{1}H NMR (600 MHz, {H_2O/D_2O 9:1}, noesygppr1d, 3.0–9.0 ppm) spectra showing the sequential reaction of 2′,8-anhydroguanosine 3G (35.7 mM), potassium hydrogen phthalate 13 (7.14 mM; internal NMR standard), and Na_2S (714 mM) at pH 7 and 60 °C for 7 d followed by reaction with H_2O_2 (30% w/w, 39.0 µL, 375 µmol) at pH 7 and room temperature for 2 h. a. *Spectrum showing the reaction starting material.* ***b.*** *Spectrum showing the thiolysis products after 7 d.* ***c.*** *Spectrum showing the thiolysis products following reaction with H_2O_2 for 2 h.* ***d.*** *Spectrum showing the oxidation products (spectrum c) after spiking arabino-guanosine* ***ara-4G****.*

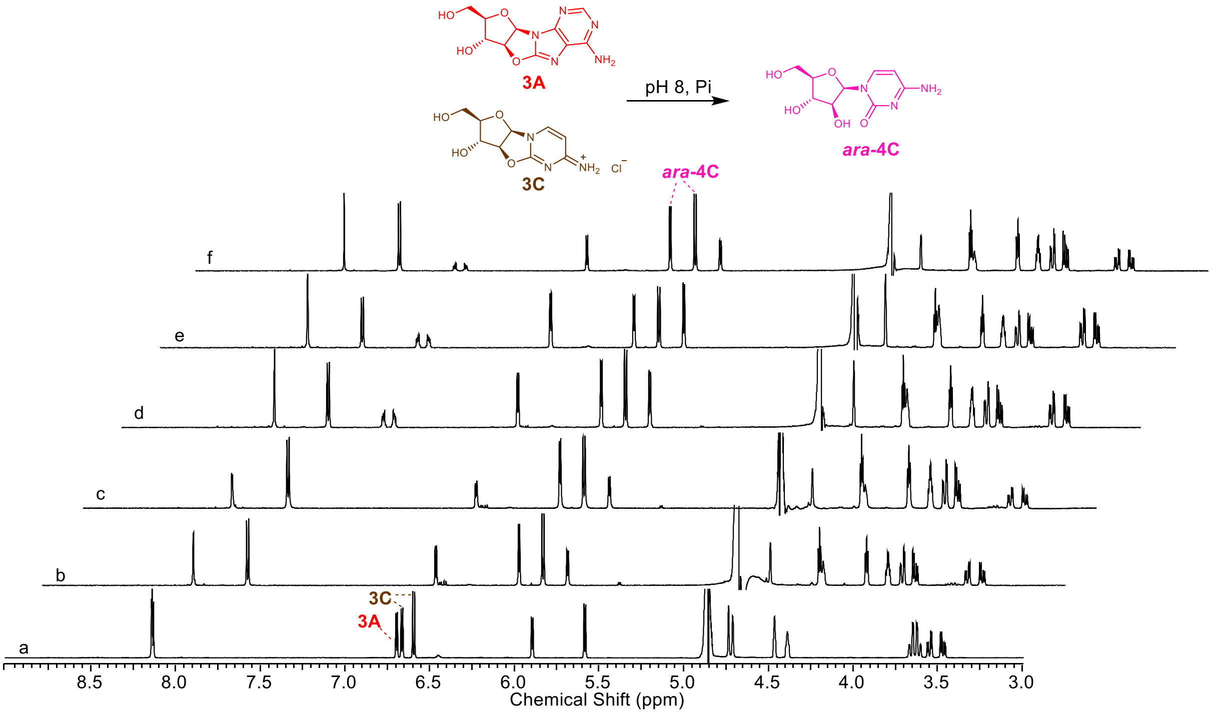

Supplementary Figure 22 | ^{1}H NMR (600 MHz, {H_2O/D_2O 9:1}, noesygppr1d, 1.0–9.0 ppm) spectra showing the reaction of 2′,8-anhydroadenosine 3A (12.5 mM) and arabino-2′,2-anhydrocytidine 3C (12.5 mM) in 0.5 M phosphate buffer (P_i) at pH 8. a. *Spectrum showing the reaction starting materials.* ***b.*** *Spectrum showing the reaction products after 6 h.* ***c.*** *Spectrum showing the reaction products after 24 h.* ***d.*** *Spectrum showing the reaction products after spiking with potassium hydrogen phthalate* ***13*** *(2.5 mM; internal NMR standard).* ***d.*** *Spectrum showing the reaction products (spectrum c) after spiking with 2′,8-anhydroadenosine* ***3A****;* ***e.*** *Spectrum showing the reaction products (spectrum d) after spiking arabino-cytidine* ***ara-4C****.*

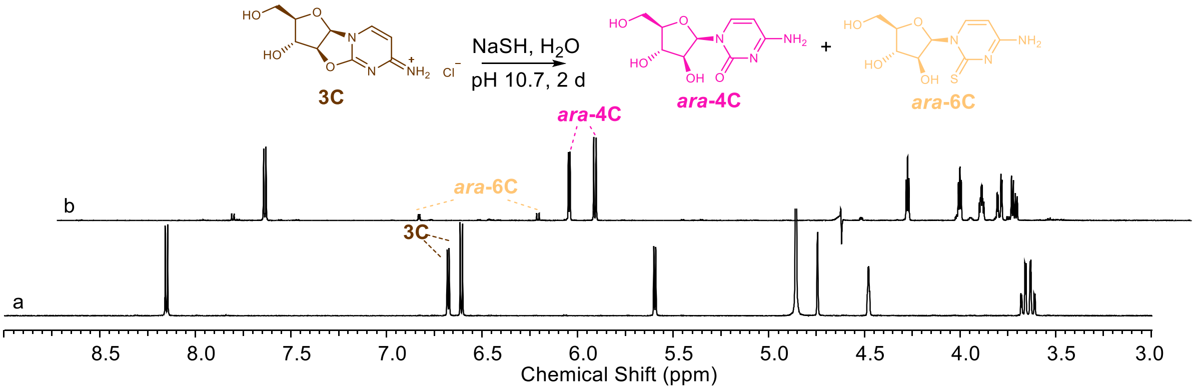

Supplementary Figure 23 | ^{1}H NMR spectra showing the reaction of 2′,2-anhydrocytidine 3C (31.3 mM) with NaHS (325 mM) at pH 10.7 and room temperature. a. *^{1}H NMR (600 MHz, {D_2O}, 3.5–8.5 ppm) spectrum showing the reaction starting material.* ***b.*** *^{1}H NMR (600 MHz, {H_2O/D_2O 9:1}, noesygppr1d 3.5–8.5 ppm) spectrum showing the reaction products after 2 d.*

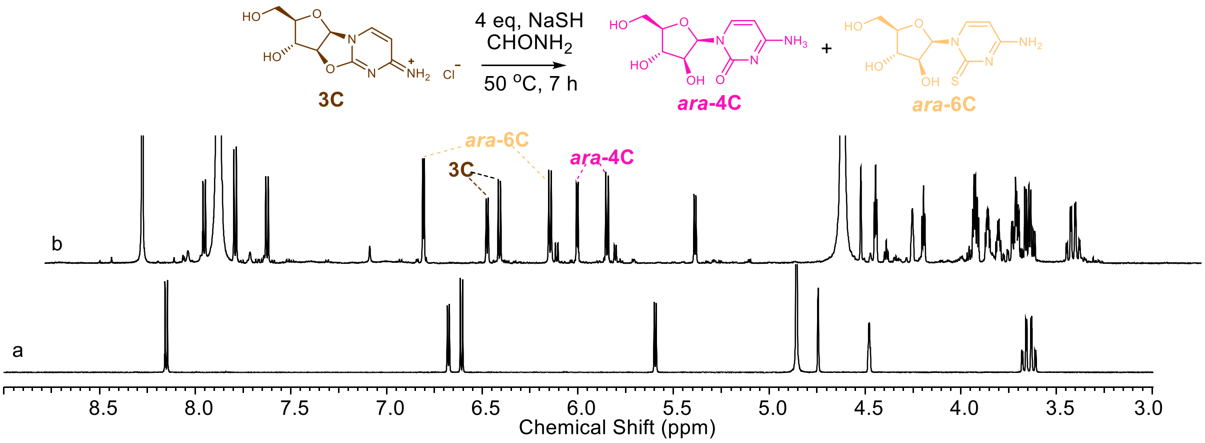

Supplementary Figure 24 | ^{1}H NMR (600 MHz, {D_2O}, 3.0-9.0 ppm) spectra showing the reaction of arabino-2′,2-anhydrocytidine 3C (38.0 mM) with NaSH (153 mM) in formamide at 50 ℃. a. *Spectrum showing the starting materials.* ***b.*** *Spectrum showing the thiolysis products after 7 h.*

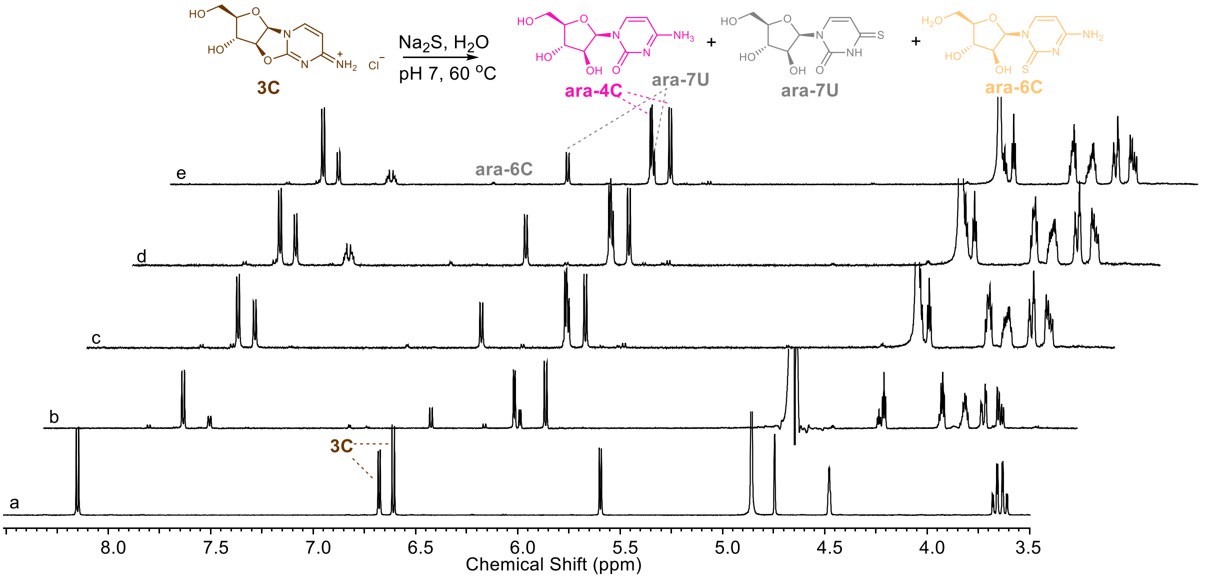

Supplementary Figure 25 | ^{1}H NMR spectra showing the reaction of 2′,2-anhydrocytidine 3C (27.8 mM) with Na_2S (556 mM) at pH 7 and 60 °C. a. *^{1}H NMR (600 MHz, {D_2O}, 3.5–8.5 ppm) spectrum showing the reaction starting material.* ***b.*** *^{1}H NMR (600 MHz, {H_2O/D_2O 9:1}, noesygppr1d 3.5–8.5 ppm) spectrum showing the reaction products after 2 d.* ***c.*** *^{1}H NMR (600 MHz, {D_2O}, noesygppr1d, 3.5–8.5 ppm) spectrum showing the reaction products after 7 d.* ***d.*** *Spectrum showing the reaction products (spectrum c) after spiking with potassium hydrogen phthalate* ***13*** *(5.00 µmol; internal NMR standard).* ***e.*** *Spectrum showing the reaction products (spectrum d) after spiking with arabino-cytidine* ***ara-4C****.*

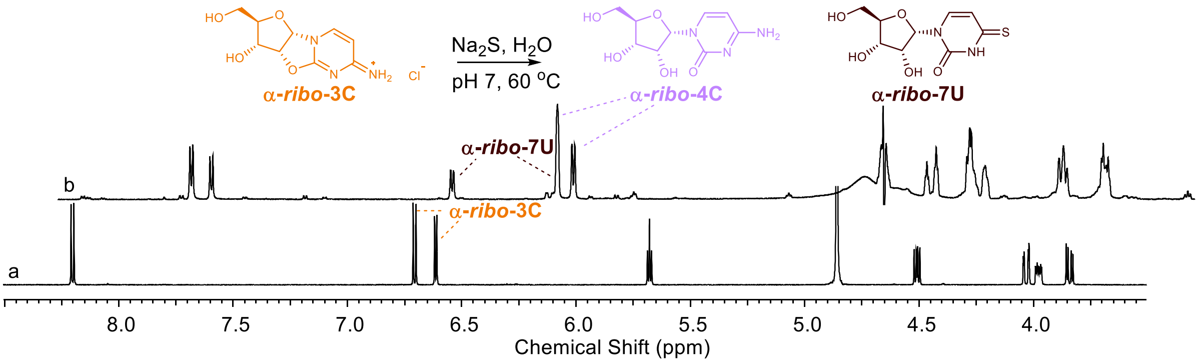

Supplementary Figure 26 | ^{1}H NMR spectra showing the reaction of ribo-2′,2-anhydrocytidine α-ribo-3C (27.8 mM) with Na_2S (556 mM) at pH 7 and 60 °C for 7 d. a. *^{1}H NMR (600 MHz, {D_2O}, 3.5–8.5 ppm) spectrum showing the reaction starting material.* ***b.*** *^{1}H NMR (600 MHz, {H_2O/D_2O 9:1}, noesygppr1d, 3.5–8.5 ppm) spectrum showing the reaction products.*

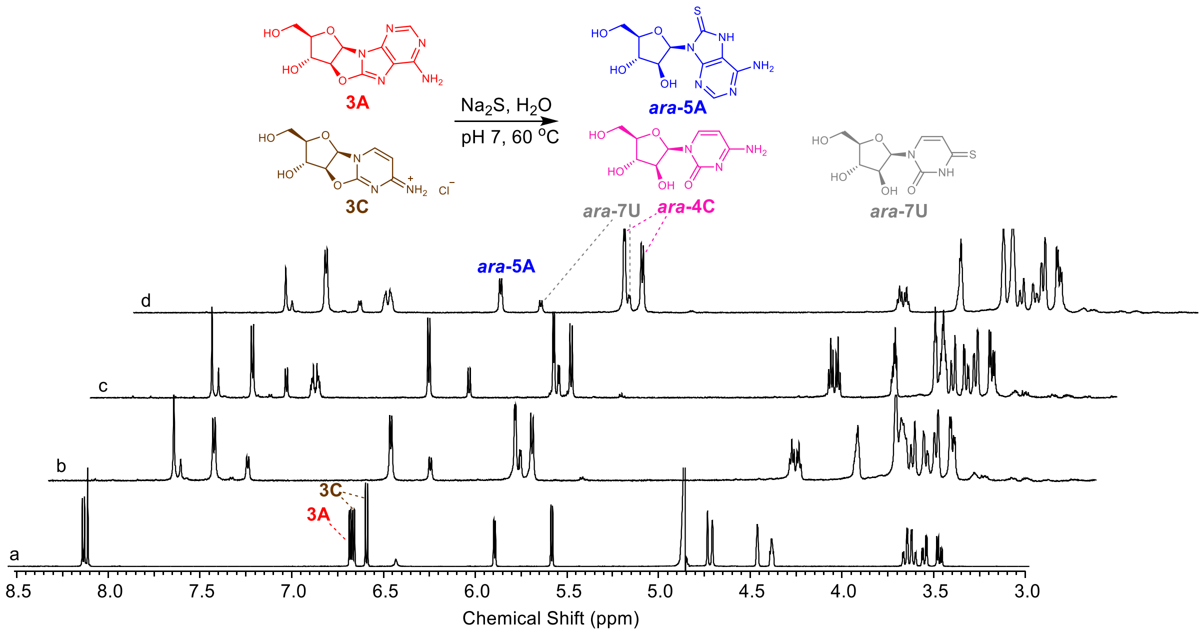

Supplementary Figure 27 | ^{1}H NMR spectra showing the reaction of arabino-2′,2-anhydrocytidine 3C (31.3 mM) and 2′,8-anhydroadenosine 2A (31.3 mM) with Na_2S (1.25 M) at pH 7 and 60 °C. a. *^{1}H NMR (600 MHz, {H_2O/D_2O 9:1}, noesygppr1d, 3.1–8.5 ppm) spectrum showing the reaction starting materials.* ***b.*** *^{1}H NMR (600 MHz, {D_2O}, noesygppr1d, 3.1–8.5 ppm) spectrum showing the reaction products after 7 d.* ***c.*** *Spectrum showing the reaction products (spectrum b) after spiking with potassium hydrogen phthalate* ***13*** *(5.00 µmol; internal NMR standard).* ***d.*** *Spectrum showing the reaction products (spectrum c) after spiking with arabino-cytidine* ***ara-4C****.*

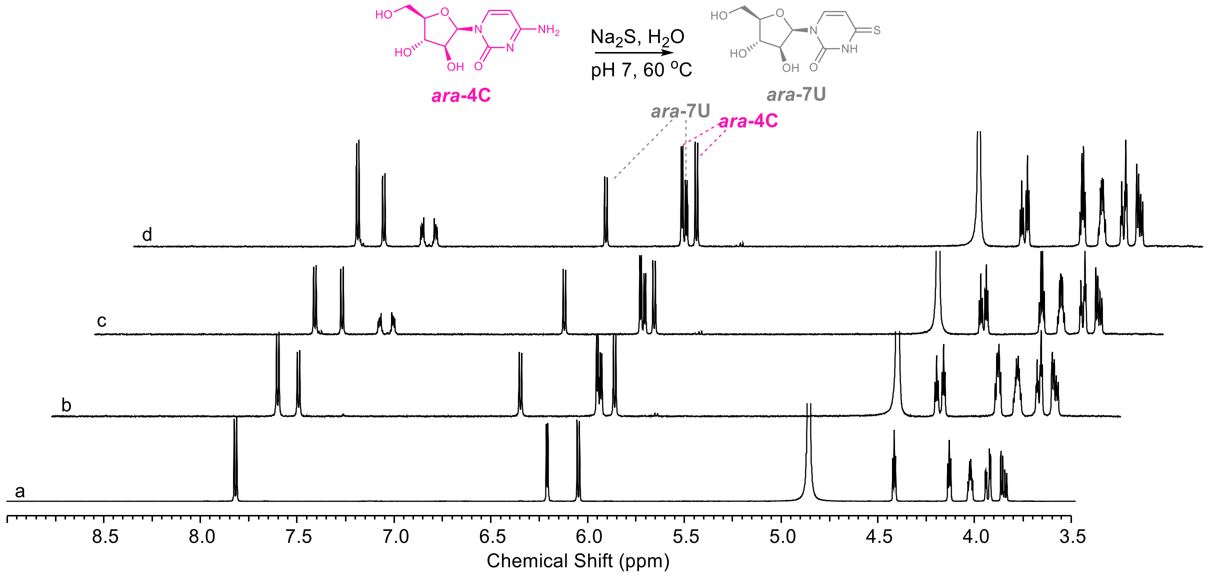

Supplementary Figure 28 | ^{1}H NMR (600 MHz, {D_2O}, 3.5–9.0 ppm) spectra showing the reaction of arabino-cytidine ara-4C (55.8 mM) with Na_2S (1.12 M) at pH 7 and 60 °C. a. *Spectrum of the reaction starting material.* ***b.*** *Spectrum showing reaction products after 7 d.* ***c.*** *Spectrum showing the reaction products (spectrum b) after spiking with potassium hydrogen phthalate* ***13*** *(5.00 µmol; internal NMR standard).* ***d.*** *Spectrum showing the reaction products (spectrum c) after spiking with arabino-cytidine* ***ara-4C****.*

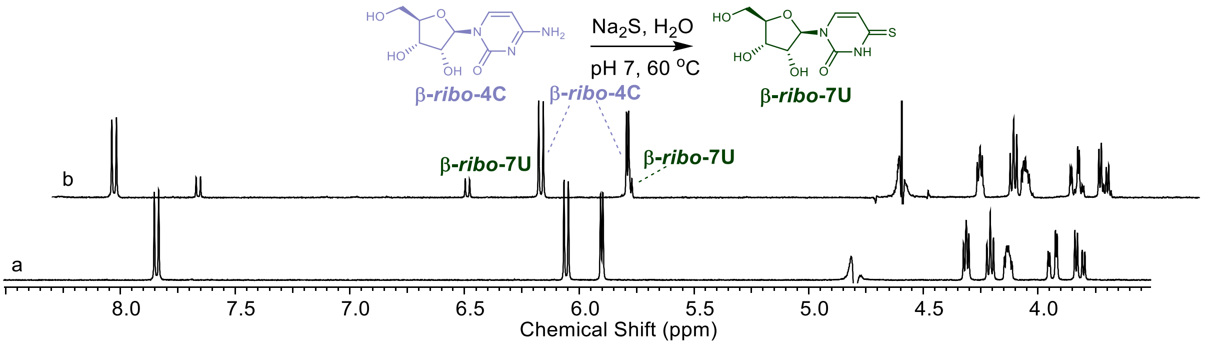

Supplementary Figure 29 | ^{1}H NMR (600 MHz, {D$_2$O}, noesygppr1d, 3.5–8.5 ppm) spectra showing the reaction of β-ribo-cytidine β-ribo-4C (42 mM) with Na$_2$S (420 mM) at pH 7 and 60 °C. a. *Spectrum of the reaction starting materials.* ***b.*** *Spectrum showing reaction products after 7 d.*

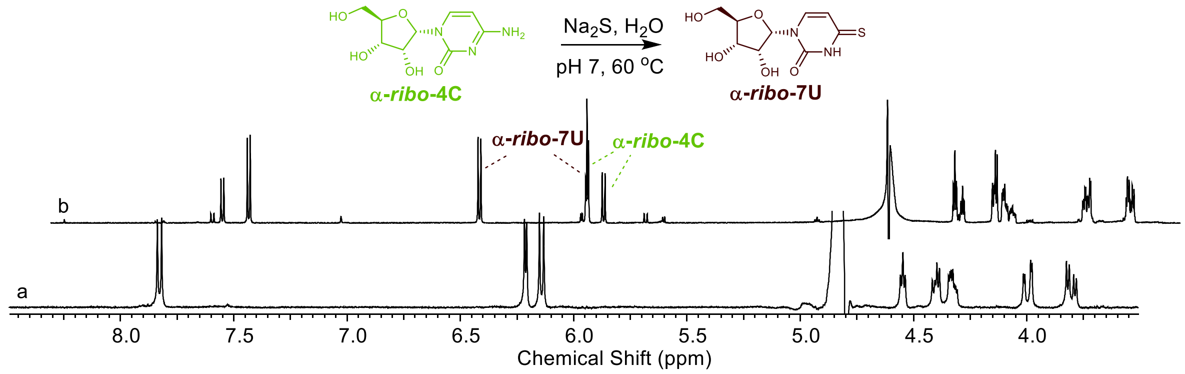

Supplementary Figure 30 | ^{1}H NMR (600 MHz, {D$_2$O}, noesygppr1d, 3.5–8.5 ppm) spectra showing the reaction of α-ribo-cytidine α-ribo-4C (42 mM) with Na$_2$S (420 mM) at pH 7 and 60 °C. a. *Spectrum of the reaction starting material.* ***b.*** *Spectrum showing reaction products after 7 d.*

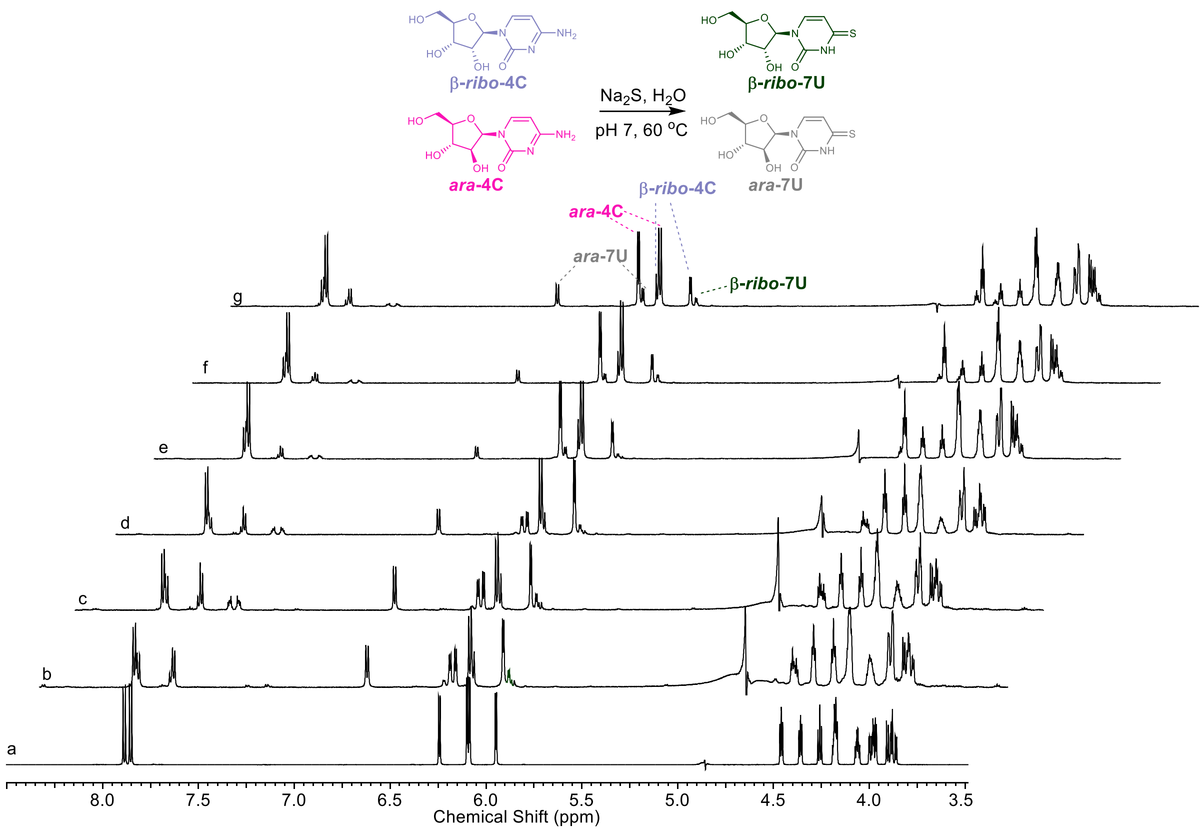

***Supplementary Figure 31** | **^{1}H NMR (600 MHz, {H_2O/D_2O 9:1}, noesygppr1d, 3.5–8.5 ppm) spectra showing the simultaneous reaction of ribo-cytidine β-ribo-4C (71.4 mM) and arabino-cytidine ara-4C (71.4 mM) with Na_2S (1.43 M) at pH 7 and 60 °C. a.** Spectrum of the reaction starting material. **b.** Spectrum showing reaction products after 7 d. **c.** Spectrum showing the reaction products (spectrum b) after spiking with potassium hydrogen phthalate **13** (5.00 µmol; internal NMR standard). **d.** Spectrum showing the reaction products (spectrum c) after spiking with ribo-cytidine **β-ribo-4C**. **e.** Spectrum showing the reaction products (spectrum d) after spiking with arabino-cytidine **ara-4C**. **f.** Spectrum showing the reaction products (spectrum e) after spiking with 4-thio-ribo-cytidine **β-ribo-7U**. **g.** Spectrum showing the reaction products (spectrum f) after spiking with 4-thio-arabino-cytidine **ara-7U**.*

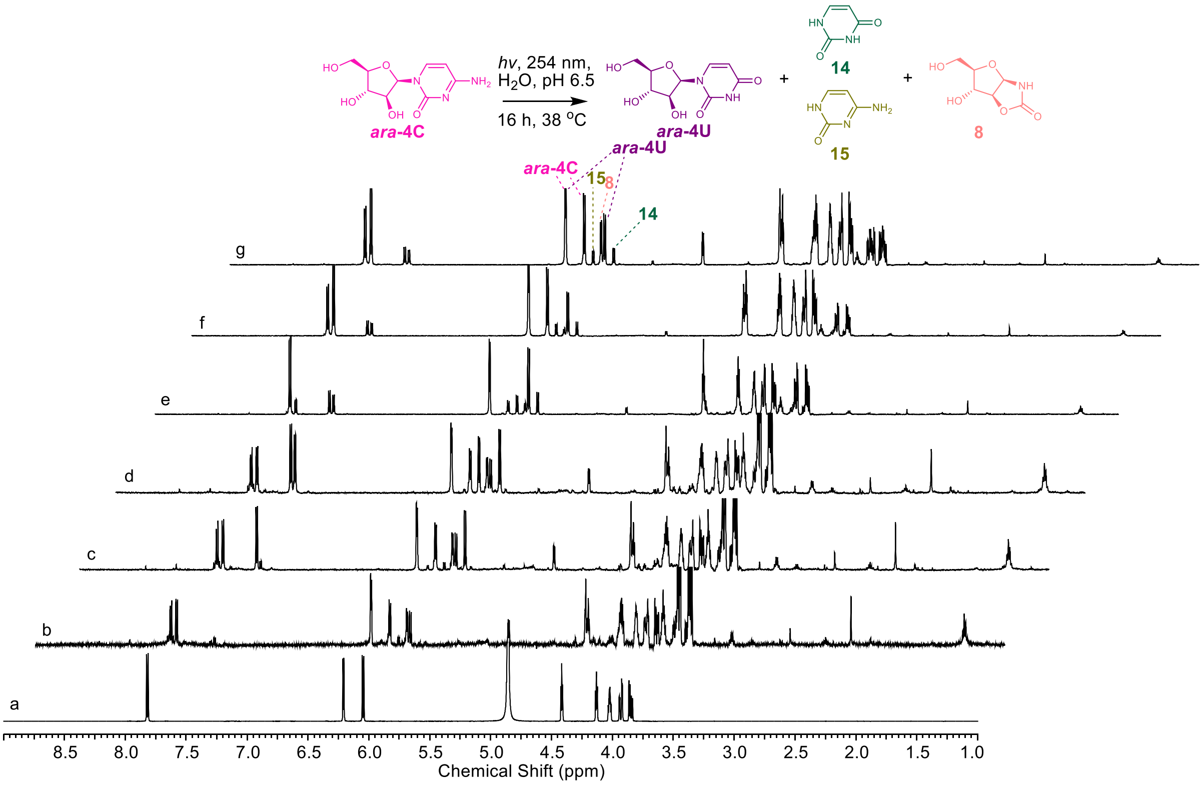

Supplementary Figure 32 | ^{1}H NMR spectra showing the irradiation (254 nm) at pH 6.5 and 38 °C of arabino-cytidine ara-4C (2 mM) for 16 h. a. *^{1}H NMR (600 MHz, {D₂O}, noesygppr1d, 1.0–9.0 ppm) spectrum showing the starting material.* ***b.*** *^{1}H NMR (600 MHz, {D₂O}, noesygppr1d, 1.0–9.0 ppm) spectrum showing the irradiation products after 16 h and subsequent photo-hydrate relaxation (90 °C) for 16 h.* ***c.*** *Spectrum showing the irradiation products (spectrum b) after spiking with uracil* ***14****.* ***d.*** *Spectrum showing the irradiation products (spectrum c) after spiking with cytosine* ***15****.* ***e.*** *Spectrum showing the irradiation products (spectrum d) after spiking with arabino-uridine* ***ara-4U****.* ***f.*** *Spectrum showing the irradiation products (spectrum e) after spiking with arabino-cytidine* ***ara-4C****.* ***g.*** *Spectrum showing the irradiation products (spectrum f) after spiking with arabino-oxazolidinone* ***8****.*

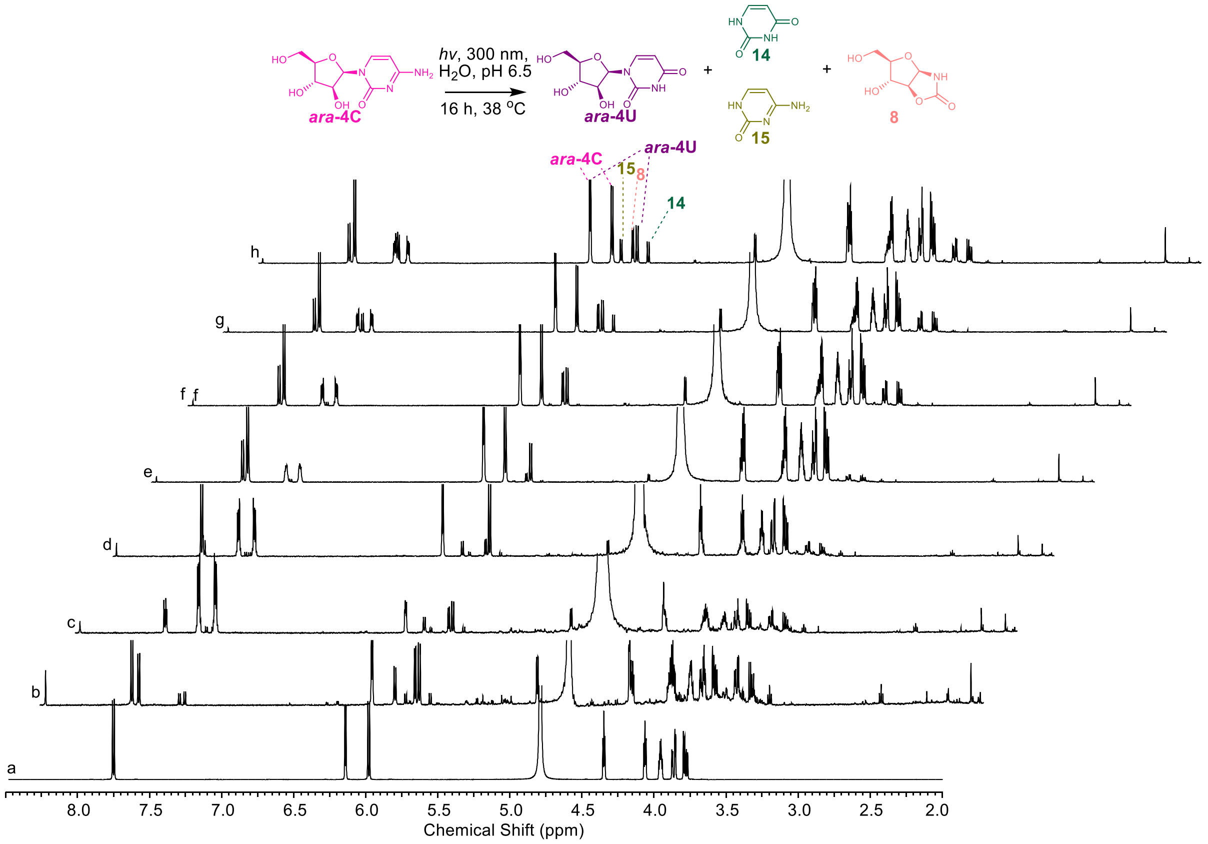

Supplementary Figure 33 | ^{1}H NMR (600 MHz, {D_2O}, 2.0–8.5 ppm) spectra showing the irradiation (300 nm) at pH 6.5 and 38 °C of arabino-cytidine ara-4C (2 mM) for 16 h. a. *Spectrum showing the starting materials.* ***b.*** *Spectrum showing the irradiation products after 16 h.* ***c.*** *Spectrum showing the irradiation products (spectrum b) after spiking with potassium hydrogen phthalate* ***13*** *(5 µmol; internal standard).* ***d.*** *Spectrum showing the irradiation products (spectrum c) after spiking with arabino-uridine* ***ara-4U****.* ***e.*** *Spectrum showing the irradiation products (spectrum d) after spiking with arabino-cytidine* ***ara-4C****.* ***f.*** *Spectrum showing the irradiation products (spectrum e) after spiking with arabino-oxazolidinone* ***8****.* ***g.*** *Spectrum showing the irradiation products (spectrum f) after spiking with cytosine* ***15****.* ***h.*** *Spectrum showing the irradiation products (spectrum g) after spiking with uracil* ***14****.*

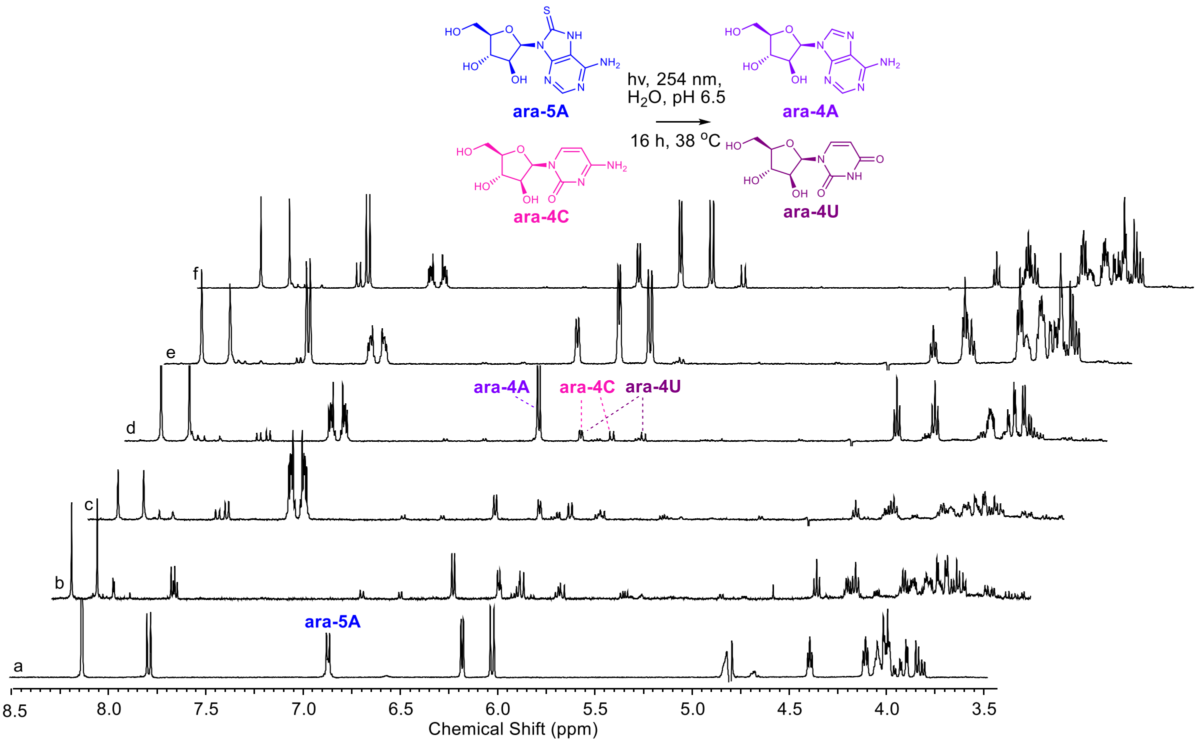

Supplementary Figure 34 | ^{1}H NMR spectra showing the irradiation (254 nm) at pH 6.5 and 38 °C of 8-mercapto-arabino-adenosine ara-5A (1 mM) and arabino-cytidine ara-4C (1 mM). a. *^{1}H NMR (600 MHz, {H_2O/D_2O 9:1}, noesygppr1d, 3.5–8.5 ppm) spectrum showing the starting materials.* ***b.*** *^{1}H NMR (600 MHz, {D_2O}, noesygppr1d, 3.5–8.5 ppm) spectrum showing the irradiation products after 16 h and subsequent photo-hydrate relaxation (90 °C) for 16 h.* ***c.*** *Spectrum showing the irradiation products (spectrum b) after spiking with potassium hydrogen phthalate* ***13*** *(5.0 µmol; internal NMR standard).* ***d.*** *Spectrum showing the irradiation products (spectrum c) after spiking with arabino-adenosine* ***ara-4A****.* ***e.*** *Spectrum showing the irradiation products (spectrum d) after spiking with arabino-cytidine* ***ara-4C****.* ***f.*** *Spectrum showing the irradiation products (spectrum e) after spiking with arabino-uridine* ***ara-4U****.*

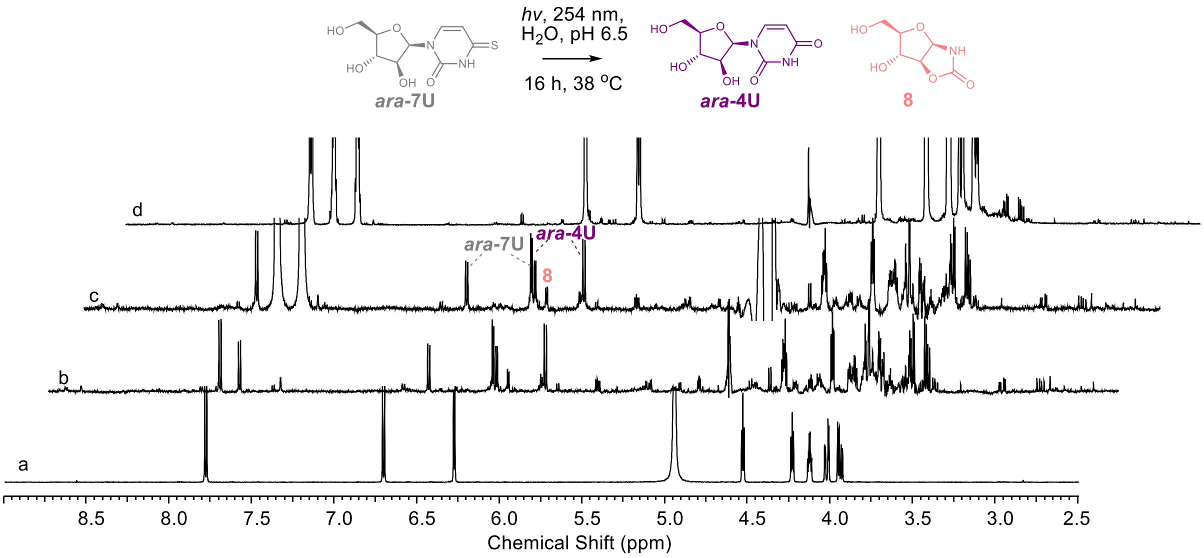

Supplementary Figure 35 | ^{1}H NMR spectra showing the irradiation (254 nm) at pH 6.5 and 38 °C of 4-thio-arabino-uridine ara-7U (1.52 mM) for 16 h. a. *^{1}H NMR (600 MHz, {D_2O}, 2.5–9.0 ppm) spectrum showing the starting material.* ***b.*** *^{1}H NMR (600 MHz, {D_2O}, noesygppr1d, 2.5–9.0 ppm) spectrum showing the irradiation products after 16 h and subsequent photo-hydrate relaxation (90 °C) for 16 h.* ***c.*** *Spectrum showing the irradiation products after spiking with potassium hydrogen phthalate* ***13*** *(5.00 µmol; internal NMR standard).* ***d.*** *Spectrum showing the irradiation products (spectrum c) after spiking with arabino-uridine* ***ara-4U****.*

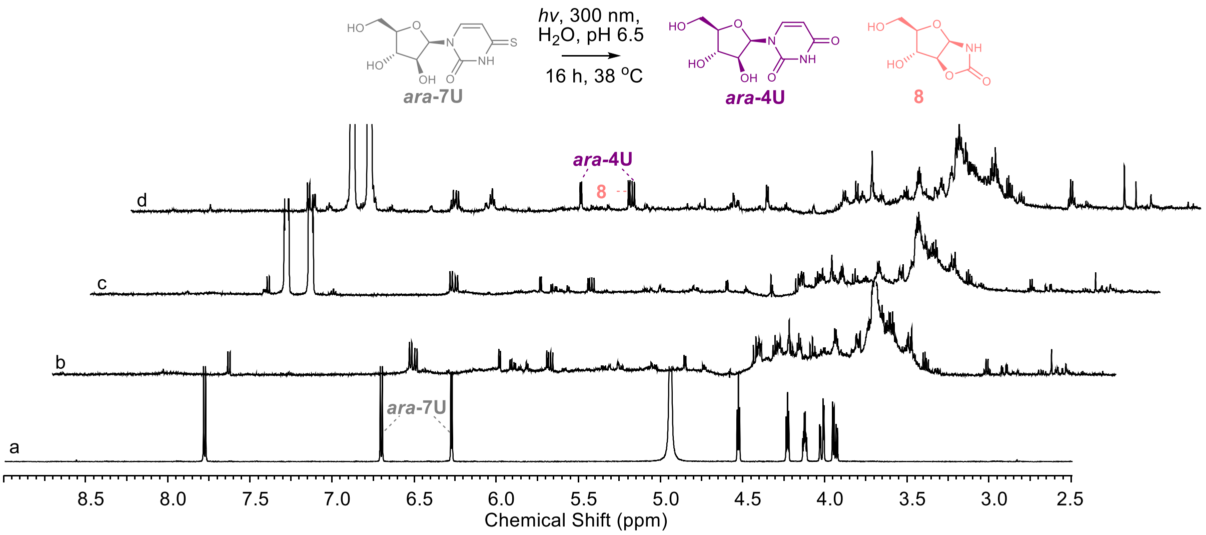

Supplementary Figure 36 | ^{1}H NMR spectra showing the irradiation (300 nm) at pH 6.5 and 38 °C of 4-thio-arabino-uridine ara-7U (2.00 mM) for 16 h. a. *^{1}H NMR (600 MHz, {D_2O}, 2.5–9.0 ppm) spectrum showing the starting material.* ***b.*** *^{1}H NMR (600 MHz, {D_2O}, noesygppr1d, 2.5–9.0 ppm) spectrum showing the irradiation products after 16 h.* ***c.*** *Spectrum showing the irradiation products after spiking with potassium hydrogen phthalate* ***13*** *(5.00 µmol; internal NMR standard).* ***d.*** *Spectrum showing the irradiation products (spectrum c) after subsequent photo-hydrate relaxation (90 °C) for 16 h.*

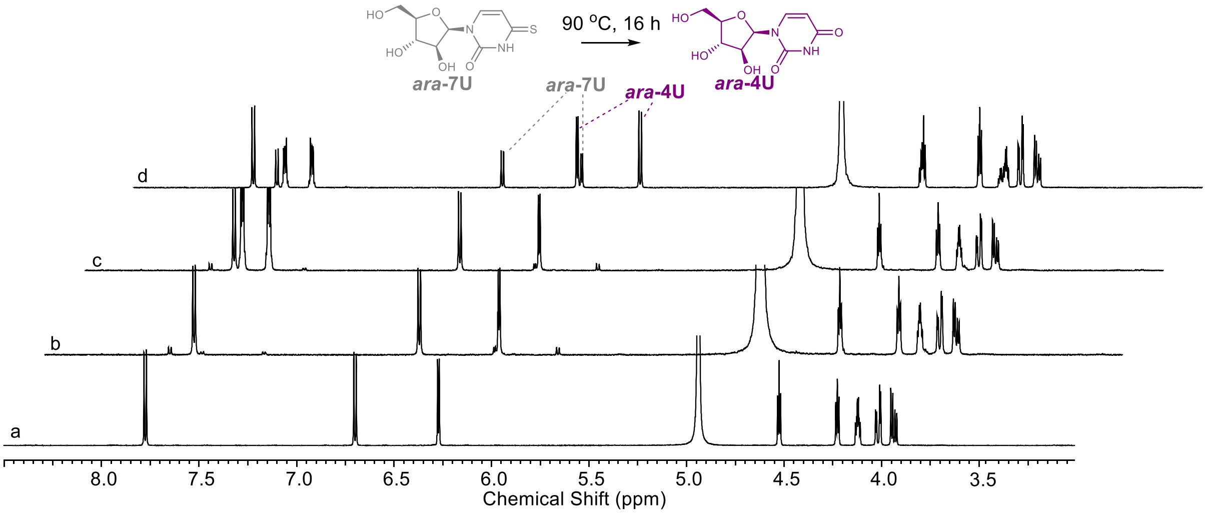

Supplementary Figure 37 | ^{1}H NMR (600 MHz, {D_2O}, 3.0–8.5 ppm) spectra showing products of heating 4-thio-arabino-uridine ara-7U (54.5 µM) at 90 ℃ for 16 h. a. *Spectrum showing reaction starting material.* ***b.*** *Spectrum showing reaction products after heating at 90 ℃ for 16 h.* ***c.*** *Spectrum showing the reaction products (spectrum b) after spiking with potassium hydrogen phthalate* ***13*** *(5.00 µmol; internal NMR standard).* ***d.*** *Spectrum showing the reaction products (spectrum c) after spiking with arabino-uridine* ***ara-4U****.*

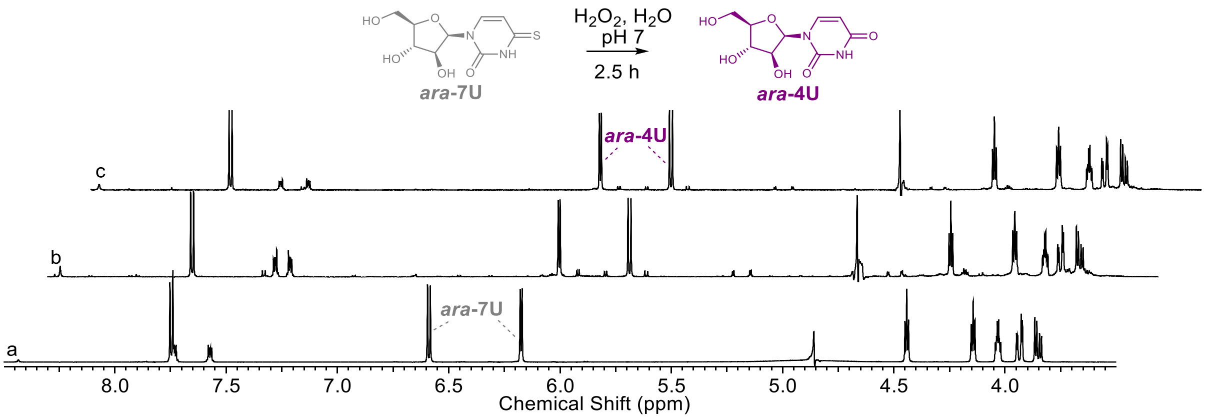

Supplementary Figure 38 | ^{1}H NMR (600 MHz, {H_2O/D_2O, 9:1}, noesygppr1d, 3.5–8.5 ppm) spectra showing the reaction of 4-thio-arabino-uridine ara-7U (50.0 mM) and potassium hydrogen phthalate 13 (10.0 mM; internal NMR standard) with H_2O_2 (150 mM) at pH 7 and room temperature. a. *Spectrum showing the reaction starting material.* ***b.*** *Spectrum showing reaction products after 1 h then being adjusted to pH 7 and incubated for 1 h further at room temperature.* ***c.*** *Spectrum showing the reaction products (spectrum b) after spiking with arabino-uridine* ***ara-4U****.*

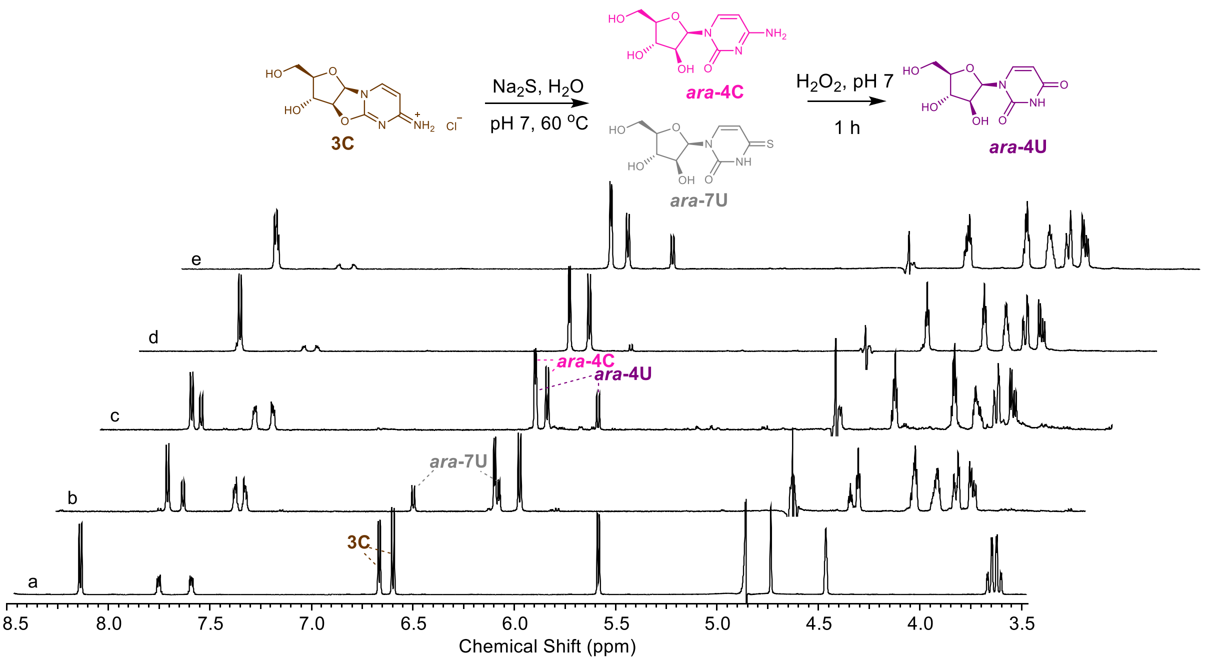

Supplementary Figure 39 | ^{1}H NMR (600 MHz, {H_2O/D_2O, 9:1}, noesygppr1d, 3.5-8.5 ppm) spectra showing the sequential reaction of arabino-2′,2-anhydrocytidine 3C (35.7 mM), potassium hydrogen phthalate 13 (7.14 mM, internal NMR standard), and Na_2S (714 mM) at pH 7 and 60 °C for 7 d followed by reaction with H_2O_2 (30% w/w, 39.0 µL, 375 µmol) at pH 7 and room temperature for 1 h. a. *Spectrum showing reaction starting material.* ***b.*** *Spectrum showing the thiolysis products after 7 d.* ***c.*** *Spectrum showing the thiolysis products following reaction with H_2O_2 (30% w/w, 39.0 µL, 375 µmol) for 1 h.* ***d.*** *Spectrum showing the oxidation products (spectrum c) after spiking with arabino-cytidine* ***ara-4C****;* ***e.*** *Spectrum showing the oxidation products (spectrum d) after spiking with arabino-uridine* ***ara-4U****.*

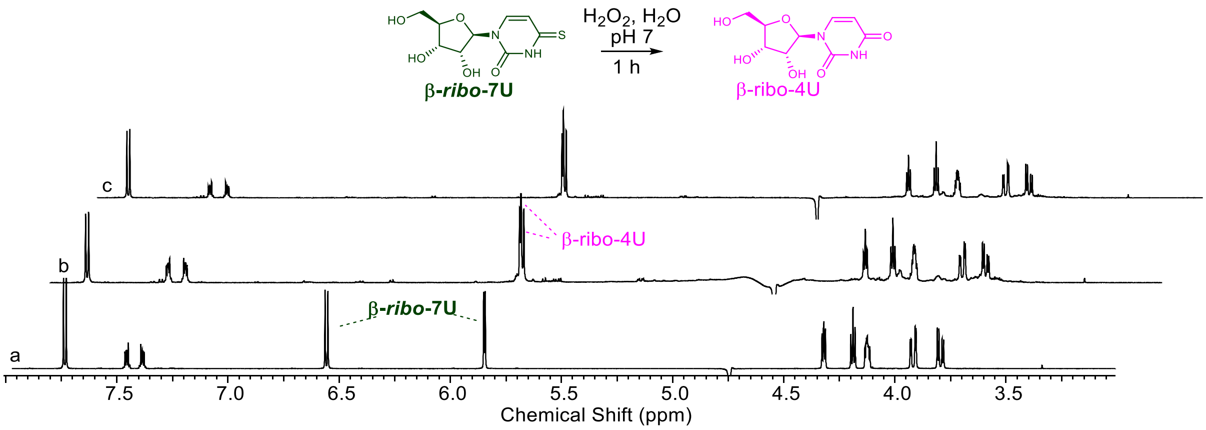

Supplementary Figure 40 | ^{1}H NMR (600 MHz, {H_2O/D_2O, 9:1}, noesygppr1d, 3.0–8.0 ppm) spectra showing the reaction of 4-thio-ribo-uridine β-ribo-7U (50.0 mM) and potassium hydrogen phthalate 13 (8.79 mM; internal NMR standard) with H_2O_2 (150 mM) at pH 7 and room temperature. a. *Spectrum showing the reaction starting material.* ***b.*** *Spectrum showing reaction products after 1 h then being adjusted to pH 7 and incubated for 1 h further at room temperature.* ***c.*** *Spectrum showing the reaction products (spectrum b) after spiking with ribo-uridine* ***β-ribo-4U****.*

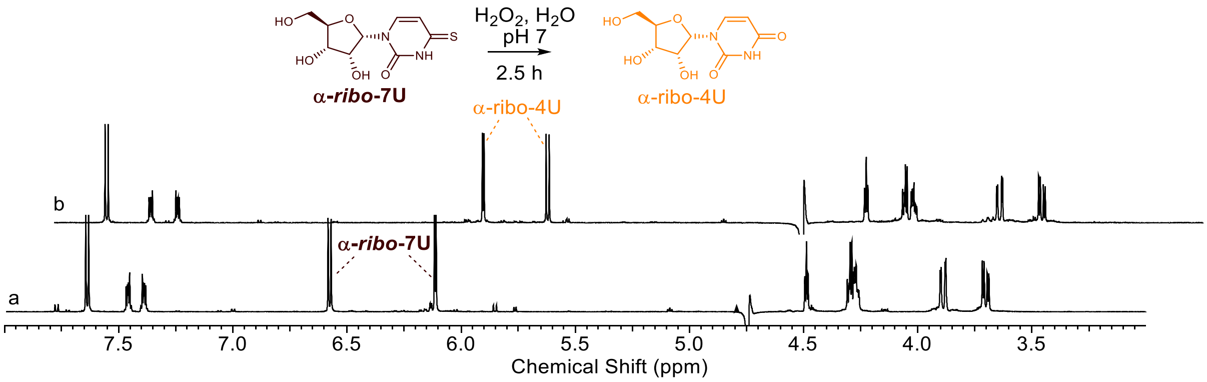

Supplementary Figure 41 | ^{1}H NMR (600 MHz, {H_2O/D_2O, 9:1}, noesygppr1d, 3.0–8.0 ppm) spectra showing the reaction of 4-thio-α-ribo-uridine α-ribo-7U (50.0 mM) and potassium hydrogen phthalate 13 (6.77 mM; internal NMR standard) with H_2O_2 (150 mM) at pH 7 and room temperature. a. *Spectrum showing the reaction starting material.* ***b.*** *Spectrum showing reaction products after 1 h then being adjusted to pH 7 and incubated for 3 h further at room temperature.*

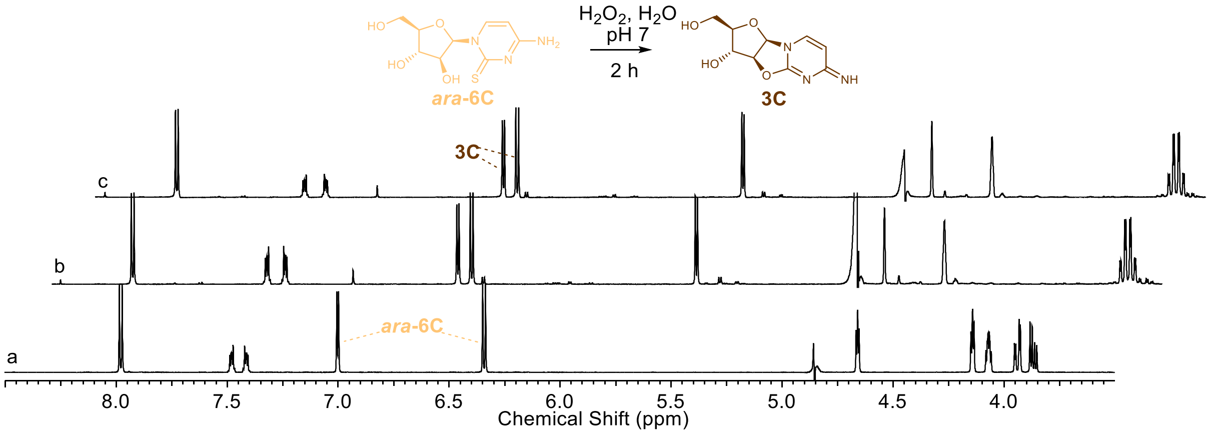

Supplementary Figure 42 | ^{1}H NMR (600 MHz, {H_2O/D_2O, 9:1}, noesygppr1d, 3.5–8.5 ppm) spectra showing the reaction of 2-thio-arabino-cytidine ara-6C (50.0 mM) and potassium hydrogen phthalate 13 (10.0 mM; internal NMR standard) with H_2O_2 (150 mM) at pH 7 and room temperature. a. *Spectrum showing the reaction starting materials.* ***b.*** *Spectrum showing reaction products after 1 h then being adjusted to pH 7 and incubated for 1 h further at room temperature.* ***c.*** *Spectrum showing the reaction products (spectrum b) after spiking with arabino-2',2-anhydrocytidine* ***3C****.*

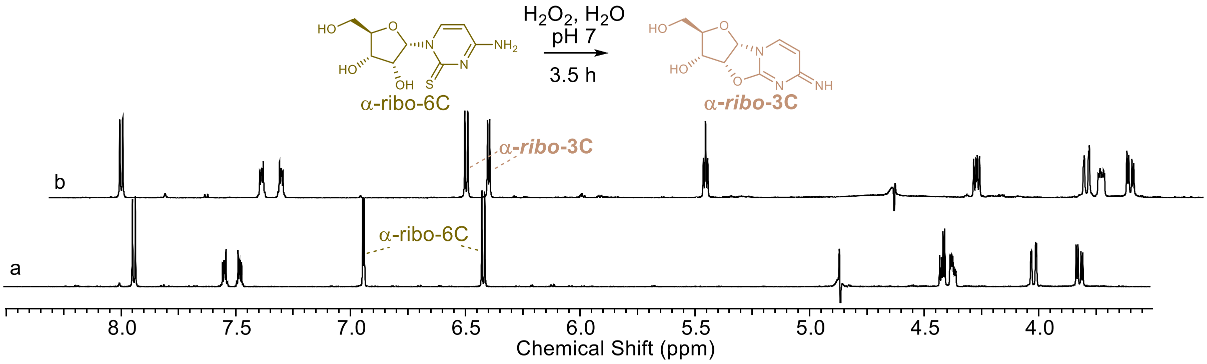

Supplementary Figure 43 | ^{1}H NMR (600 MHz, {H_2O/D_2O, 9:1}, noesygppr1d, 3.5–8.5 ppm) spectra showing the reaction of 2-thio-α-ribo-cytidine α-ribo-6C (50.0 mM) and potassium hydrogen phthalate 13 (10.0 mM; internal NMR standard) with H_2O_2 (150 mM) at pH 7 and room temperature. a. *Spectrum showing the reaction starting materials.* ***b.*** *Spectrum showing reaction products after 2.5 h then being adjusted to pH 7 and incubated for 1 h further at room temperature.*

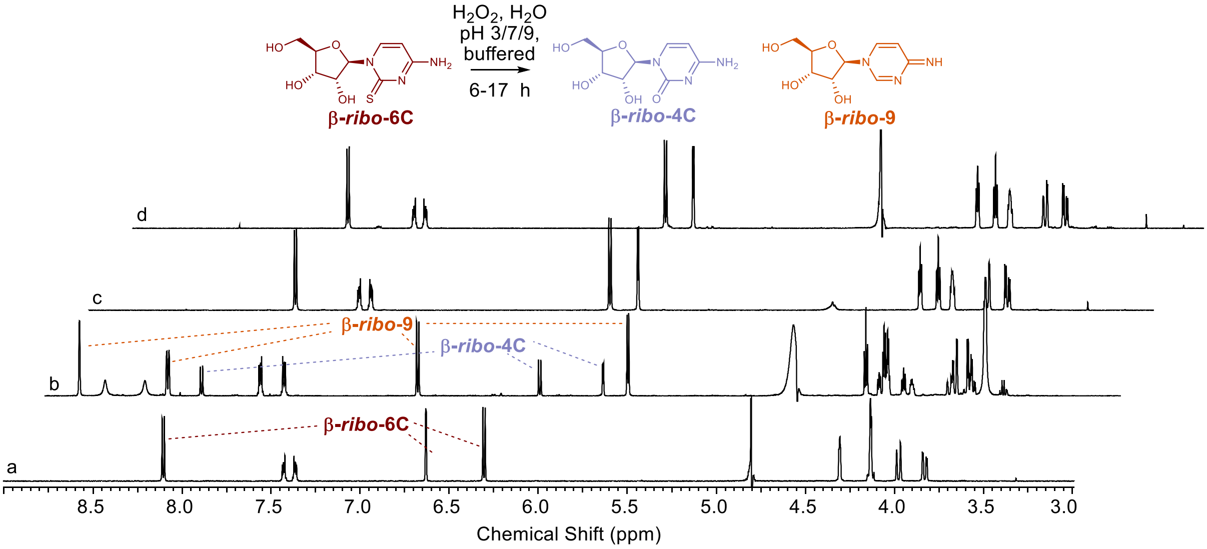

Supplementary Figure 44 | ^{1}H NMR (600 MHz, {H_2O/D_2O, 9:1}, noesygppr1d, 3.0–9.0 ppm) spectra showing the reaction of 2-thio-ribo-cytidine β-ribo-6C (38.4 mM) and potassium hydrogen phthalate 13 (10.0 mM; internal NMR standard) with H_2O_2 (116 or 232 mM) at room temperature whilst buffered at pH 3 by glycine (0.100 M) or at pH 7 and 9 by phosphate (0.100 M). a. *Spectrum showing the reaction starting material.* ***b.*** *Spectrum showing reaction products at pH 3 after 17 h.* ***c.*** *Spectrum showing reaction products at pH 7 after 7 h.* ***d.*** *Spectrum showing reaction products at pH 9 after 6 h.*

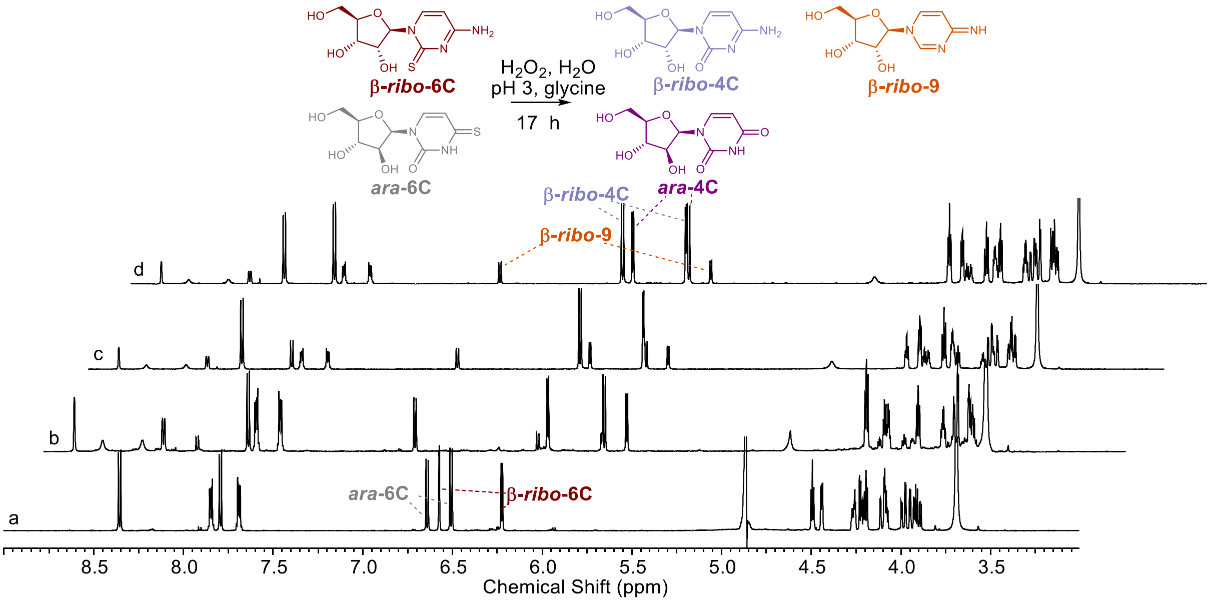

Supplementary Figure 45 | ^{1}H NMR (600 MHz, {H_2O/D_2O, 9:1}, noesygppr1d, 3.0–9.0 ppm) spectra showing the simultaneous reaction of 2-thio-ribo-cytidine β-ribo-6C (19.3 mM), 4-thio-arabino-uridine ara-7U (19.3 mM) and potassium hydrogen phthalate 13 (10.0 mM; internal NMR standard) with H_2O_2 (232 mM) at room temperature whilst buffered at pH 3 by glycine (0.100 M). a. *Spectrum showing the reaction starting material.* ***b.*** *Spectrum showing reaction products at pH 3 after 19 h.* ***c.*** *Spectrum showing the reaction products (spectrum b) after spiking with ribo-cytidine* ***β-ribo-4C****.* ***d.*** *Spectrum showing the reaction products (spectrum c) after spiking with arabino-uridine* ***ara-4U****.*

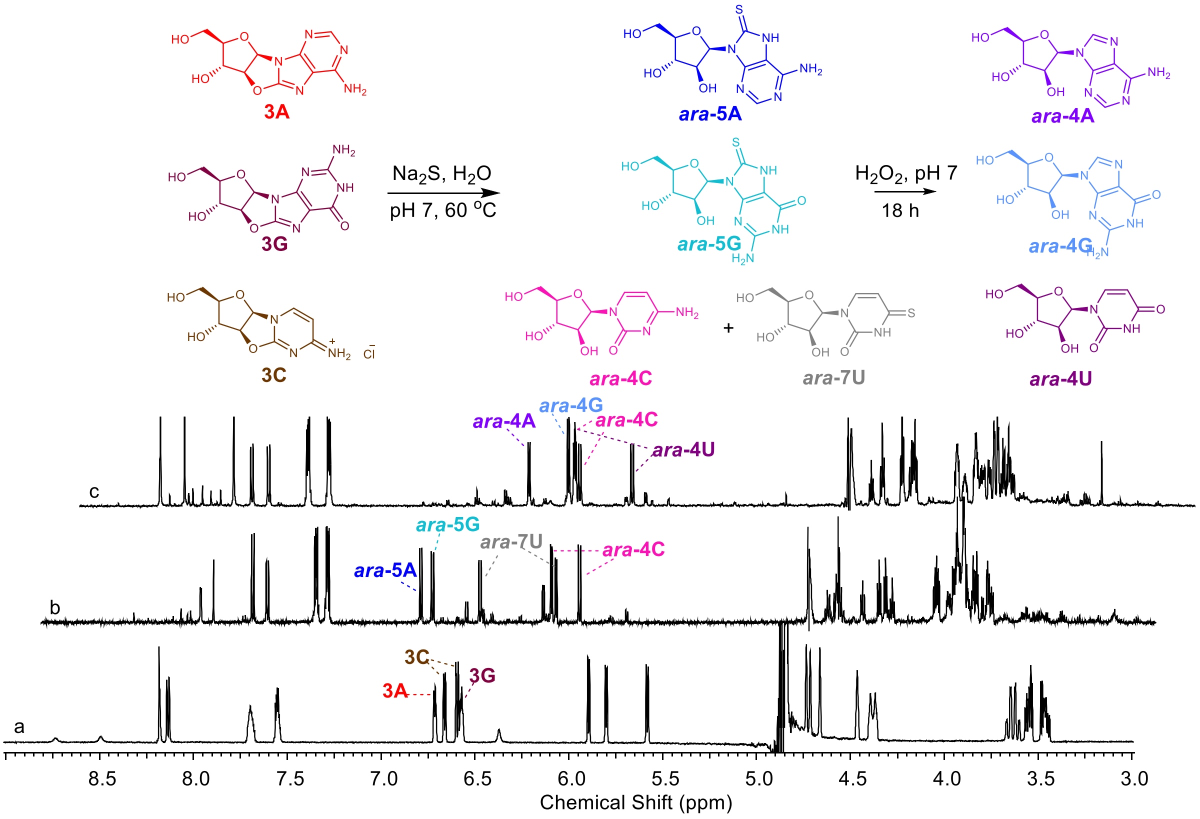

Supplementary Figure 46 | ^{1}H NMR (600 MHz, {H_2O/D_2O 9:1}, noesygppr1d, 3.0–9.0 ppm) spectra showing the sequential reaction of arabino-2′,2-anhydrocytidine 3C (11.9 mM), 2′,8-anhydroadenosine 3A (11.9 mM), 2′,8-anhydroguanosine 3G (11.9 mM), potassium hydrogen phthalate 13 (7.14 mM; internal NMR standard), with Na_2S (714 mM) at pH 7 and 60 °C for 7 d followed by reaction with H_2O_2 (30% w/w, 115 µL, 1.13 mmol) at pH 7 and room temperature for 1 h. a. *Spectrum showing the reaction starting materials.* ***b.*** *Spectrum showing the thiolysis products after 7 d.* ***c.*** *Spectrum showing the thiolysis products following reaction with H_2O_2 (30% w/w, 115 µL, 1.13 mmol) for 1 h.*

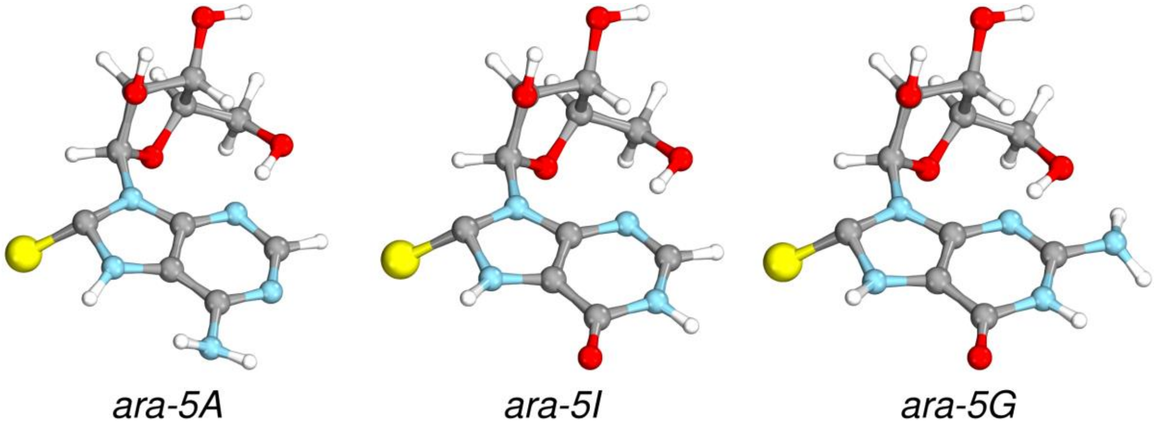

Supplementary Figure 47 | Minimum-energy geometries of the S1(ππCS*) state in all three investigated arabinose mercaptopurines ara-5A, ara-5I and ara-5G.

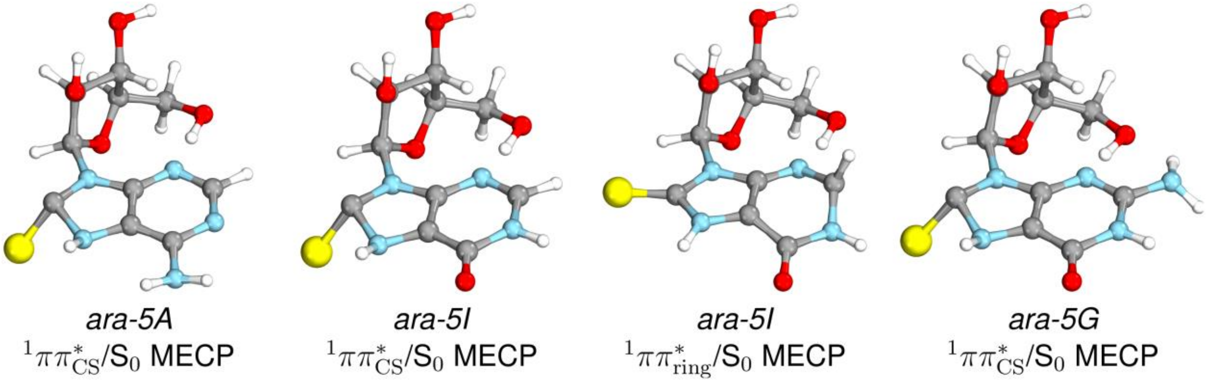

Supplementary Figure 48 | Minimum-energy crossing points for the S1/S0 conical intersections of ara-5A, ara-5I and ara-5G.

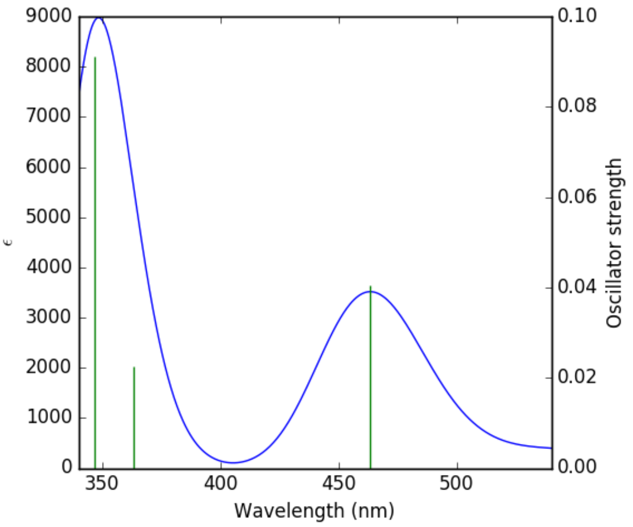

Supplementary Figure 49 | ESA spectrum simulated from the S1(ππCS*) minimum of ara-5I.

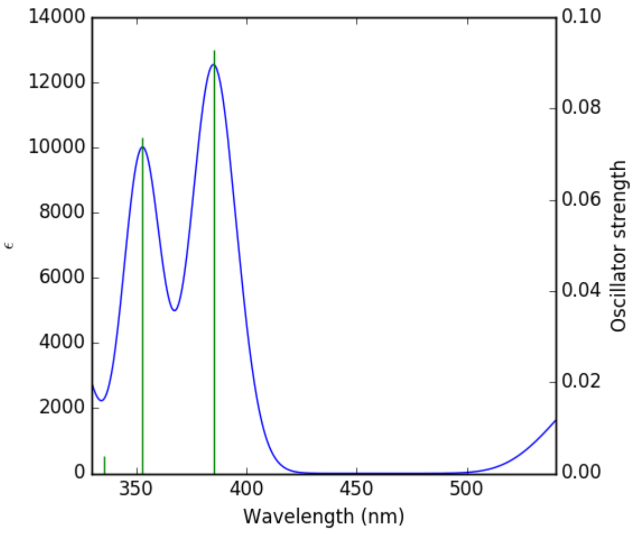

Supplementary Figure 50 | ESA spectrum simulated from the S1(ππring*)/S0 conical intersection of ara-5I. *Since the minimum of the S1(ππring*) state coincides with the corresponding conical intersection with the ground electronic state, the simulated spectrum should be treated as a tentative result.*

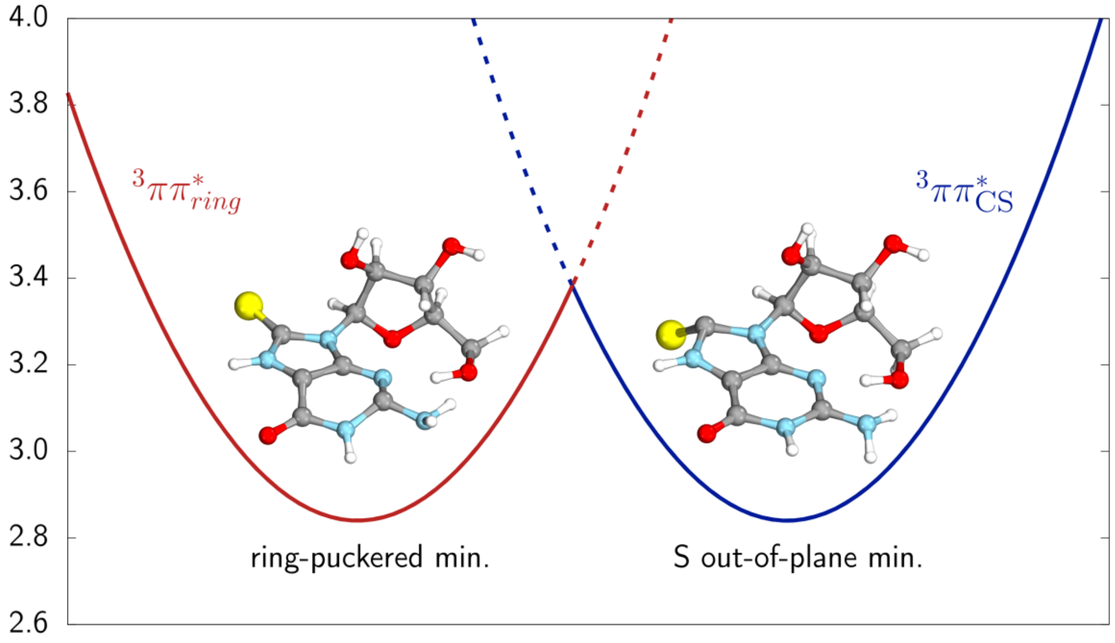

Supplementary Figure 51 | Schematic representation of the T1 topography of ara-5G. *The parabolas were fitted to the computed energies of the T1 minima and the optimized saddle point.*

2′,8-Anhydroadenosine ***3A***

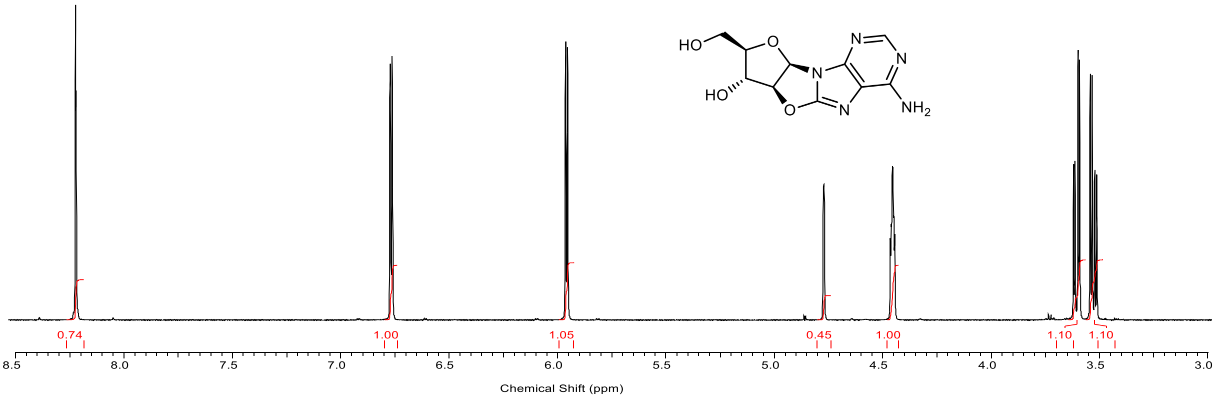

Supplementary Figure 52 | ^{1}H NMR (600 MHz, {H_2O/D_2O 9:1}, noesygppr1d, 3.0–8.5 ppm) spectrum of 2′,8-anhydroadenosine 3A

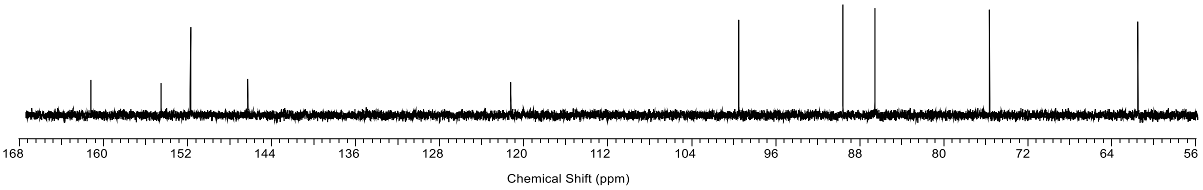

Supplementary Figure 53 | ^{13}C NMR (151 MHz, {D_2O}, 56.0–168.0 ppm) spectrum of 2′,8-anhydroadenosine 3A

2′,8-Anhydroguanosine ***3G***

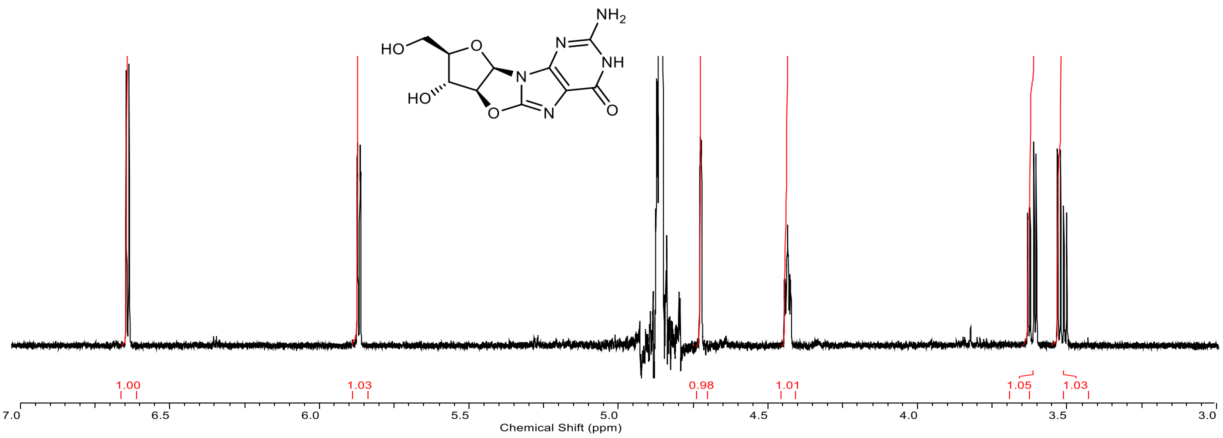

Supplementary Figure 54 | ^{1}H NMR (600 MHz, {H_2O/D_2O 9:1}, noesygppr1d, 3.0–7.0 ppm) spectrum of 2′,8-anhydroguanosine 3G

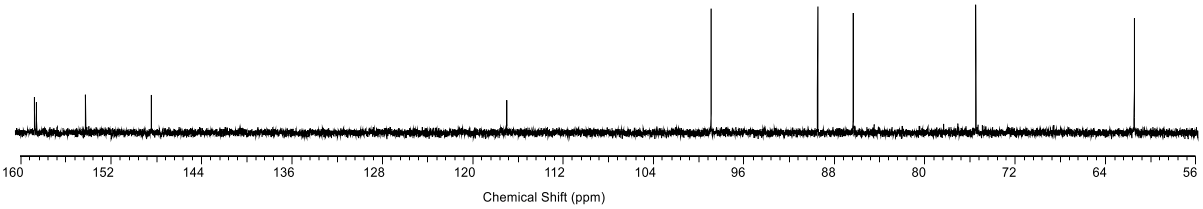

Supplementary Figure 55 | ^{13}C NMR (151 MHz, {D_2O}, 56.0–160.0 ppm) spectrum of 2′,8-anhydroguanosine 3G

2′,8-Anhydroinosine ***3I***

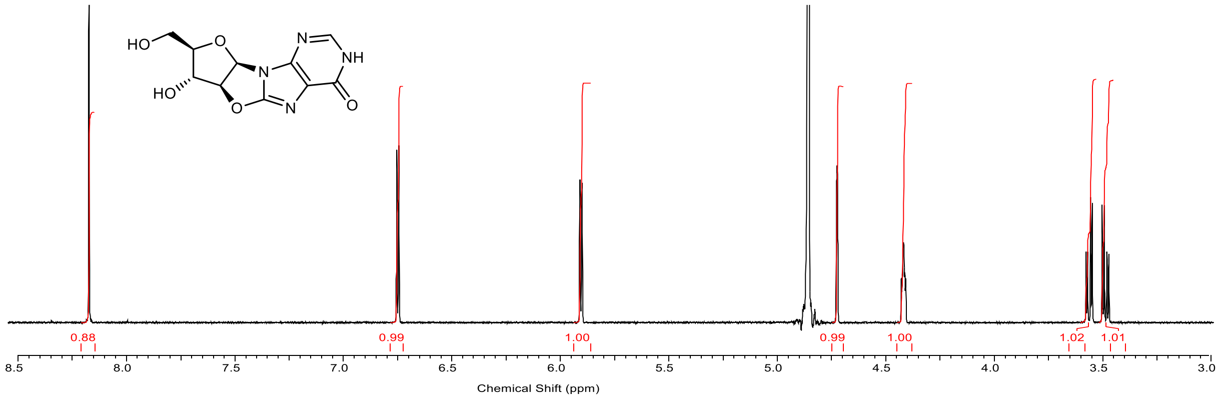

Supplementary Figure 56 | ¹H NMR (600 MHz, {D₂O}, 3.0–8.5 ppm) spectrum of 2′,8-anhydroinosine 3I

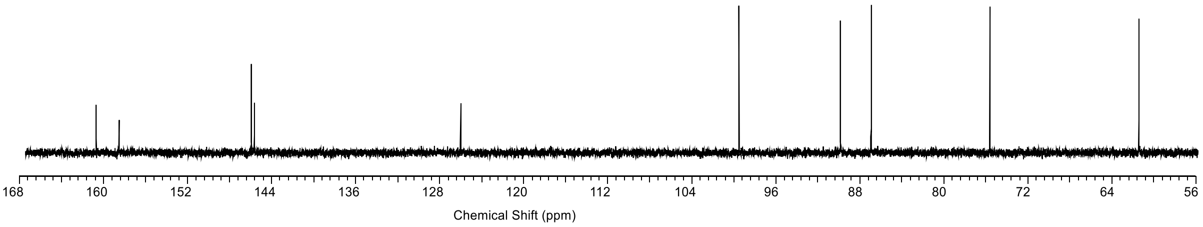

Supplementary Figure 57 | ¹³C NMR (1540 MHz, {D₂O}, 56.0–168.0 ppm) spectrum of 2′,8-anhydroinosine 3I

2′,2-Anhydrocytidine ***3C***

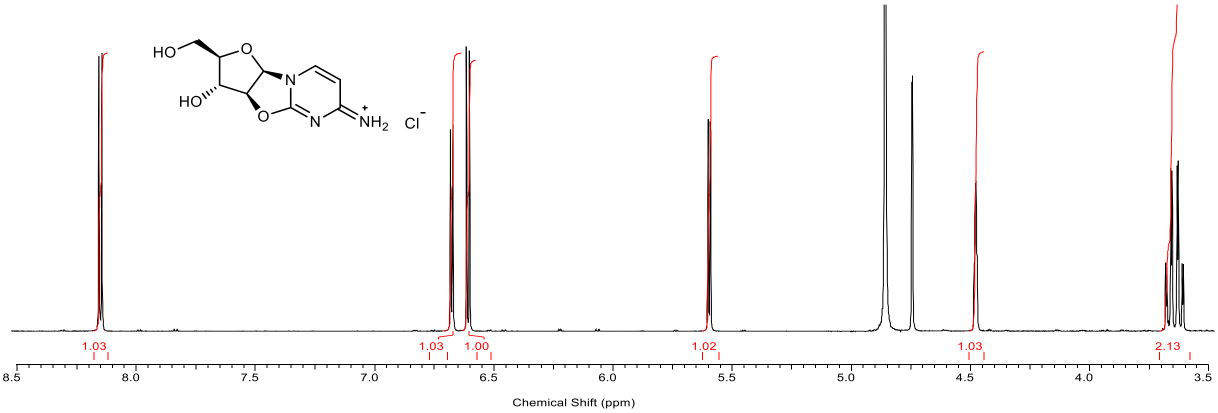

Supplementary Figure 58 | ¹H NMR (600 MHz, {D₂O}, 3.5–8.5 ppm) spectrum of 2′,2-anhydrocytidine 3C

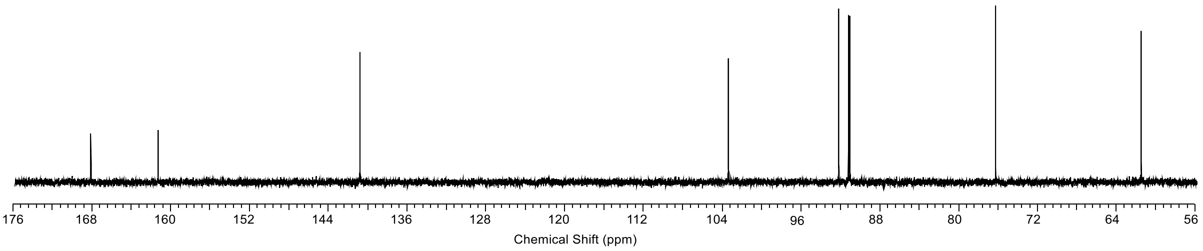

Supplementary Figure 59 | ¹³C NMR (151 MHz, {D₂O}, 56.0–176.0 ppm) spectrum of 2′,2-anhydrocytidine 3C

ribo-2',2-Anhydrocytidine ***α-ribo-3C***

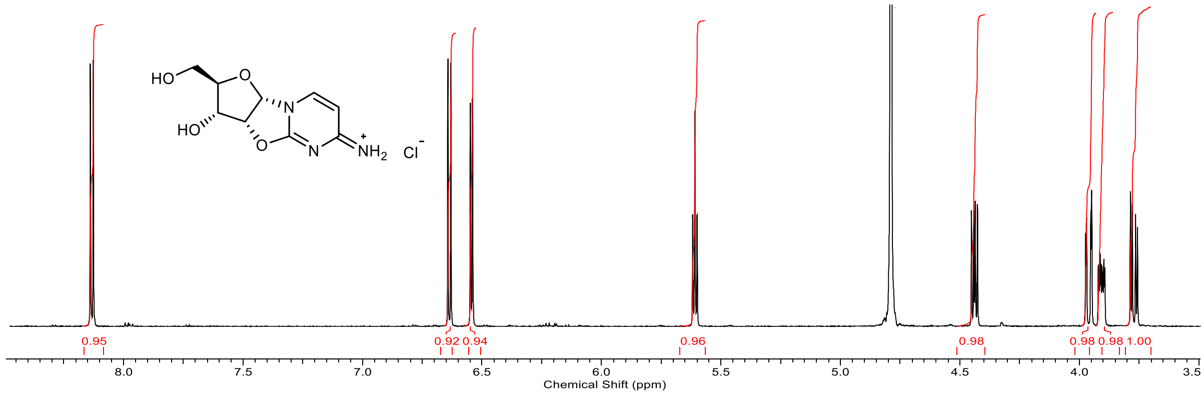

Supplementary Figure 60 | ^{1}H NMR (600 MHz, {D$_2$O}, 3.5–8.5 ppm) spectrum of ribo-2',2-anhydrocytidine α-ribo-3C

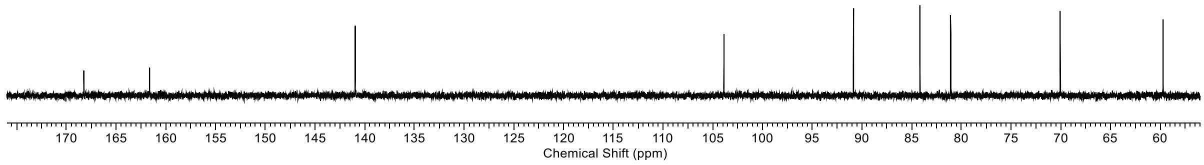

Supplementary Figure 61 | ^{13}C NMR (151 MHz, {D$_2$O}, 56.0–176.0 ppm) spectrum of ribo-2',2-anhydrocytidine α-ribo-3C

arabino-Adenosine ***ara-4A***

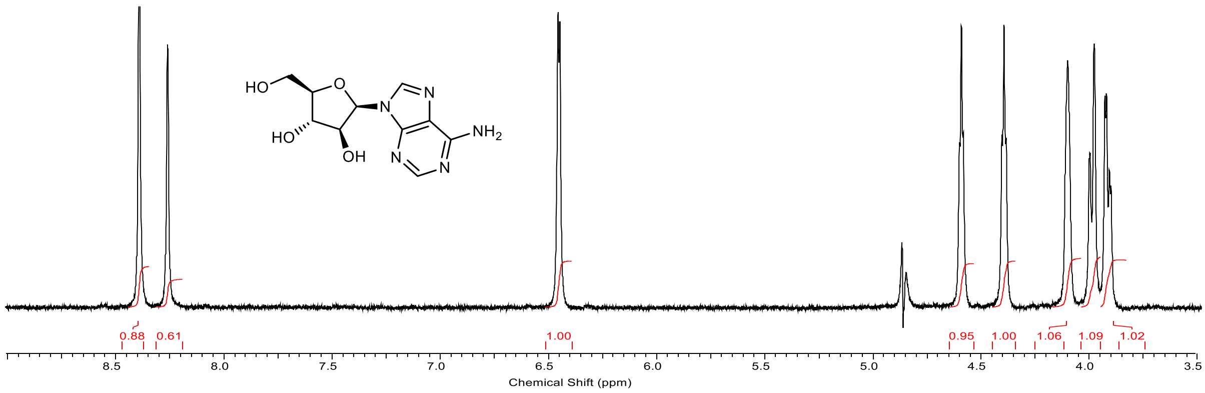

Supplementary Figure 62 | ^{1}H NMR (600 MHz, {D$_2$O}, noesygppr1d, 3.5–9.0 ppm) spectrum of arabino-adenosine ara-4A

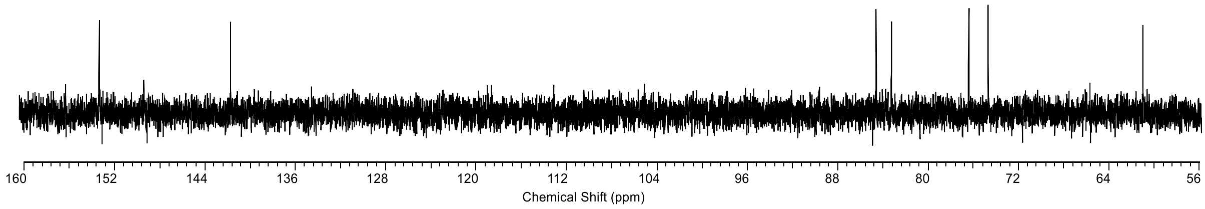

Supplementary Figure 63 | ^{13}C NMR (151 MHz, {D$_2$O}, 56.0–160.0 ppm) spectrum of arabino-adenosine ara-4A

arabino-Guanosine ***ara*-4G**

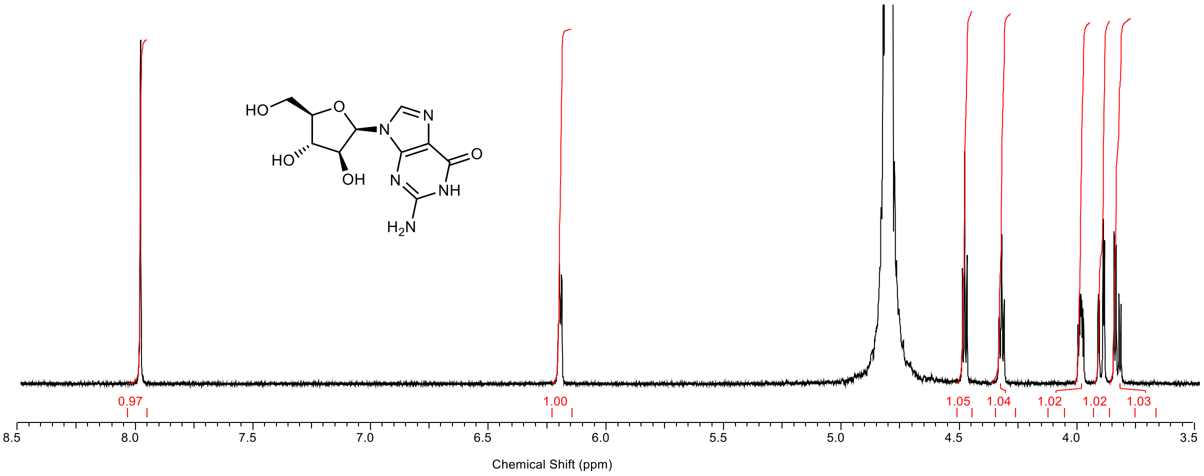

Supplementary Figure 64 | ***^{1}H NMR (600 MHz, {D₂O}, 3.5–8.5 ppm) spectrum of arabino-guanosine ara-4G***

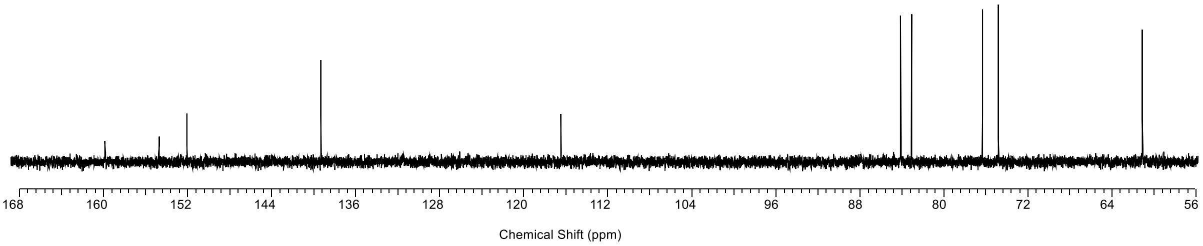

Supplementary Figure 65 | ***^{13}C NMR (151 MHz, {D₂O}, 56.0–168.0 ppm) spectrum of arabino-guanosine ara-4G***

arabino-Inosine ***ara*-4I**

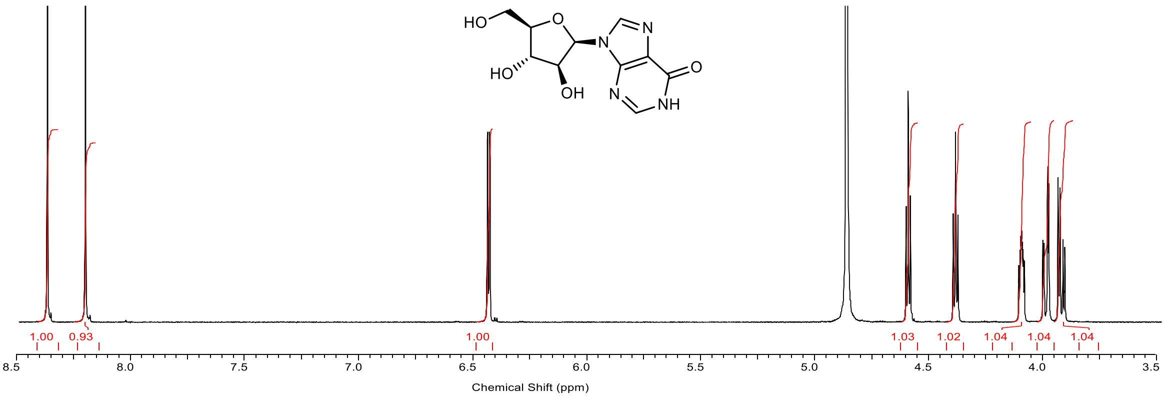

Supplementary Figure 66 | ***^{1}H NMR (600 MHz, {D₂O}, 3.5–8.5 ppm) spectrum of arabino-inosine ara-4I***

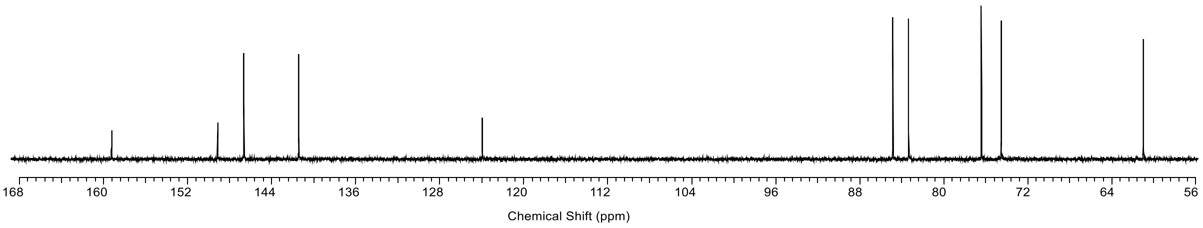

Supplementary Figure 67 | ***^{13}C NMR (151 MHz, {D₂O}, 56.0–168.0 ppm) spectrum of arabino-inosine ara-4I***

arabino-Cytidine ***ara-4C***

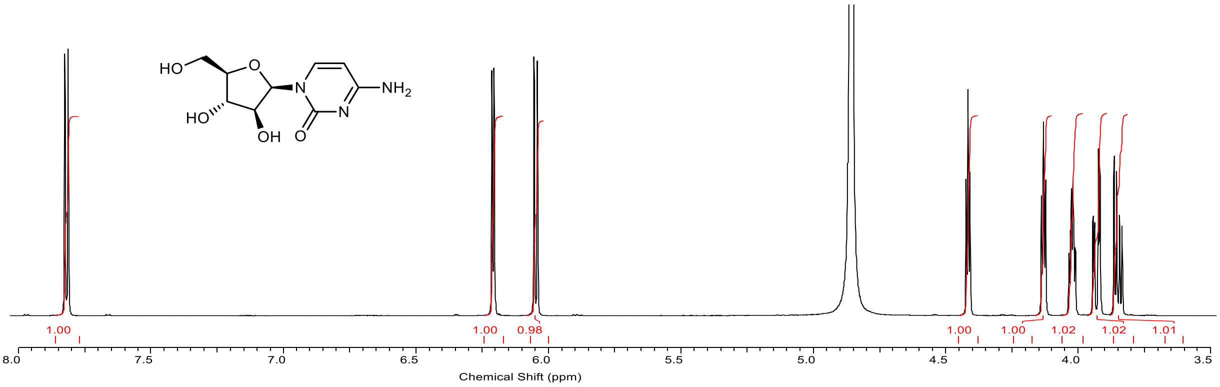

Supplementary Figure 68 | ¹H NMR (600 MHz, {D₂O}, 3.5–8.0 ppm) spectrum of arabino-cytidine ara-4C

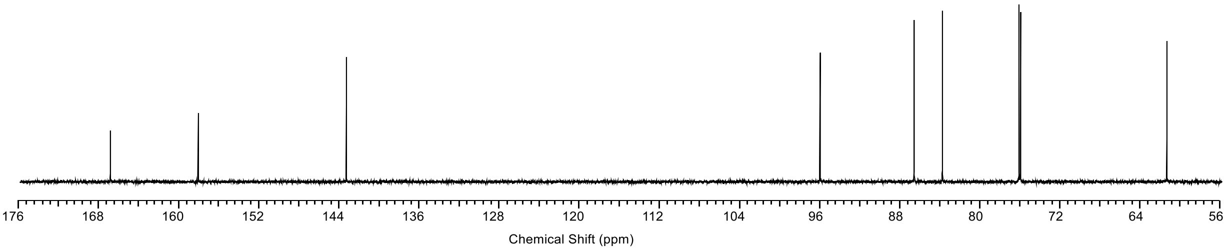

Supplementary Figure 69 | ¹³C NMR (151 MHz, {D₂O}, 56.0–176.0 ppm) spectrum of arabino-cytidine ara-4C

arabino-Uridine ***ara-4U***

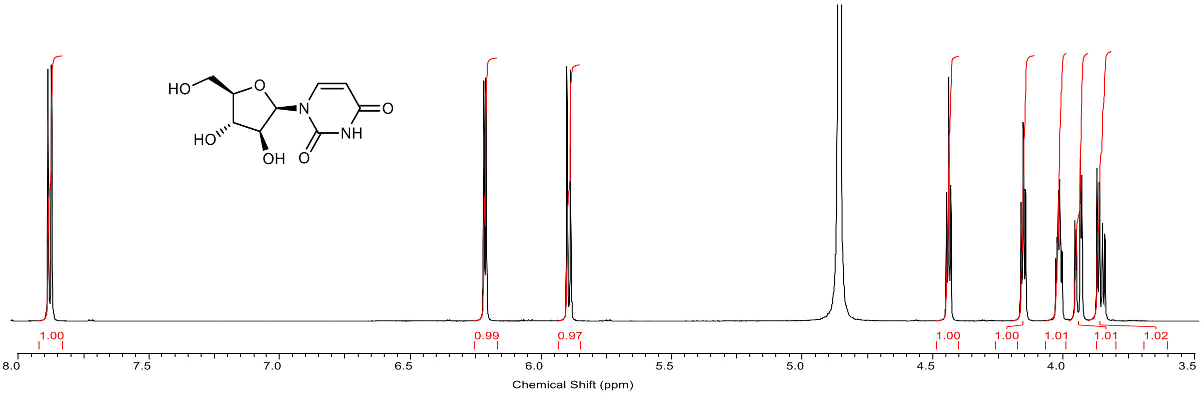

Supplementary Figure 70 | ¹H NMR (600 MHz, {D₂O}, 3.5–8.0 ppm) spectrum of arabino-uridine ara-4U

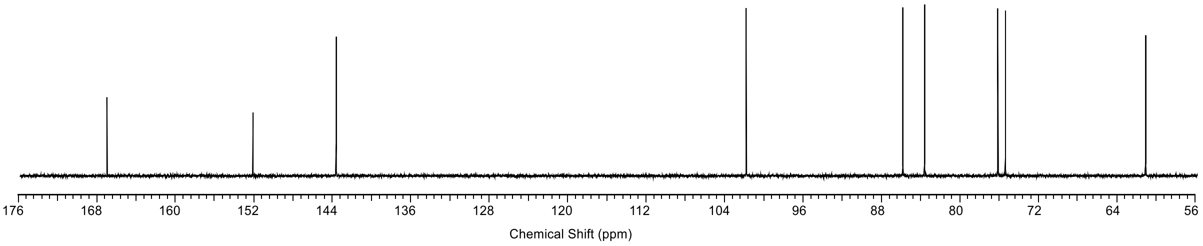

Supplementary Figure 71 | ¹³C NMR (151 MHz, {D₂O}, 56.0–176.0 ppm) spectrum of arabino-uridine ara-4U

*α-ribo-Cytidine **α-ribo-4C***

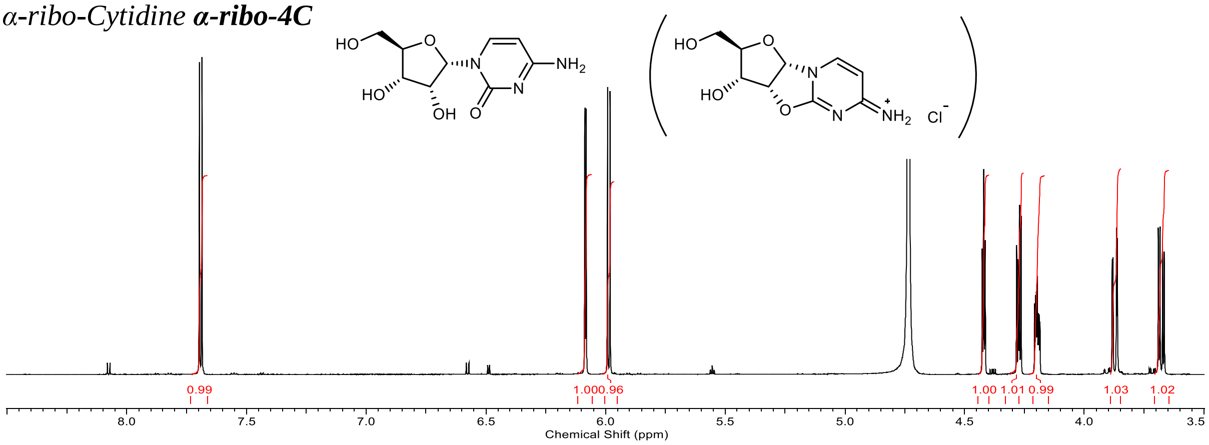

Supplementary Figure 72 | ^{1}H NMR (600 MHz, {D₂O}, 3.5–8.5 ppm) spectrum of α-ribo-cytidine α-ribo-4C, with ribo-2′,2-anhydrocytidine α-ribo-3C impurity (4%)

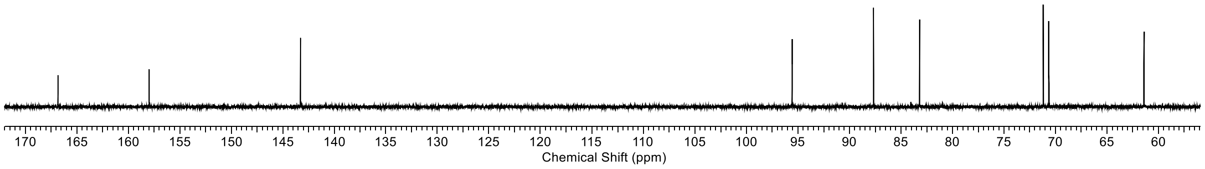

Supplementary Figure 73 | ^{13}C NMR (151 MHz, {D₂O}, 56.0–174.0 ppm) spectrum of α-ribo-cytidine α-ribo-4C

*8-Mercapto-arabino-adenosine **ara-5A***

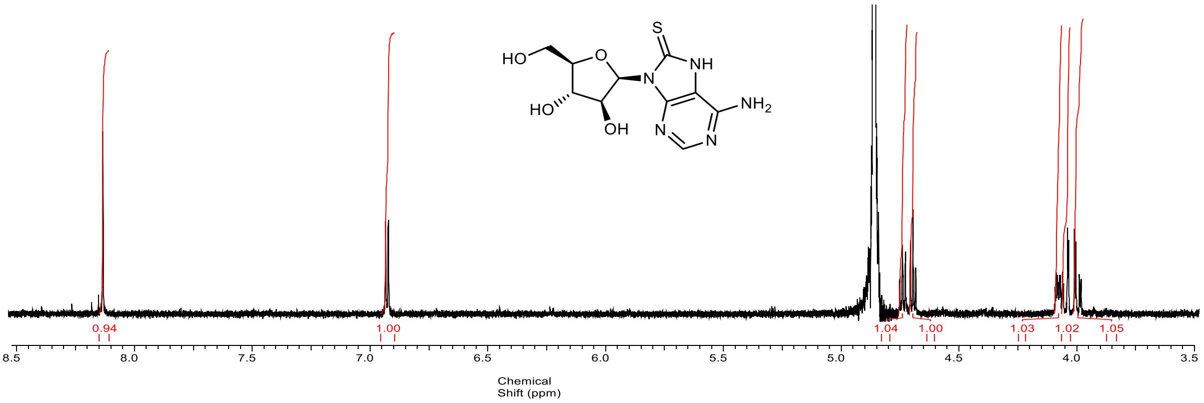

Supplementary Figure 74 | ^{1}H NMR (600 MHz, {D₂O}, 3.5–8.5 ppm) spectrum of 8-mercapto-arabino-adenosine ara-5A.

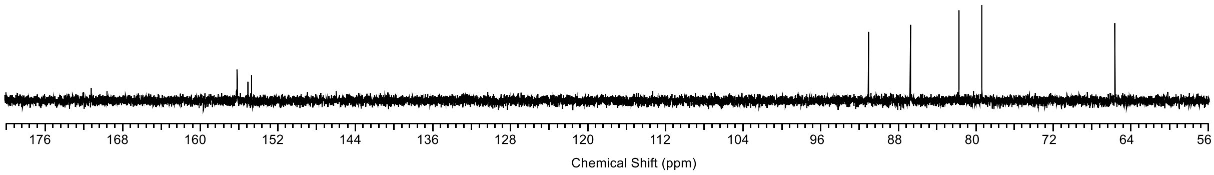

Supplementary Figure 75 | ^{13}C NMR (151 MHz, {D₂O}, 56.0–180.0 ppm) spectrum of 8-mercapto-arabino-adenosine ara-5A

8-Mercapto-arabino-guanosine ***ara-5G***

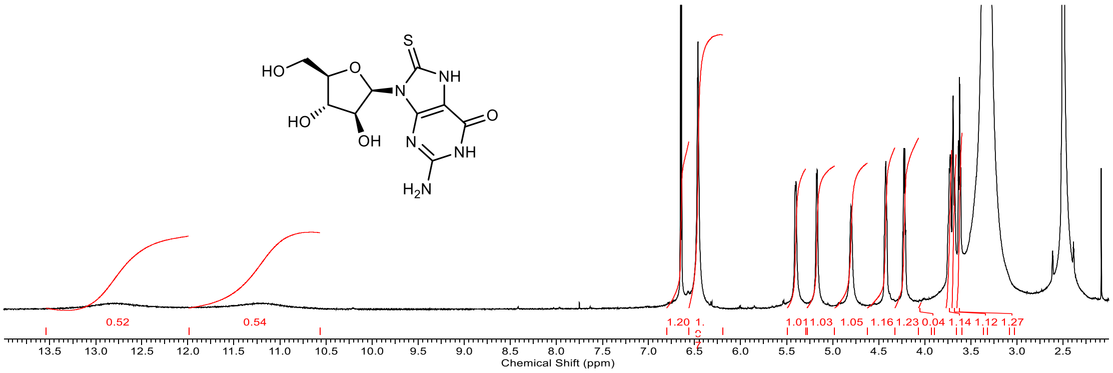

Supplementary Figure 76 | ***^{1}H NMR (600 MHz, {d6-DMSO}, 2.0–14.0 ppm) spectrum of 8-mercapto-arabino-guanosine ara-5G.***

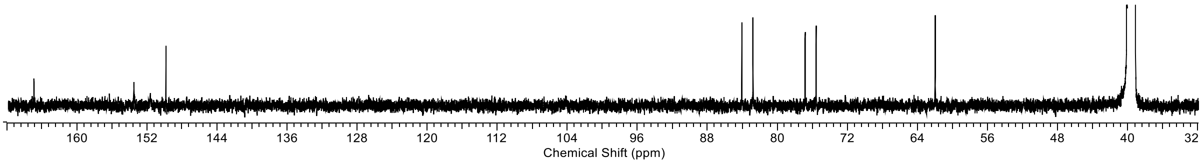

Supplementary Figure 77 | ^{13}C NMR (151 MHz, {d6-DMSO}, 32.0–168.0 ppm) spectrum of 8-mercapto-arabino-guanosine ara-5G

8-Mercapto-arabino-inosine ***ara-5I***

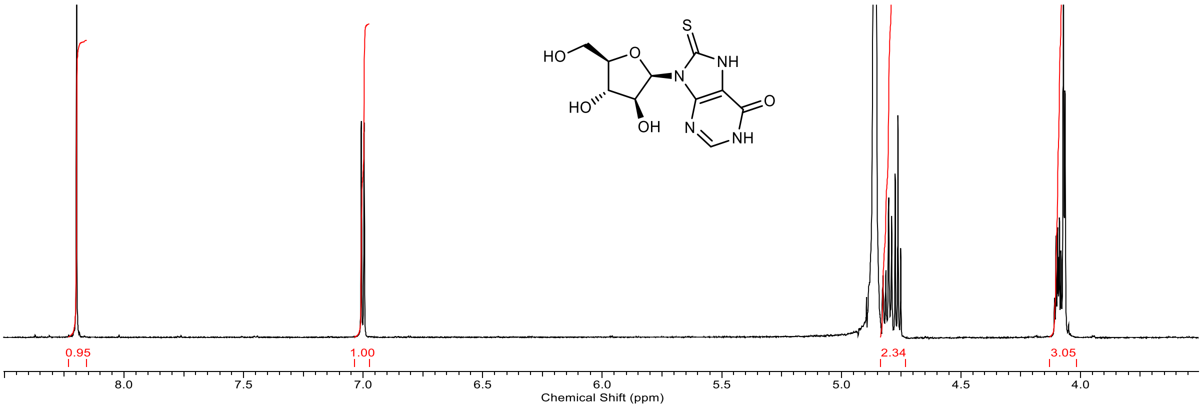

Supplementary Figure 78 | ^{1}H NMR (600 MHz, {D2O}, 3.5–8.5 ppm) spectrum of 8-mercapto-arabino-inosine ara-5I.

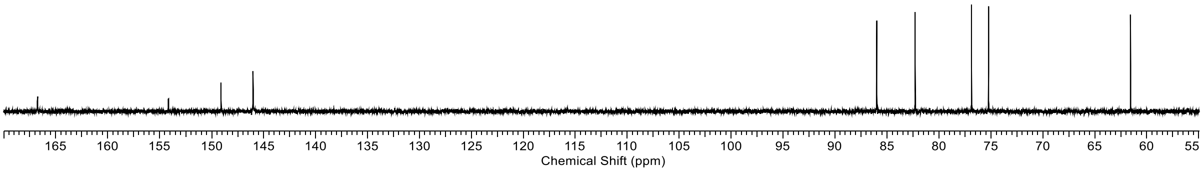

Supplementary Figure 79 | ^{13}C NMR (151 MHz, {D2O}, 55.0–170.0 ppm) spectrum of 8-mercapto-arabino-inosine ara-5I

8-Mercapto-ribo-adenosine ***ribo-5A***

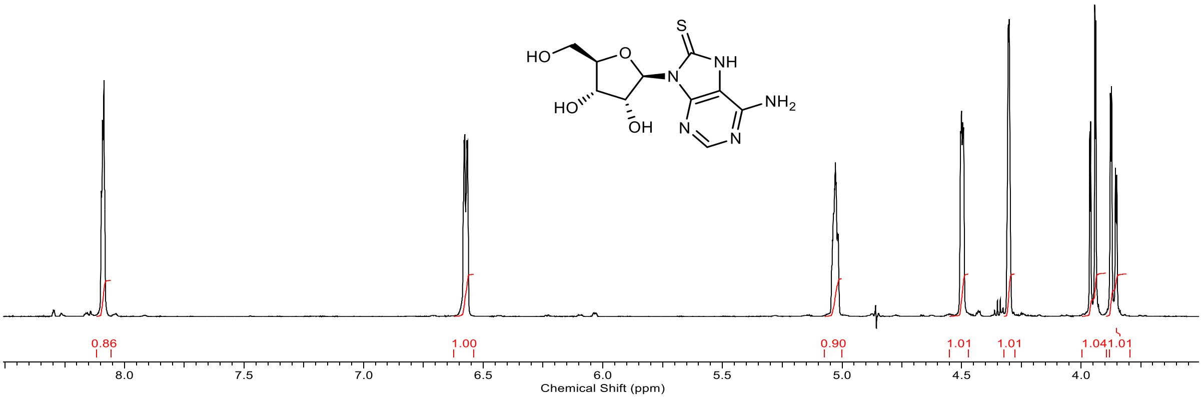

Supplementary Figure 80 | ^{1}H NMR (600 MHz, {D₂O}, 3.5–8.5 ppm) spectrum of 8-mercapto-ribo-adenosine ribo-5A

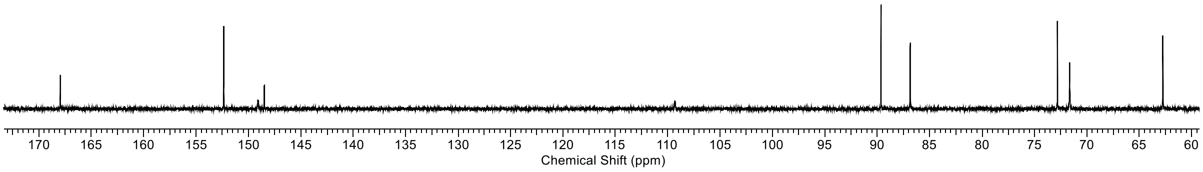

Supplementary Figure 81 | ^{13}C NMR (151 MHz, {D₂O}, 60.0–172.0 ppm) spectrum of 8-mercapto-ribo-adenosine ribo-5A

8-Mercapto-ribo-guanosine ***ribo-5G***

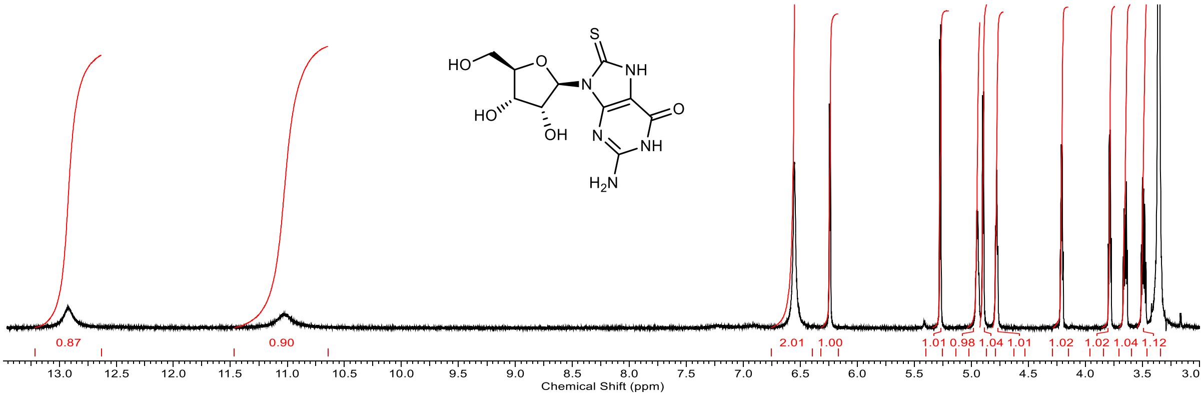

Supplementary Figure 82 | ^{1}H NMR (600 MHz, {DMSO}, 3.0–13.5 ppm) spectrum of 8-mercapto-ribo-guanosine ribo-5G

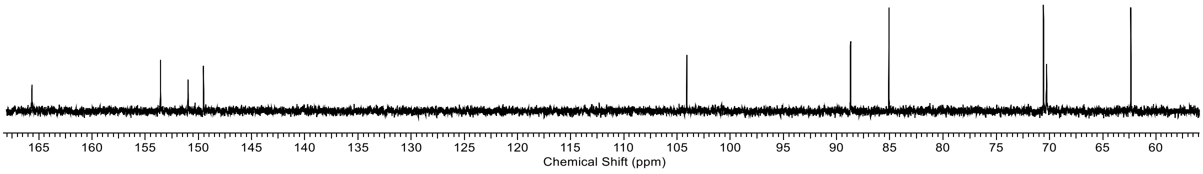

Supplementary Figure 83 | ^{13}C NMR (151 MHz, {DMSO}, 56.0–168.0 ppm) spectrum of 8-mercapto-ribo-guanosine ribo-5G

8-Mercapto-ribo-inosine ***ribo-5I***

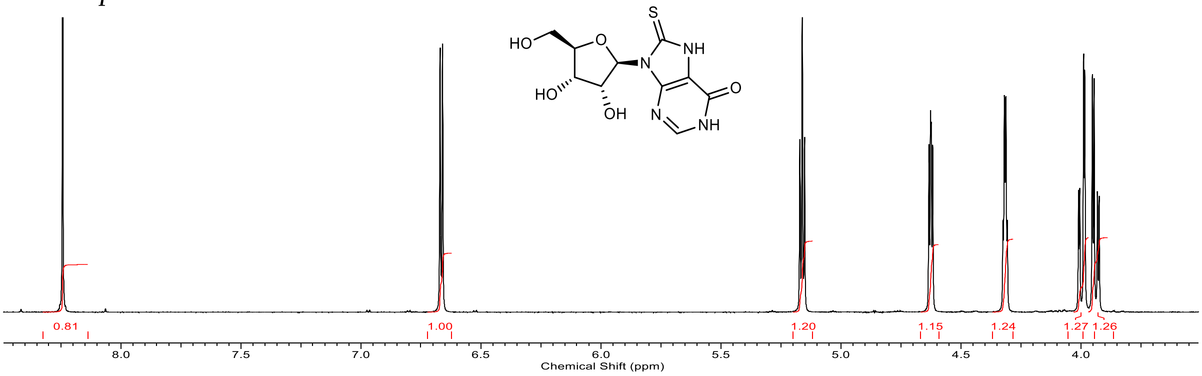

Supplementary Figure 84 | ^{1}H NMR (600 MHz, {D₂O}, 3.5–8.5 ppm) spectrum of 8-mercapto-ribo-inosine ribo-5I.

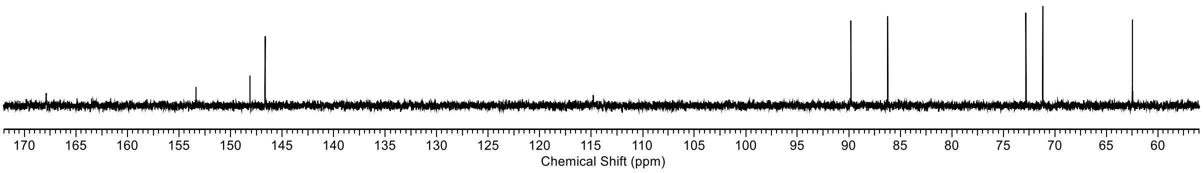

Supplementary Figure 85 | ^{13}C NMR (151 MHz, {D₂O}, 56.0–172.0 ppm) spectrum of 8-mercapto-ribo-inosine ribo-5I.

2-Thio-arabino-cytidine ***ara-6C***

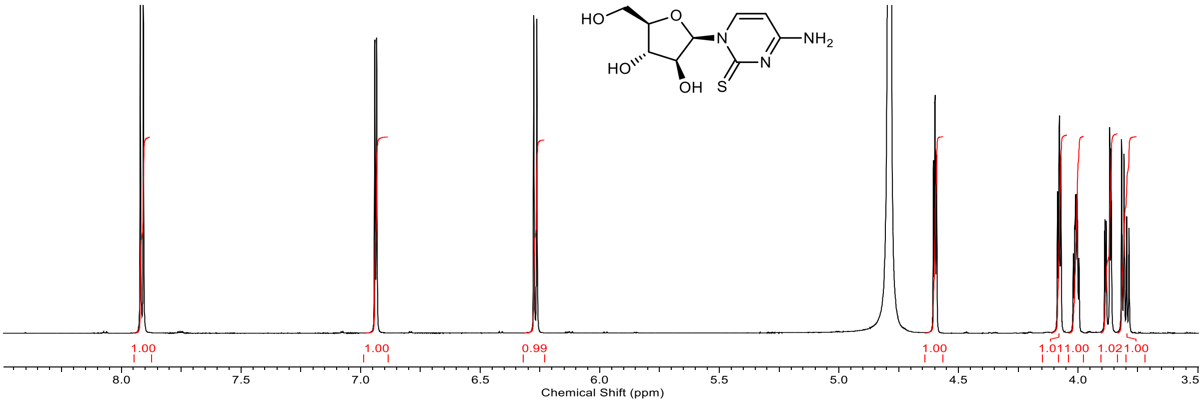

Supplementary Figure 86 | ^{1}H NMR (600 MHz, {D₂O}, 3.5–8.5 ppm) spectrum of 2-thio-arabino-cytidine ara-6C

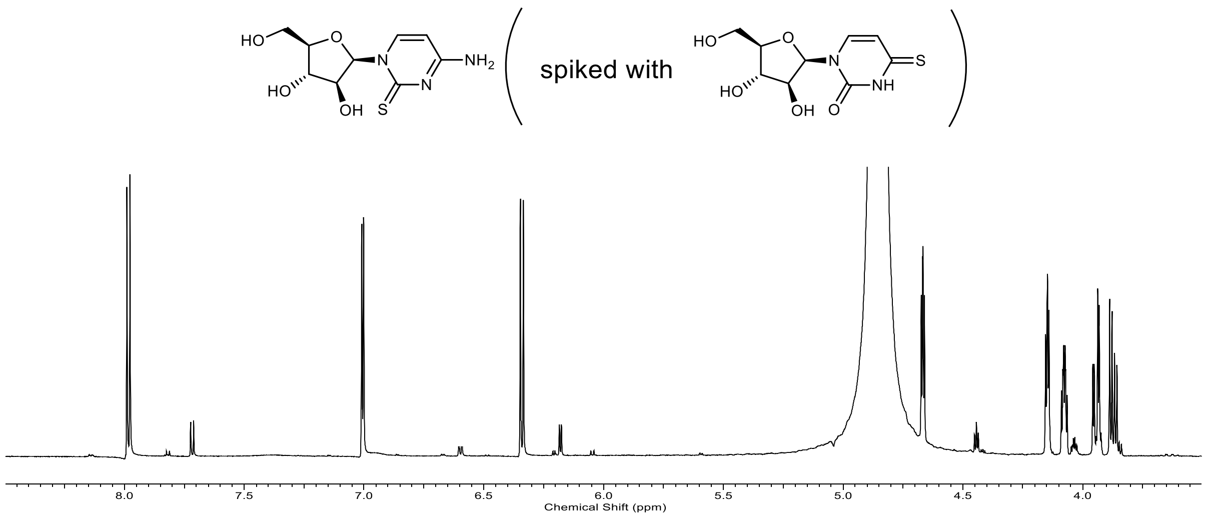

Supplementary Figure 87 | ^{1}H NMR (600 MHz, {D2O}, 3.5–8.5 ppm) spectrum of 2-thio-arabino-cytidine ara-6C spiked with 4-thio-arabino-uridine ara-7U

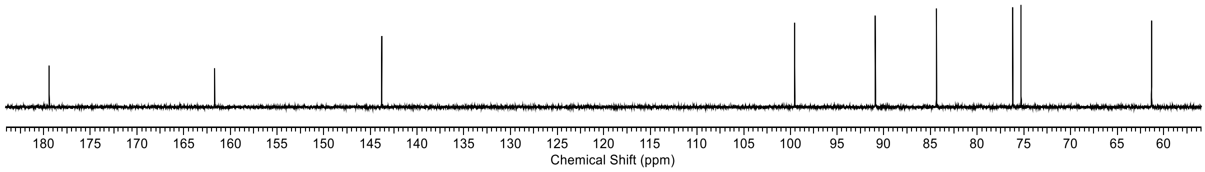

Supplementary Figure 88 | ^{13}C NMR (151 MHz, {D2O}, 56.0–184.0 ppm) spectrum of 2-thio-arabino-cytidine ara-6C

*2-Thio-ribo-cytidine **β-ribo-6C***

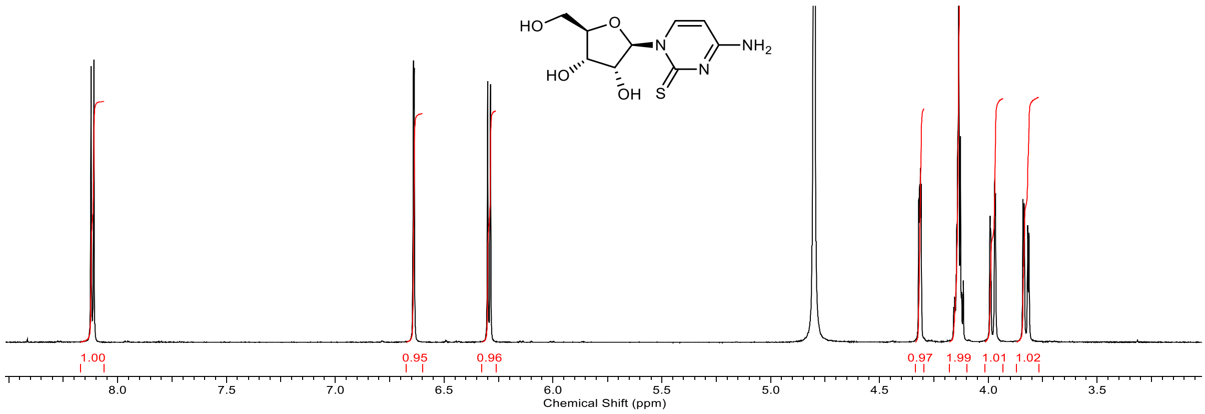

Supplementary Figure 89 | ^{1}H NMR (600 MHz, {D2O}, 3.0–8.5 ppm) spectrum of 2-thio-ribo-cytidine β-ribo-6C.

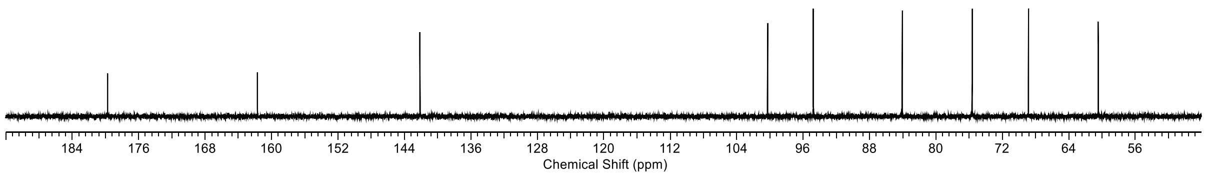

Supplementary Figure 90 | ^{13}C NMR (151 MHz, {D2O}, 48.0–192.0 ppm) spectrum of 2-thio-ribo-cytidine β-ribo-6C

2-Thio-α-ribo-cytidine ***α-ribo-6C***

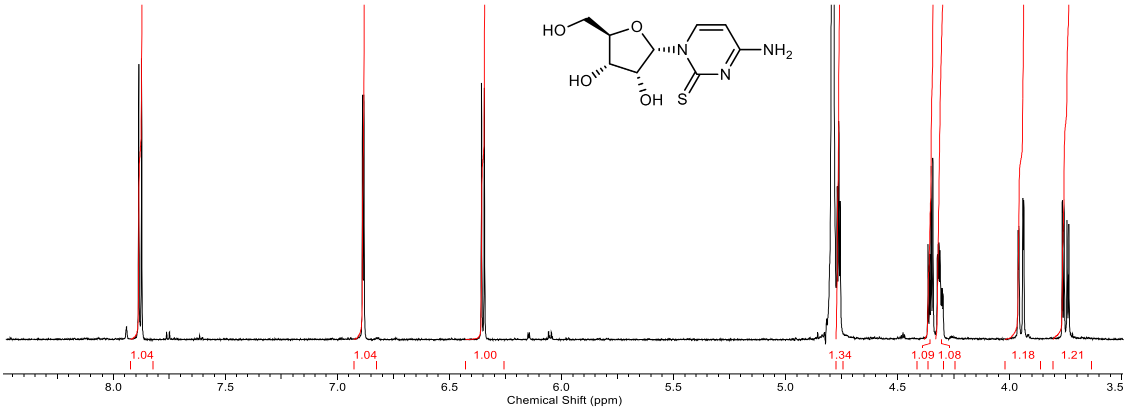

Supplementary Figure 91 | ***^{1}H NMR (600 MHz, {D2O}, 3.5–8.5 ppm) spectrum of 2-thio-α-ribo-cytidine α-ribo-6C.***

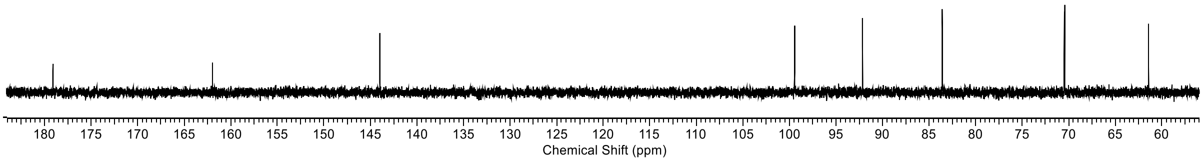

Supplementary Figure 92 | ***^{13}C NMR (151 MHz, {D2O}, 56.0–184.0 ppm) spectrum of 2-thio-α-ribo-cytidine α-ribo-6C***

4-Thio-arabino-uridine ***ara-7U***

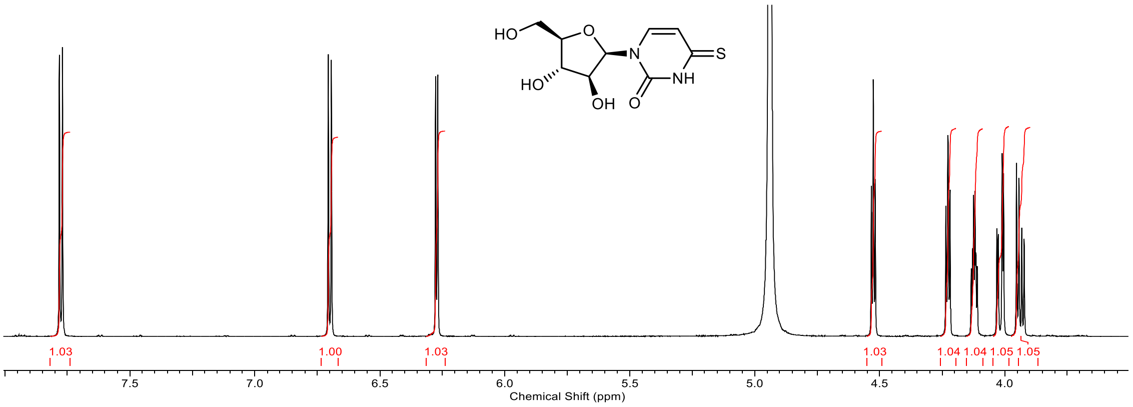

Supplementary Figure 93 | ***^{1}H NMR (600 MHz, {D2O}, 3.5–8.0 ppm) spectrum of 4-thio-arabino-uridine ara-7U***

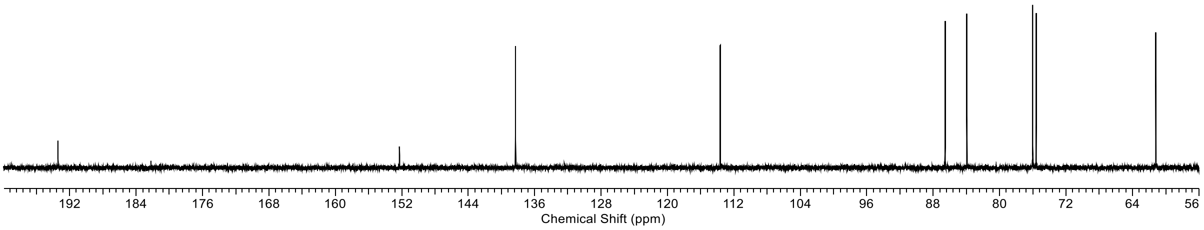

Supplementary Figure 94 | ***^{13}C NMR (151 MHz, {D2O}, 56.0–200.0 ppm) spectrum of 4-thio-arabino-uridine ara-7U***

Presence of sulfur at C4 was confirmed by synthesis of 2-thio-*arabino*-cytidine **ara-6C** and subsequent cross spiking with 4-thio-*arabino*-uridine **ara-7U** (Supplementary Figure 88).

*4-Thio-ribo-uridine **β-ribo-7U***

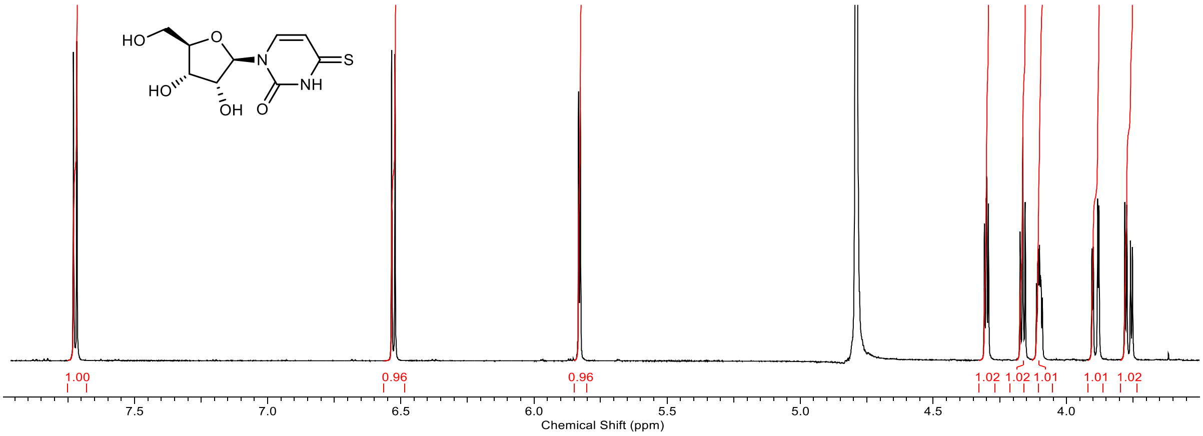

Supplementary Figure 95 | ¹H NMR (600 MHz, {D₂O}, 3.5–8.0 ppm) spectrum of 4-thio-ribo-uridine β-ribo-7U

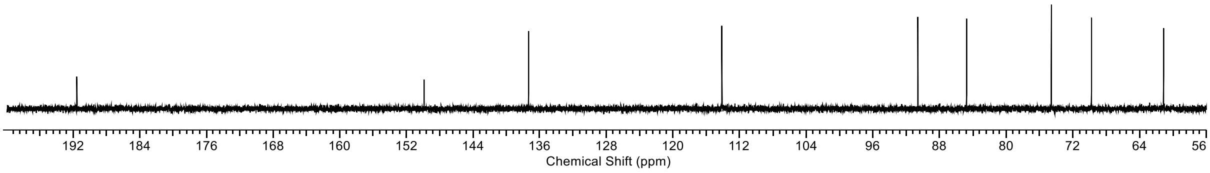

Supplementary Figure 96 | ¹³C NMR (151 MHz, {D₂O}, 56.0-200.0 ppm) spectrum of 4-thio-ribo-uridine β-ribo-7U

*4-thio-α-ribo-uridine **α-ribo-7U***

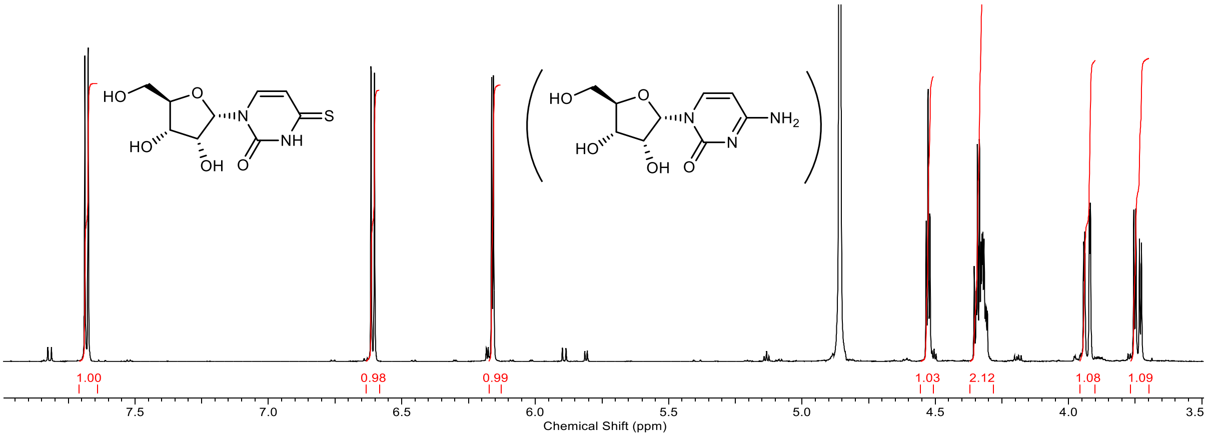

Supplementary Figure 97 | ¹H NMR (600 MHz, {D₂O}, 3.5–8.0 ppm) spectrum of 4-thio-α-ribo-uridine α-ribo-7U, with α-ribo-cytidine α-ribo-4C (5%)

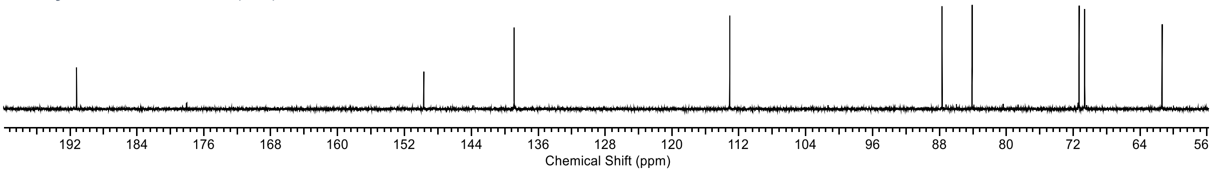

Supplementary Figure 98 | ¹³C NMR (151 MHz, {D₂O}, 56.0-200.0 ppm) spectrum of 4-thio-α-ribo-uridine α-ribo-7U, with α-ribo-cytidine α-ribo-4C (5%)

arabino-Oxazolidinone ***8***

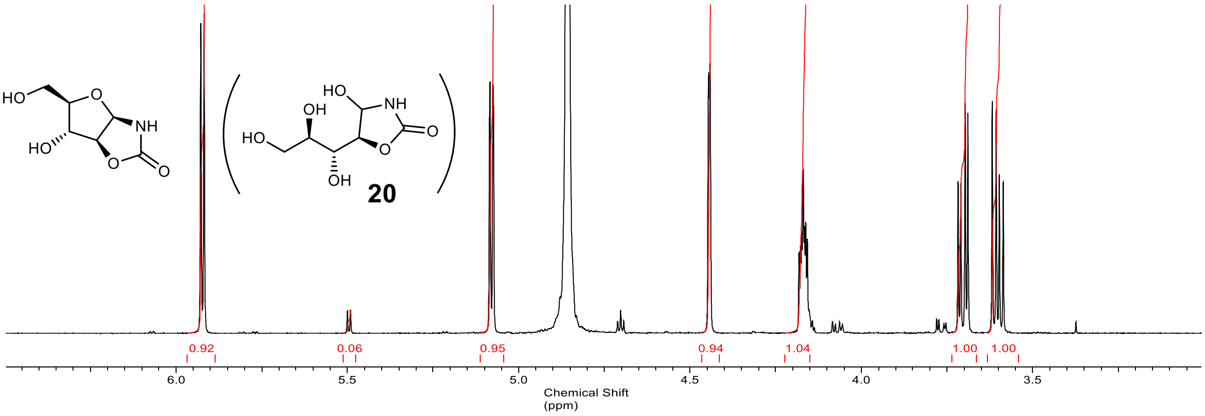

Supplementary Figure 99 | ***^{1}H NMR (600 MHz, {D_2O}, 3.0–6.5 ppm) spectrum of arabino-oxazolidinone 8 with open chain arabino-oxazolidinone 20 (6%)***

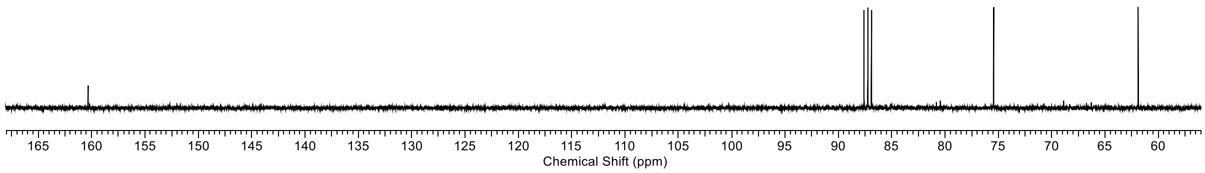

Supplementary Figure 100 | ***^{13}C NMR (151 MHz, {DMSO}, 56.0–168.0 ppm) spectrum of arabino-oxazolidinone 8 with open chain arabino-oxazolidinone 20 (6%)***

5′,8-Anhydroadenosine ***10A***

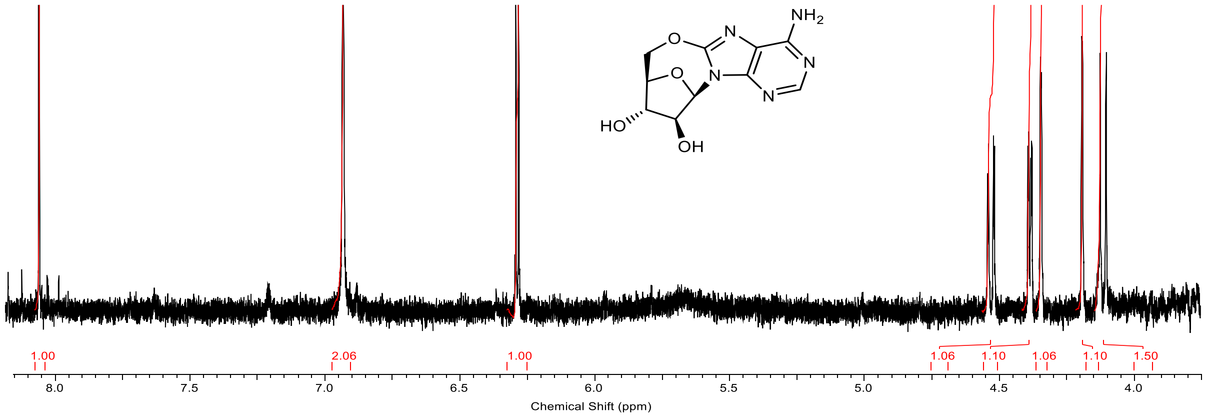

Supplementary Figure 101 | ***^{1}H NMR (600 MHz, {DMSO}, 3.8–8.2 ppm) spectrum of 5′,8-anhydroadenosine 10A***

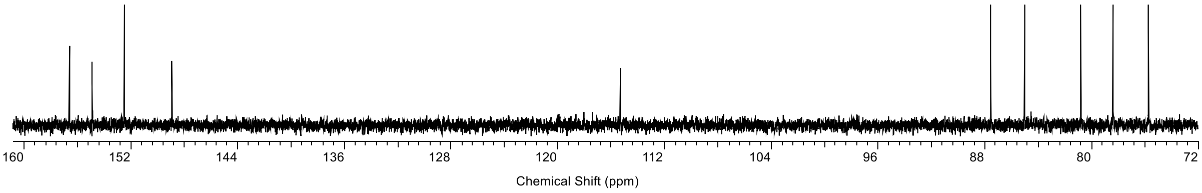

Supplementary Figure 102 | ***^{13}C NMR (151 MHz, {DMSO}, 72.0–160.0 ppm) spectrum of 5′,8-anhydroadenosine 10A***

8-oxo-3′,3-Anhydroguanosine ***12***

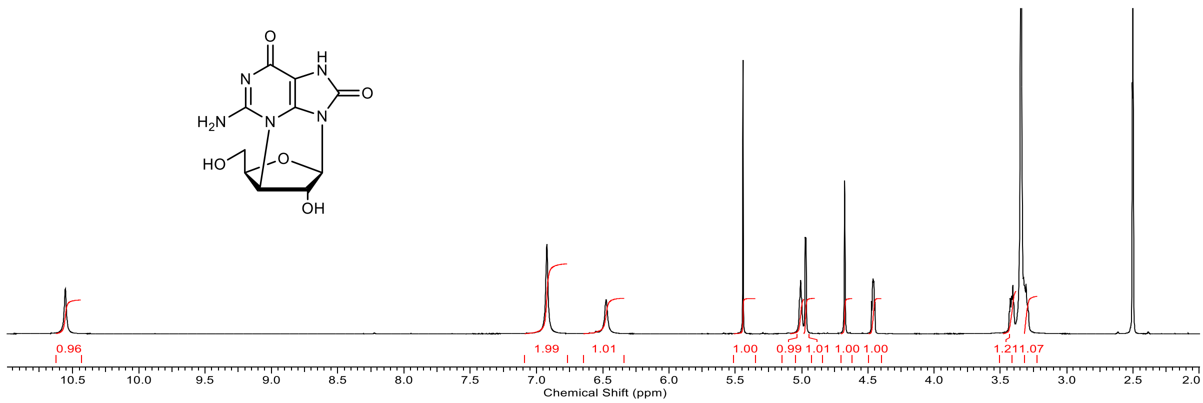

***Supplementary Figure 103** | ^{1}H NMR (600 MHz, {DMSO}, 2.0–11.0 ppm) spectrum of 8-oxo-3′,3-anhydroguanosine 12*

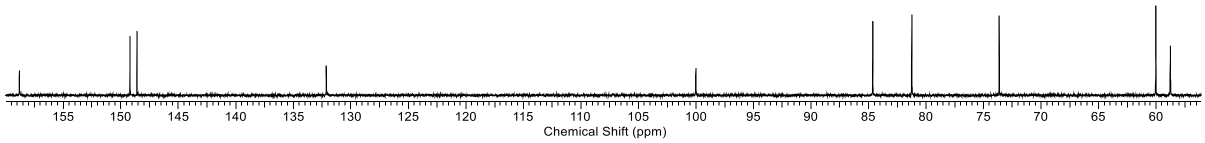

***Supplementary Figure 104** | ^{13}C NMR (151 MHz, {DMSO}, 56.0–160.0 ppm) spectrum of 8-oxo-3′,3-anhydroguanosine 12*

8-Bromo-arabino-adenosine ***ara-16A***

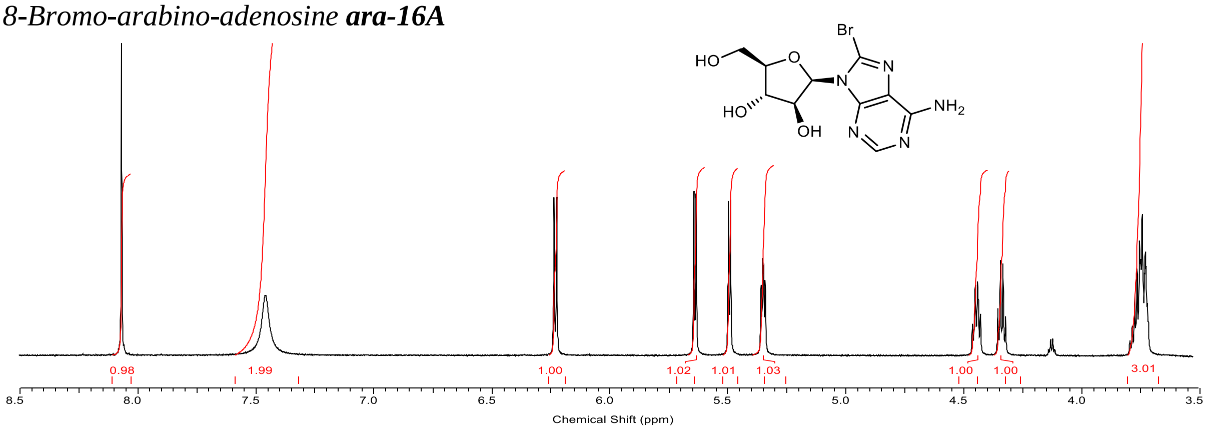

***Supplementary Figure 105** | ^{1}H NMR (600 MHz, {DMSO}, 3.5–8.5 ppm) spectrum of 8-bromo-arabino-adenosine ara-16A*

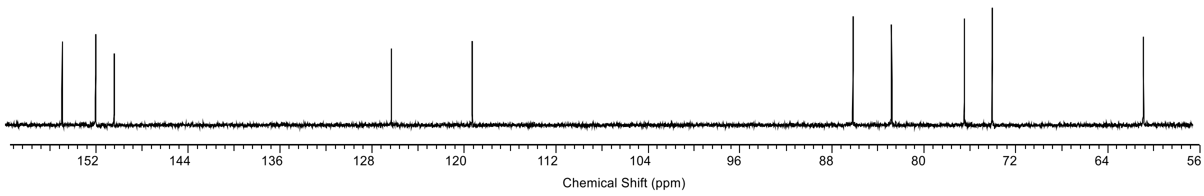

***Supplementary Figure 106** | ^{13}C NMR (151 MHz, {DMSO}, 56.0–160.0 ppm) spectrum of 8-bromo-arabino-adenosine ara-16A*

8-Bromo-arabino-guanosine ***ara-16G***

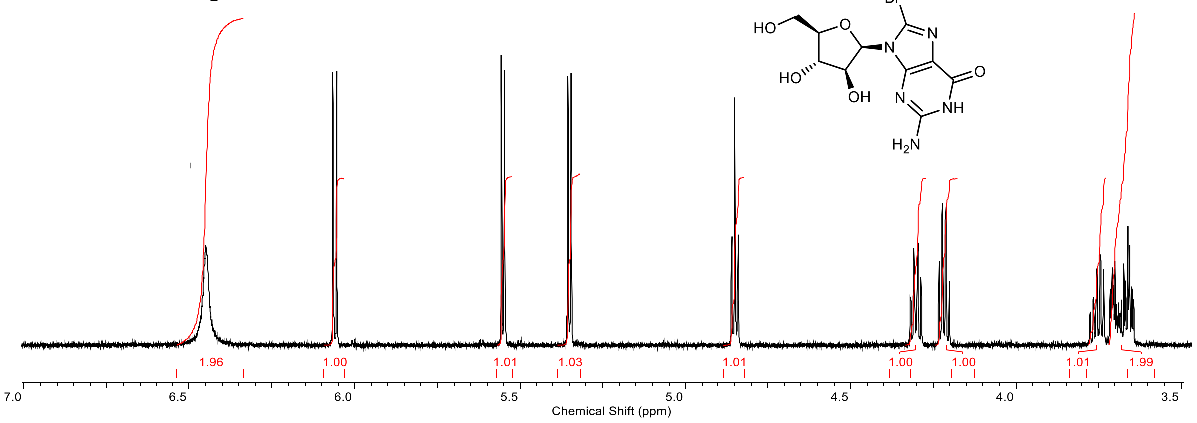

Supplementary Figure 107 | ¹H NMR (600 MHz, {DMSO}, 3.5–7.0 ppm) spectrum of 8-bromo-arabino-guanosine ara-16G

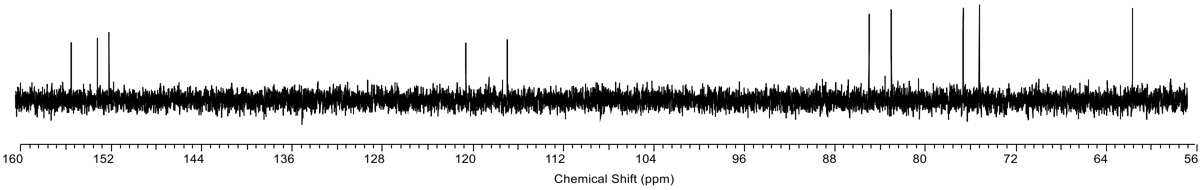

Supplementary Figure 108 | ¹³C NMR (151 MHz, {DMSO}, 56.0–160.0 ppm) of 8-bromo-arabino-guanosine ara-16G

8-Bromo-ribo-adenosine ***ribo-16A***

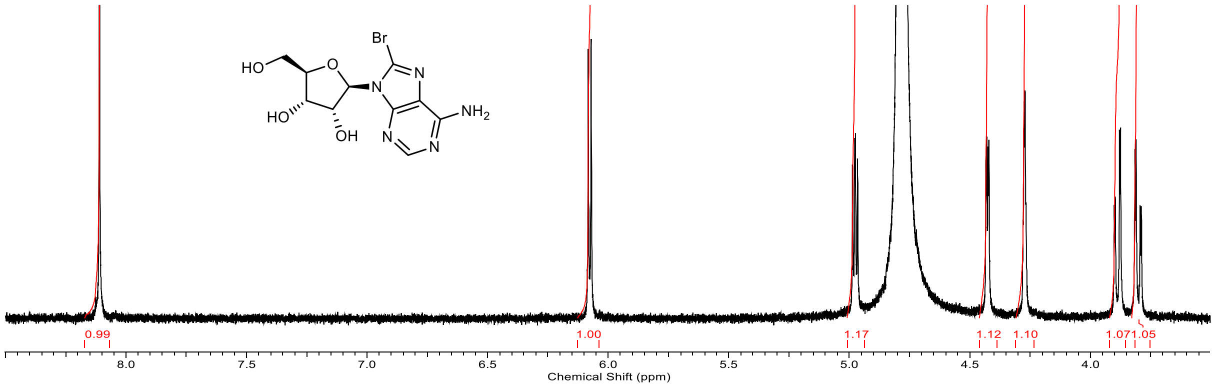

Supplementary Figure 109 | ¹H NMR (600 MHz, {D₂O}, 3.5–8.5 ppm) spectrum of 8-bromo-ribo-adenosine ribo-16A.

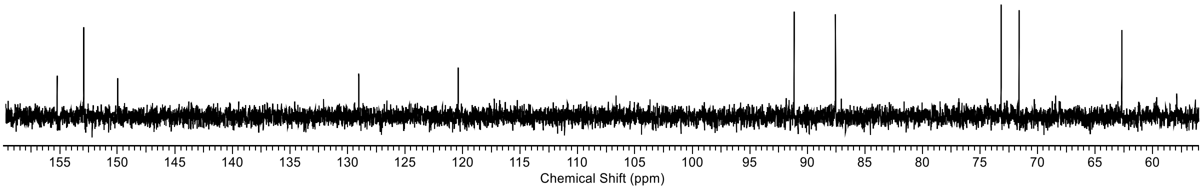

Supplementary Figure 110 | ¹³C NMR (151 MHz, {D₂O}, 56.0–160.0 ppm) spectrum of 8-bromo-ribo-adenosine ribo-16A

8-Bromo-ribo-guanosine ***ribo-16G***

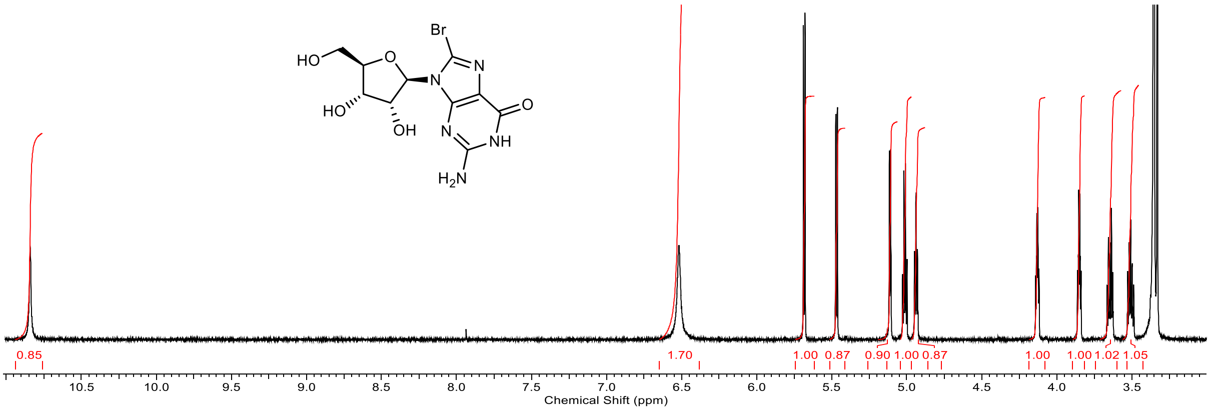

Supplementary Figure 111 | ^{1}H NMR (600 MHz, {DMSO}, 3.0–11.0 ppm) spectrum of 8-bromo-ribo-guanosine ribo-16G.

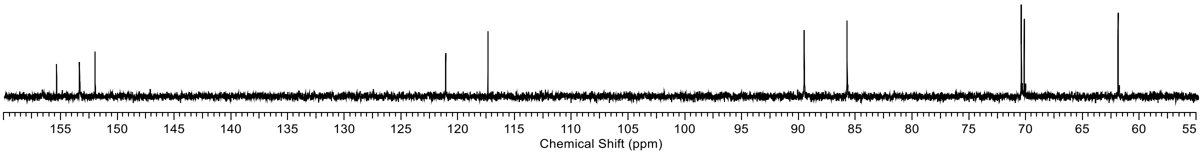

Supplementary Figure 112 | ^{13}C NMR (151 MHz, {DMSO}, 55.0–160.0 ppm) spectrum of 8-bromo-ribo-guanosine ribo-16G

Tri-acetyl-ribo-inosine ***17***

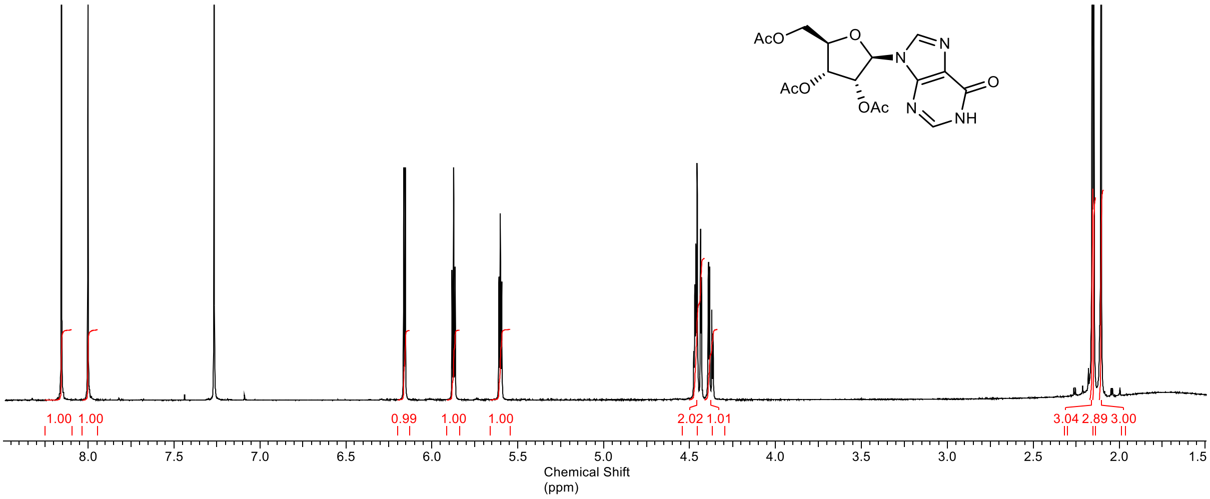

Supplementary Figure 113 | ^{1}H NMR (600 MHz, {$CDCl_3$}, 1.5–8.5 ppm) spectrum of tri-acetyl-ribo-inosine 17.

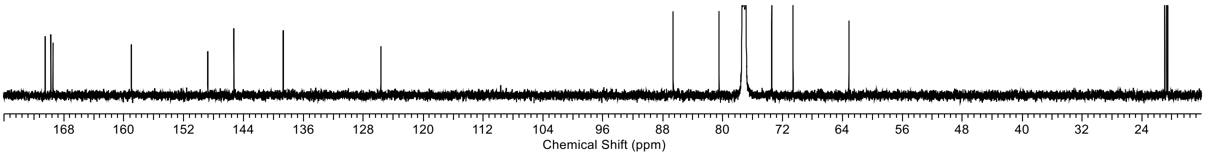

Supplementary Figure 114 | ^{13}C NMR (151 MHz, {D_2O}, 16.0–176.0 ppm) spectrum of tri-acetyl-ribo-inosine 17.

Tri-acetyl-8-bromo-ribo-inosine ***18***

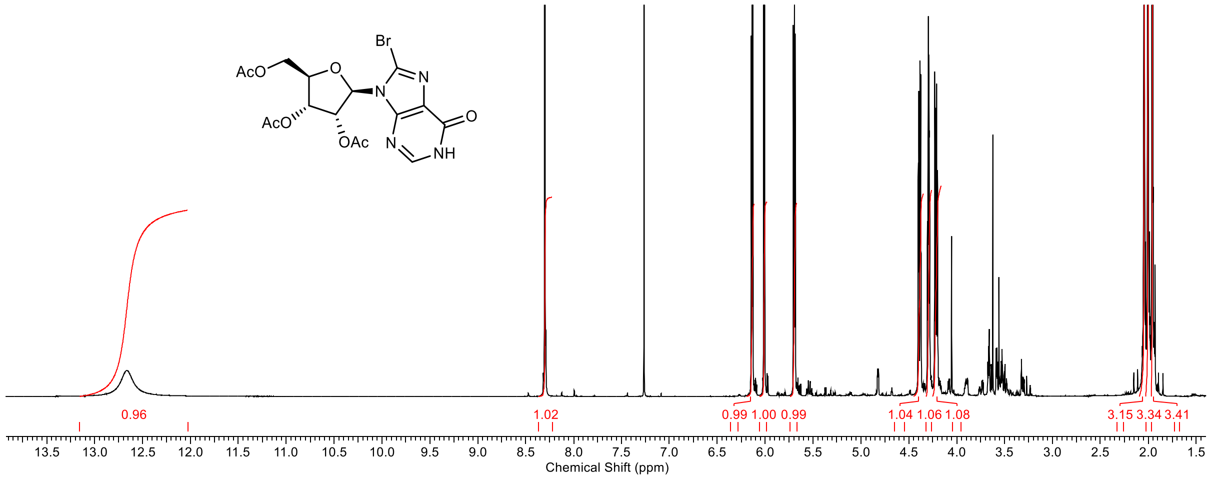

Supplementary Figure 115 | ***^{1}H NMR (600 MHz, {CDCl3}, 1.5-14.0 ppm) spectrum of tri-acetyl-8-bromo-ribo-inosine 18.***

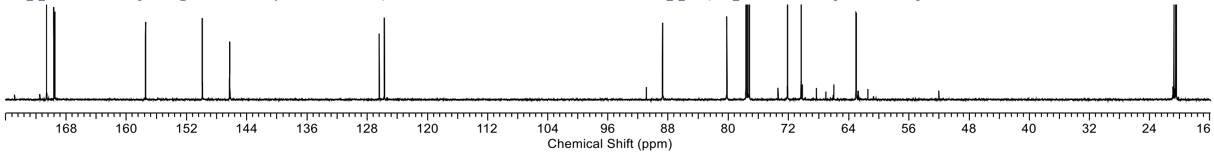

Supplementary Figure 116 | ***^{13}C NMR (151 MHz, {CDCl3}, 16.0–176.0 ppm) spectrum of tri-acetyl-8-bromo-ribo-inosine 18.***

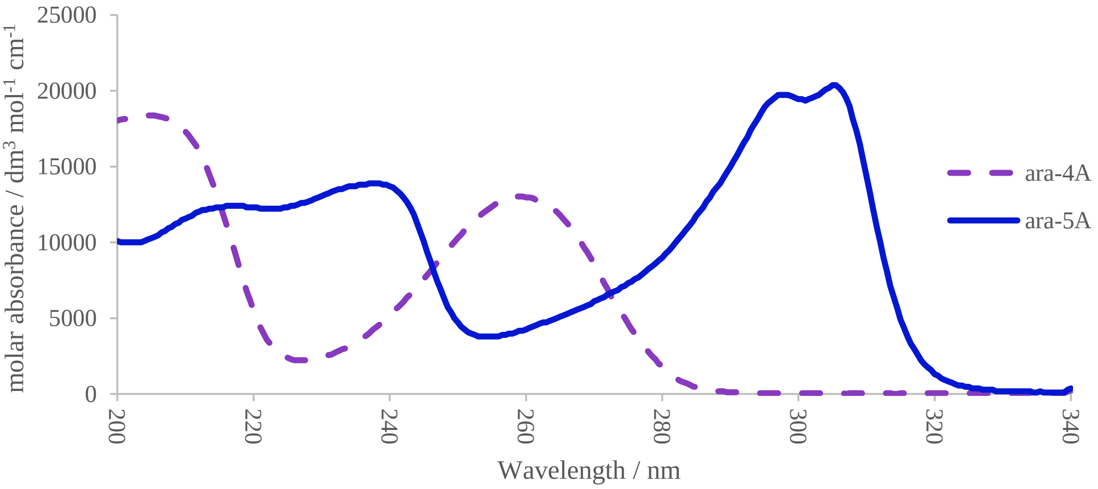

Supplementary Figure 117 | ***UV/Vis spectra (340-200 nm, H_2O) of 8-mercapto-arabino-adenosine ara-5A and arabino-adenosine ara-4A***

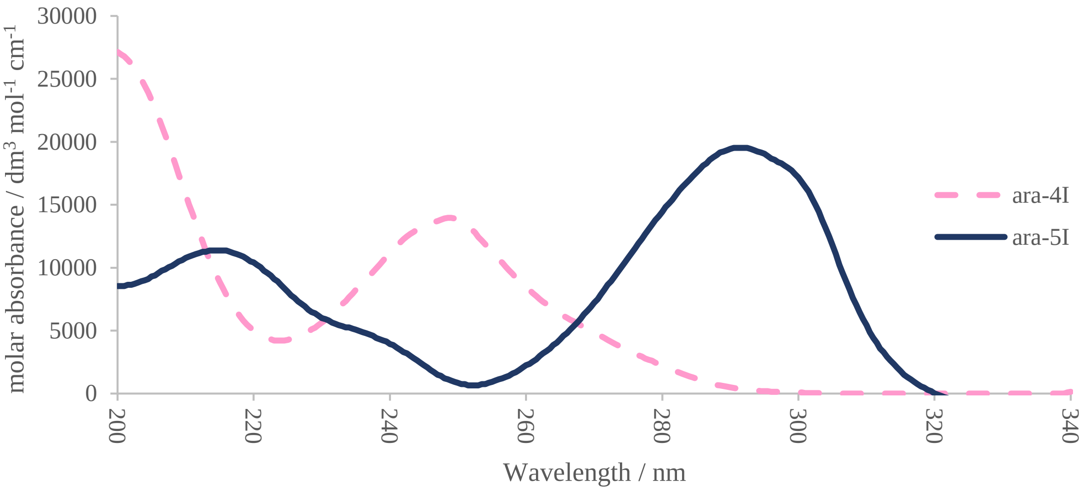

Supplementary Figure 118 | UV/Vis spectra (340-200 nm, H_2O) of 8-mercapto-arabino-inosine ara-5I and arabino-inosine ara-4I

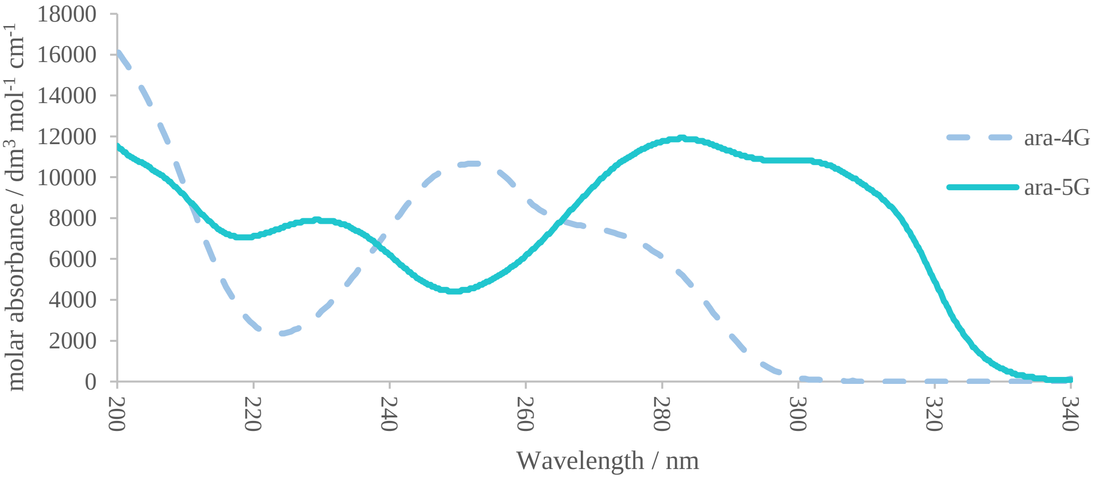

Supplementary Figure 119 | UV/Vis spectra (340-200 nm, H_2O) of 8-mercapto-arabino-guanosine ara-5G and arabino-guanosine ara-4G

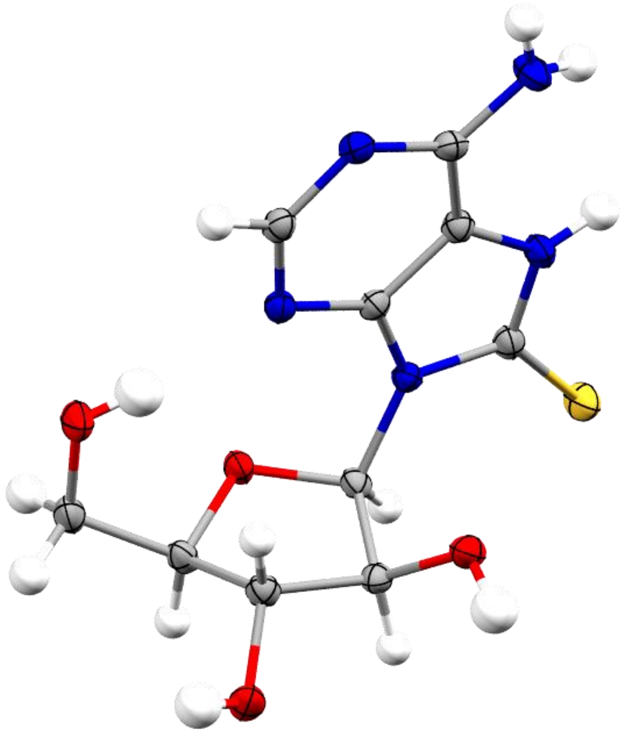

Supplementary Figure 120 | Molecular structure of ara-5A, as obtained by single crystal X-ray diffraction. *The mean atomic- displacement parameters are shown as ellipsoids at the 50% probability level. Colour scheme: carbon = grey, hydrogen = white, nitrogen = blue, oxygen = red and sulfur = yellow.*

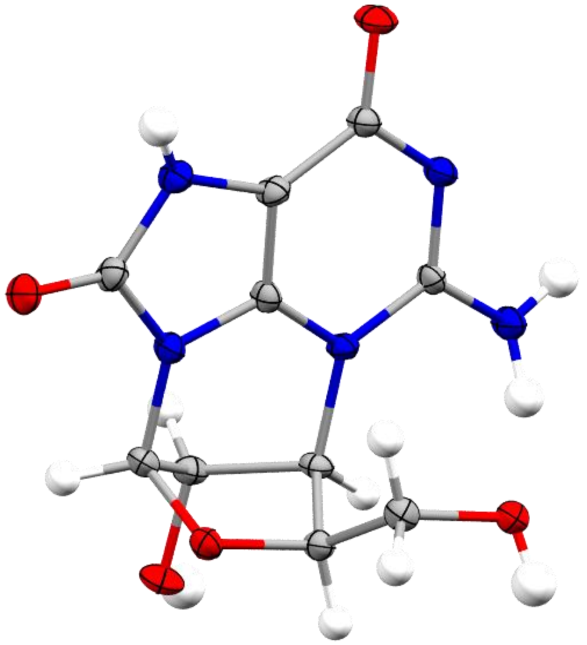

Supplementary Figure 121 | Molecular structure of 12, as obtained by single crystal X-ray diffraction. *The mean atomic-displacement parameters are shown as ellipsoids at the 50% probability level. Colour scheme: carbon = grey, hydrogen = white, nitrogen = blue and oxygen = red.*

Supplementary Tables

Supplementary Table 1 | Vertical excitation energies of ara-5A, ara-5I and ara-5G (C3′-endo-syn conformers).

ADC(2)/cc-pVTZ method				
State	character	f_{osc}	E_{exc} [eV]	λ [nm]
***ara*-5A**				
S_1	$n_S\pi^*$	0.02578185	4.08	303.9
S_2	$\pi\pi_{CS}^*$	0.42364835	4.22	293.8
S_3	$\pi\pi_{ring}^*$	0.06390473	4.85	255.6
S_4	$n_N\pi^*$	0.02466572	5.25	236.2
S_5	$\pi\pi_{ring}^*$	0.06553618	5.34	232.2
S_6	$n_S\pi^*/n_N\pi^*$	0.00061240	5.54	223.8
S_7	$n_S\pi^*/n_N\pi^*$	0.01398965	5.62	220.6
***ara*-5I**				
S_1	$\pi\pi_{ring}^*$	0.08997617	3.94	314.7
S_2	$n_S\pi^*$	0.02153694	4.23	293.1
S_3	$\pi\pi_{CS}^*$	0.40887318	4.40	281.2
S_4	$n_S\pi^*$	0.00347316	4.73	262.1
S_5	$n_O\pi^*$	0.00090187	5.16	240.3
S_6	$\pi\pi_{CS}^*$	0.08394919	5.23	237.1
S_7	$\pi\pi_{ring}^*$	0.13075426	5.57	222.6
***ara*-5G**				
S_1	$\pi\pi_{ring}^*$	0.11109737	4.15	298.8
S_2	$n_S\pi^*$	0.01822744	4.28	289.7
S_3	$\pi\pi_{ring}^*$	0.48557056	4.42	280.5
S_4	$n_S\pi^*$	0.00739863	5.12	242.2
S_5	$n_O\pi^*$	0.00560696	5.28	234.8
S_6	$\pi\pi_{CS}^*$	0.04837175	5.32	233.1
S_7	$\pi\pi_{ring}^*$	0.19315710	5.70	217.5

Supplementary Table 2 | Spin-orbit coupling matrix elements for* ara*-5A and* ara*-5I (C3′-endo-syn conformers). *The square roots of the sum of the sums of squares of the SOCMEs computed at the CASPT2/SA-CASSCF(10,9) level for each considered geometry are presented.*

CASPT2/SA-CASSCF(10,9)/cc-pVTZ-DK		
Initial state	Final state	SOC [cm^{-1}]
***ara*-5A**		
$S_1(\pi\pi_{CS}*)$	$T_1(\pi\pi_{CS}*)$	108.7
$S_1(\pi\pi_{CS}*)$	$T_2(n_S\pi*)$	99.5
$T_1(\pi\pi_{CS}*)$	S_0	99.5
***ara*-5I**		
$S_1(\pi\pi_{CS}*)$	$T_1(\pi\pi_{CS}*)$	102.0
$S_1(\pi\pi_{CS}*)$	$T_2(n_S\pi*)$	73.3
$T_1(\pi\pi_{CS}*)$	S_0	81.4
$T_1(\pi\pi_{ring}*)$	S_0	1.4

Supplementary Table 3 | Crystallographic and refinement parameters for ara-5A and 12.

**compound**	***ara*-5A**	**12**
chemical formula	$C_{10}H_{15}N_5O_5S$	$C_{10}H_{11}N_5O_5$
M_r/gmol^{-1}	317.33	281.24
crystal system	orthorhombic	monoclinic
space group	$P2_12_12_1$	$P2_1$
a/Å	5.09698(3)	7.08510(10)
b/Å	14.89480(10)	9.25750(10)
c/Å	18.0202(2)	8.70750(10)
α/°	90	90
β/°	90	110.1410(10)
γ/°	90	90
V/Å^3	1368.07(2)	536.202(11)
Z	4	2
D_c/gcm^{-3}	1.541	1.742
F(000)	664	292
μ(CuK_α)/mm^{-1}	2.416	1.228
T/K	150.0(1)	150(1)
crystal size/mm	0.39 × 0.07 × 0.02	0.77 × 0.13 × 0.04
index range	−6 → 6<br>−18 → 18<br>−22 → 21	−8 → 8<br>−10 → 10<br>−10 → 10
collected reflections	47093	18462
unique reflections	2768	2070
R_{int}	0.0698	0.0432
reflections with $I > 2\sigma(I)$	2711	2053
no. parameters	205	183
$R(F)$, $F > 2\sigma$(F)	0.0298	0.0279
$wR(F^2)$, $F > 2\sigma$(F)	0.0765	0.0281
$R(F)$, all data	0.0306	0.0772
$wR(F^2)$, all data	0.0774	0.0774
Δ_r (min., max.) eÅ^{-3}	-0.225, 0.291	−0.207, 0.203
CCDC deposition number	1586272	1836032

Supplementary Methods

All compounds were obtained from *Sigma Aldrich, Alfa Aesar, Hopkins and Williams, Fisher Scientific, Carbosynth, BDH, Lancaster and VWR* and used without further purification unless specified. Water (H_2O) refers to deionised water produced by an *Elga Option 3* purification system. Flash Column Chromatography (FCC) and Reverse Phase Flash Column Chromatography (RP-FCC) were carried out using either *Biotage* SNAP or *Kinesis* TELOS cartridges in a *Biotage Isolera One* purification system. After reactions were complete, solutions were sparged of H_2S by bubbling argon or nitrogen gas for 1 h through the solution, whilst maintaining the pH of the solution at pH 2 to ensure efficient degassing and H_2S was quenched with sodium hypochlorite solution. *Bruker* NMR spectrometer *AVANCE III 600* equipped with a *Bruker* 5 mm cryoprobe (600 MHz) was used to provide ^{1}H and ^{13}C NMR data. All reported chemical shifts (δ) are given in parts per million (ppm) relative to residual solvent peaks, and ^{1}H and ^{13}C spectra calibrated using the residual solvent peaks relative shift to TMS. Water supressed ^{1}H NMR spectra were obtained using a 1D nuclear Overhauser enhancement spectroscopy (NOESY) pulse sequence (noesygppr1d, *Bruker*) and all spectra were recorded at 298 K. Rapidly exchanging proton (O–H, N–H) resonances are not detected due to signal broadening and coalescence with the HOD signal in 9:1 H_2O/D_2O. Coupling constants (*J*) are given in Hertz (Hz). The following abbreviations refer to spin multiplicities: s (singlet); d (doublet); t (triplet); q (quartet); m (multiplet); br (broad signal); obs. (obscured/coincidental signals), or any combination of these. Diastereotopic geminal (AB) spin systems coupled to an additional nucleus are reported as ABX. NMR data are stated as follows: chemical shift (number of protons, multiplicity, coupling constants (*J*), nuclear assignment). An electrothermal standard digital apparatus was used to record the melting points for all crystalline solids. Melting points are uncorrected and are quoted to the nearest 0.1 ºC. Infrared spectra (IR) were recorded on a *Shimadzu IR Tracer 100* FT-IR spectrometer. Absorption maxima are reported in wavenumber (cm^{-1}). Ultraviolet (UV) spectra were recorded on a *Shimadzu UV-1800* UV spectrophotometer. Absorption maxima are reported in wavelength (nm). *Mettler Toledo Seven Compact* pH meter with a *Mettler Toledo InLab* semi-micro pH probe and a *Corning pH meter 430* with a *Fischerbrand* (FB68801) semi-micro pH probe were used to measure the pH of solutions. UV irradiations were performed using a *Rayonet RPR-200* with *RPR-3000A* lamps or with *RPR-2547A l*amps or using a *Ultra-Violet Products Ltd (UVP, LLC) Pen-Ray* 254 nm mercury lamp in a water-cooled *Ace Glassware* quartz microreactor. Mass spectra and accurate mass measurements were recorded by *Thermo Finnigan MAT 900XP* or *VG70-SE*, *Waters LCT Premier XE* instruments at the Department of Chemistry, University College London.

Isomerisation of 2′,8-Anhydroinosine ***3I***

2′,8-Anhydroinosine **3I** (10.0 mg, 37.6 µmol) was dissolved in H_2O/D_2O (9:1, 500 µL) and incubated at pH 11 and 40 °C. After 6 and 24 h the pH of the solution was readjusted pH 11 and then NMR spectra were acquired. 2′,8-Anhydroinosine **3I** (84%) and 5′,8-anhydroinosine **10I** (16%) were observed. The pH of the solution was then raised to pH 13 and incubated at 40 °C for 2 h. 2′,8-anhydroinosine **3I** (60%), 5′,8-anhydroinosine **10I** (22%) and 2′,3′-epoxy-8-oxo-inosine **11** (18%) were observed by NMR spectroscopy (Supplementary Figure 1). The observed isomerisations are analogous to those reported for 2′,8-anhydroadenosine (**3A**).[1–3]

*Isomerisation of 2′,8-anhydroguanosine **3G***

2′,8-Anhydroguanosine **3G** (50.0 mg, 177 µmol) was dissolved in NaOD (1 mL, 1 M, D_2O) and the resultant solution was heated at 80 °C for 30 min. The crude mixture was then analysed by NMR spectroscopy. Near quantitative isomerisation to 3′,3-anhydroguanosine **12** was observed (Supplementary Figure 2).

*Thiolysis of 2′,8-anhydroadenosine **3A***

Sodium hydrosulfide (>67% pure, 56.0 mg, 670 µmol) and 2′,8-anhydroadenosine **3A** (6.70 mg, 25.0 µmol) were dissolved in H_2O/D_2O (9:1, 500 µL) and then submitted to *general procedure A* for 2 d, 3 d and 7 d. After 7 d, potassium hydrogen phthalate **13** (5.11 mg, 25.0 µmol) was added as an internal standard. By integration against **13**, 8-mercapto-*arabino*-adenosine ***ara*-5A** was observed to have been furnished in 73% conversion, alongside 5% conversion to 5′,8-anhydroadenosine **10A** (Supplementary Figure 3).

*Thiolysis of 2′,8-anhydroguanosine **3G** with an internal standard*

Disodium sulfide nonahydrate (120 mg, 500 µmol), potassium hydrogen phthalate **13** (0.100 M, 50.0 µL, 5.00 µmol in D_2O) and 2′,8-anhydroguanosine **3G** (7.10 mg, 25.0 µmol) were dissolved in H_2O/D_2O (9:1, 500 µL) and then submitted to *general procedure A* for 7 d. 8-Mercapto-*arabino*-guanosine ***ara*-5G** and 5′,8-anhydroguanosine **10G** were observed be furnished in 83% and 4% conversion, respectively. (Supplementary Figure 4).

*Thiolysis of 2′,8-anhydroinosine **3I** with an internal standard*

Disodium sulfide nonahydrate (120 mg, 500 µmol), potassium hydrogen phthalate **13** (0.100 M, 50.0 µL, 5.00 µmol in D_2O) and 2′,8-anhydroinosine **3I** (6.70 mg, 25.0 µmol) were dissolved in H_2O/D_2O (9:1, 500 µL) and then submitted to *general procedure A* for 7 d. 8-Mercapto-*arabino*-inosine ***ara*-5I** was observed to be furnished in 78% conversion. (Supplementary Figure 5).

*Thiolysis of 2′,8-anhydroadenosine **3A** and 2′,8-anhydroinosine **3I***

2′,8-Anhydroadenosine **3A** (9.50 mg, 36.1 µmol) and 2′,8-anhydroinosine **3I** (9.60 mg, 36.1 µmol) were dissolved in degassed H_2O/D_2O (9:1, 2.00 mL) and analysed by NMR spectroscopy, showing an initial 49:51 ratio of **3A**/**3I**. Sodium hydrosulfide (>67% pure, 161.3 mg, 1.93 mmol) in H_2O/D_2O (9:1, 10.0 mL) was added and then submitted to *general procedure A* for 7 d. 8-Mercapto-*arabino*-adenosine ***ara*-5A**, 8-mercapto-*arabino*-inosine *ara*-5I, 5′,8-anhydroadenosine **10A** and 5′,8-anhydroinosine **10I** were observed in 66%, 75%, 4% and 4% conversion, respectively. (Supplementary Figure 6).

*300 nm UV irradiation of 8-mercapto-arabino-adenosine **ara-5A***

A solution of 8-mercapto-*arabino*-adenosine ***ara*-5A** (2.00 mM, 5.00 mL, 10.0 µmol) was submitted to *general procedure B*. Following 5 h of degassing with a stream of nitrogen and irradiation with 300 nm lamps for 16 h, NMR spectra were acquired of the products showing 66% and 1% conversion to *arabino*-adenosine ***ara*-4A** and 8-mercapto-*arabino*-adenosine ***ara*-5A**, respectively. (Supplementary Figure 7)

*300 nm UV irradiation of 8-mercapto-arabino-adenosine **ara-5A** in an aprotic solvent*

8-Mercapto-*arabino*-adenosine ***ara*-5A** (1.80 mg, 6.00 µmol) was dissolved in anhydrous DMF (3.00 mL) and degassed with a stream of nitrogen for 3 h. The solution was irradiated with 300 nm lamps for 16 h and concentrated *in vacuo*. After dissolving in D_2O, NMR spectra were acquired. A solution of potassium hydrogen phthalate **13** (0.100 M, 50.0 µL, 5.00 µmol in D_2O) was added as an internal NMR

standard, and NMR spectra were then reacquired. NMR spectra acquired of the products showed a 36% conversion to *arabino*-adenosine ***ara*-4A**. (Supplementary Figure 8).

254 nm UV irradiation of 8-mercapto-arabino-adenosine ***ara*-5A**

8-Mercapto-*arabino*-adenosine ***ara*-5A** (2.99 mg, 10.0 µmol) dissolved H_2O (5.00 mL) was adjusted to pH 6.5. The solution was transferred to an *Ace Glassware quartz UV microreactor assembly* and degassed with a constant stream of argon for 5 h. The solution was irradiated at 254 nm (with a *UVP, LLC* 11SC-2.12 mercury *Pen-Ray Lamp*) for 16 h whilst a constant stream of argon was bubbled through the solution at room temperature. After irradiation, argon was bubbled through the solution for a further 2 h. A small amount of H_2O (~250 µL) evaporated during the irradiation. The irradiated solution was lyophilised. The lyophilisate was dissolved in D_2O (1 mL) and NMR spectra were acquired. A solution of potassium hydrogen phthalate **13** (0.100 M, 50.0 µL, 5.00 µmol in D_2O) was added and NMR spectra were then acquired. Analysis of the NMR spectra showed a 70% conversion to *arabino*-adenosine ***ara*-4A** and 5% residual 8-mercapto-*arabino*-adenosine ***ara*-5A**. (Supplementary Figure 9).

300 nm UV irradiation of 8-mercapto-arabino-inosine ***ara*-5I**

A solution of 8-mercapto-*arabino*-inosine ***ara*-5I** (2.00 mM, 3.00 mL, 6.00 µmol) was submitted to *general procedure B* with 300 nm lamps for 16 h. 2′,8-Anhydroinosine **3I** (2%) and *arabino*-inosine ***ara*-4I** (15%) were observed. The identity of 2′,8-anhydroinosine **3I** and *arabino*-inosine ***ara*-4I** were confirmed by spiking the NMR sample with authentic samples and observing increased peak intensities. (Supplementary Figure 10).

2 step synthesis of arabino-guanosine ***ara*-4G** *from 2′,8-anhydroguanosine* **3G**

2′,8-Anhydroguanosine **3G** (20.2 mg, 72.0 µmol), sodium hydrosulfide (>67% pure, 161 mg, 1.93 mmol) were dissolved in H_2O/D_2O (9:1, 12.0 mL) and then submitted to *general procedure A*. NMR spectra of the solution were acquired after 7 d showing residual starting material 2′,8-anhydroguanosine **3G**, and 8-mercapto-*arabino*-guanosine ***ara*-5G** and 5′,8-anhydroguanosine **10G** were present in 3:78:19 ration of **3G**/***ara*-5G**/**10G**. The solution was then submitted to *general procedure B* with 300 nm lamps for 22 h. Analysis of the resultant solution by NMR spectroscopy showed that 2′,8-anhydroguanosine **3G** (2%), *arabino*-guanosine ***ara*-4G** (59%) and 5′,8-anhydroguanosine **10G** (4%) were furnished, after the 2 steps. The identity of 2′,8-anhydroguanosine **3G** and *arabino*-guanosine ***ara*-4G** were confirmed by spiking the NMR sample authentic samples of **3G** and ***ara*-4G** and observing increased peak intensities. (Supplementary Figure 11).

UV irradiation of 8-mercapto-arabino-inosine ***ara*-5I** *and 8-mercapto-arabino-adenosine* ***ara*-5A**

A solution of 8-mercapto-*arabino*-inosine ***ara*-5I** (1.50 mg, 5.00 µmol) and 8-mercapto-*arabino*-adenosine ***ara*-5A** (1.50 mg, 5.00 µmol) dissolved in H_2O/D_2O (9:1, 5.00 mL) were analysed by NMR an observed in an initial 47:53 ratio of ***ara*-5I**/***ara*-5A**. This mixture was then submitted to *general procedure B* with 300 nm lamps for 16 h. Analysis of the resultant solution by NMR spectra showed *arabino*-inosine ***ara*-4I** (13%), *arabino*-adenosine ***ara*-4A** (62%) 8-mercapto-*arabino*-inosine ***ara*-5I** (4%) and 8-mercapto-*arabino*-adenosine ***ara*-5A** (7%) had been furnished. The identity of *arabino*-adenosine ***ara*-4A** and *arabino*-inosine ***ara*-4I** were confirmed by spiking the NMR sample with authentic samples of ***ara*-4A** and ***ara*-4I**, and observing increased peak intensities. (Supplementary Figure 12).

*UV irradiation of arabino-inosine **ara-4I** and arabino-adenosine **ara-4A***

arabino-Inosine ***ara*-4I** (0.90 mg, 3.00 µmol) and *arabino*-adenosine ***ara*-4A** (0.90 mg, 3.00 µmol) were dissolved in H_2O/D_2O (9:1, 3.00 mL) and NMR spectra were acquired showing an initial 1:1 ratio of ***ara*-4I**/***ara*-4A**. This mixture was submitted to *general procedure B* with either 254 nm or 300 nm lamps for 16 h. Following irradiation, analysis by NMR showed that conversion to *arabino*-inosine ***ara*-4I** and *arabino*-adenosine ***ara*-4A** had occurred, with 86% and 93% conversion after irradiation at 254 nm and with 73% and 73% conversion after irradiation at 300 nm, respectively. (Supplementary Figure 13).

*UV irradiation of 8-mercapto-arabino-adenosine **ara-5A** and 8-mercapto-ribo-adenosine **ribo-5A***

A solution of 8-mercapto-*arabino*-adenosine ***ara*-5A** (1.80 mg, 6.00 µmol) and 8-mercapto-*ribo*-adenosine ***ribo*-5A** (1.80 mg, 6.00 µmol) dissolved in H_2O/D_2O (9:1, 6.00 mL) was analysed by NMR showing an initial 47:53 ratio of ***ara*-5A**/***ribo*-5A**. This mixture was submitted to *general procedure B* with 300 nm lamps for 16 h. Following irradiation, analysis of the solution by NMR spectroscopy showed conversion to *arabino*-adenosine ***ara*-4A** (68%), *ribo*-adenosine ***ribo*-4A** (70%) alongside residual 8-mercapto-*arabino*-adenosine ***ara*-5A** (23%) and 8-mercapto-*ribo*-adenosine ***ribo*-5A** (18%). The identity of *arabino*-adenosine ***ara*-4A** and *ribo*-adenosine ***ribo*-4A** were confirmed by spiking the NMR sample with authentic samples of ***ara*-4A** and ***ribo*-4A** and observing increased peak intensities. (Supplementary Figure 14).

*UV irradiation of 8-mercapto-ribo-guanosine **ribo-5G** and 8-mercapto-ribo-adenosine **ribo-5A***

A solution of 8-mercapto-*ribo*-guanosine ***ribo*-5G** (1.90 mg, 6.00 µmol) and 8-mercapto-*ribo*-adenosine ***ribo*-5A** (1.80 mg, 6.00 µmol) dissolved in H_2O/D_2O (9:1, 6.00 mL) was analysed by NMR showing an initial 1:1 ratio of ***ribo*-5G**/***ribo*-5A**. This mixture was submitted to *general procedure B* with 300 nm lamps for 16 h. Analysis of the resultant solution by NMR spectroscopy showed conversion to *ribo*-guanosine ***ribo*-4G**, *ribo*-adenosine ***ribo*-4A**, 8-mercapto-*ribo*-guanosine ***ribo*-5G** and 8-mercapto-*ribo*-adenosine ***ribo*-5A** (80%, 51%, 5% and 12%, respectively). The identity of *ribo*-adenosine ***ribo*-4A** and *ribo*-guanosine ***ribo*-4G** were confirmed by spiking the NMR sample with authentic samples of ***ribo*-4A** and ***ribo*-4G** and observing increased peak intensities. (Supplementary Figure 15).

*UV irradiation of 8-mercapto-ribo-inosine **ribo-5I** and 8-mercapto-ribo-adenosine **ribo-5A***

A solution of 8-mercapto-ribo-inosine ***ribo*-5I** (1.80 mg, 6.00 µmol) and 8-mercapto-ribo-adenosine ***ribo*-5A** (1.80 mg, 6.00 µmol) dissolved in H_2O/D_2O (9:1, 6.00 mL) was analysed by NMR showing an initial 48:52 ratio of ***ribo*-5I**/***ribo*-5A**. This mixture was submitted to general procedure B with 300 nm lamps for 16 h. Analysis of the resultant solution by NMR spectroscopy showed conversion to ribo-inosine ***ribo*-4I**, ribo-adenosine ***ribo*-4A**, 8-mercapto-ribo-inosine ***ribo*-5I** and 8-mercapto-ribo-adenosine ***ribo*-5A** (10%, 55%, 32% and 22%, respectively). The identity of ribo-adenosine ***ribo*-4A** and ribo-inosine ***ribo*-4I** were confirmed by spiking the NMR sample with authentic samples of ***ribo*-4A** and ***ribo*-4I** and observing increased peak intensities. (Supplementary Figure 16).

*Oxidation of 8-mercapto-arabino-adenosine **ara-5A** in acidic methanol solution.*[2]

8-Mercapto-*arabino*-adenosine ***ara*-5A** (15.0 mg, 50.0 µmol) was dissolved in MeOH/H_2O (1:1, 1 mL). Aqueous HCl (0.1 M, 50.0 µL) was added to the solution followed by hydrogen peroxide (3% w/w solution in H_2O, 0.200 mL, 196 µmol). The solution was left to stand overnight at room temperature. The solution was neutralized with NaOH solution (0.1 M) and concentrated *in vacuo.* The crude material was dissolved in D_2O (0.500 mL) and NMR spectra were acquired. A solution of potassium hydrogen phthalate **13** (0.1 M, 100 µL, 10 µmol in D_2O) was added as an internal NMR standard and NMR spectra

were then reacquired. *arabino*-Adenosine ***ara*-4A** (50%) and 2′,8-anhydroadenosine **3A (**4%) were observed. (Supplementary Figure 17).

*8-Mercapto-arabino-adenosine **ara-5A** oxidation with hydrogen peroxide*
8-Mercapto-*arabino*-adenosine ***ara*-5A** (7.50 mg, 25.0 µmol) in H_2O/D_2O (9:1, 450 µL) was submitted to *general procedure C* with H_2O_2 (30% w/w, 7.50 µL, 75.0 µmol) for 3 h. Analysis of the resultant solution by NMR spectra showed that *arabino*-adenosine ***ara*-4A** (85%) had been formed. The identity of *arabino*-adenosine ***ara*-4A** was confirmed by spiking the NMR sample with a commercial sample of ***ara*-4A** and observing increased peak intensities. (Supplementary Figure 18).

*8-Mercapto-arabino-inosine **ara-5I** oxidation with hydrogen peroxide*
8-Mercapto-*arabino*-inosine ***ara*-5I** (7.50 mg, 25.0 µmol) in H_2O/D_2O (9:1, 450 µL) was submitted to *general procedure C* with H_2O_2 (30% w/w, 7.50 µL, 75.0 µmol) for 1 h. Analysis of the resultant solution by NMR spectroscopy indicated that *arabino*-inosine ***ara*-4I** (90%) and 2′,8-anhydroinosine **3I** (2%) had been formed. The identity of *arabino*-inosine ***ara*-4I** was confirmed by spiking the NMR sample with an authentic sample of ***ara*-4I** and observing increased peak intensities. (Supplementary Figure 19).

*8-Mercapto-arabino-adenosine **ara-5A** and 8-mercapto-arabino-inosine **ara-5I** oxidation*
8-Mercapto-*arabino*-inosine ***ara*-5I** (7.50 mg, 25.0 µmol) and 8-mercapto-*arabino*-adenosine ***ara*-5A** (7.50 mg, 25.0 µmol) in H_2O/D_2O (9:1, 900 µL) were analysed by NMR an observed in an initial 48:52 ratio of ***ara*-5I**/***ara*-5A**. The solution was submitted to *general procedure C* with H_2O_2 (30% w/w, 15.0 µL, 150 µmol) for 1 h. Analysis of the resultant solution by NMR spectroscopy showed *arabino*-adenosine ***ara*-4A** (88%) and *arabino*-inosine ***ara*-4I** (91%) had been furnished. The identity of *arabino*-adenosine ***ara*-4A** and *arabino*-inosine ***ara*-4I** were confirmed by spiking the NMR sample with authentic samples of ***ara*-4A** and ***ara*-4I** and observing increased peak intensities. (Supplementary Figure 20).

*2 step synthesis of arabino-guanosine **ara-4G** from 2′,8-anhydroguanosine **3G***
2′,8-Anhydroguanosine **3G** (7.10 mg, 25.0 µmol), disodium sulfide nonahydrate (120 mg, 500 µmol) and potassium hydrogen phthalate **13** (0.100 M, 50.0 µL, 5.00 µmol in D_2O) were dissolved in H_2O/D_2O (9:1, 450 µL) submitted to *general procedure A*. After 7 d NMR spectra were acquired, and 8-mercapto-*arabino*-guanosine ***ara*-5G** (90%) was observed. The solutions was submitted to *general procedure C* with H_2O_2 (30% w/w, 39.0 µL, 390 µmol) for 1 h. NMR spectra were then reacquired, which showed *arabino*-guanosine ***ara*-4G** (89%) had been formed in 2 steps. The presence *arabino*-guanosine ***ara*-4G** was confirmed by spiking the NMR sample with a commercial sample of ***ara*-4G** and observing increased peak intensities. (Supplementary Figure 21).

*Hydrolysis of arabino-2′,2-anhydrocytidine **3C** in the presence of 2′,8-anhydroadenosine **3A***
arabino-2′,2-Anhydrocytidine **3C** (6.30 mg, 25.0 µmol) and 2′,8-anhydroadenosine **3A** (6.70 mg, 25.0 µmol) were dissolved in phosphate buffer (500 mM, 2.00 mL, in H_2O/D_2O, 9:1), the pH of the solution was adjusted to pH 8. NMR spectra were taken and an initial 54:46 ratio of **3C**/**3A** was observed. NMR spectra were periodically acquired and hydrolysis of **3C** to ***ara*-4C** was observed to be complete after 6 h. After 24 h, potassium hydrogen phthalate **13** (0.10 M, 50.0 µL, 5.00 µmol in D_2O) was added, and NMR spectra were then reacquired. *arabino*-Cytidine ***ara*-4C** and 2′,8-anhydroadenosine **3A** were observed to have been furnished quantitatively. The identity of 2′,8-anhydroadenosine **3A** and *arabino*-

cytidine ***ara*-4C** were confirmed by spiking the NMR sample with authentic samples of **3A** and ***ara*-4C** and observing increased peak intensities. (Supplementary Figure 22).

*2′,2-Anhydrocytidine **3C** hydrolysis in the presence of hydrogen sulfide at room temperature*
2′,2-Anhydrocytidine **3C** (13.1 mg, 50.0 µmol) and sodium hydrosulfide (84 mg, 1.00 mmol) were dissolved in H_2O/D_2O (9:1, 1.60 mL). The pH of the solution was measured and observed to be pH 10.7. After 2 d, the pH of the solution had risen to pH 9.6 and NMR spectra were acquired. *arabino*-Cytidine ***ara*-4C** (91%), 2-thio-*arabino*-cytidine ***ara*-6C** (8%) were observed. (Supplementary Figure 23).

*Thiolysis of 2′,2-anhydrocytidine **3C** in formamide* [5]
2′,2-Anhydrocytidine **3C** (10.0 mg, 38.0 µmol) and sodium hydrogen sulfide (14.0 mg, 153 µmol) were dissolved in formamide (1.00 mL) and heated at 50 °C for 7 h. The solution was diluted with D_2O (10.0 mL) and lyophilised three times. The resultant lyophilisate was then dissolved in D_2O (0.500 mL) and NMR spectra were acquired. Potassium hydrogen phthalate **13** (0.100 M, 50.0 µL, 5.00 µmol in D_2O) was added and further NMR spectra were acquired. Analysis of the NMR spectra showed formation of 2-thio-*arabino*-cytidine ***ara*-6C** (31%) and *arabino*-cytidine ***ara*-4C** (24%), with 22% retention of *arabino*-2′,2-anhydrocytidine **3C** starting material. (Supplementary Figure 24)

*Thiolysis of 2′,2-anhydrocytidine **3C***
2′,2-Anhydrocytidine **3C** (13.1 mg, 50.0 µmol) and disodium sulfide nonahydrate (240 mg, 1.00 mmol) were dissolved in H_2O/D_2O (9:1, 1.60 mL) and submitted to *general procedure A* for a specified time: **a)** After 2 d, NMR spectra were acquired. Potassium hydrogen phthalate **13** (0.10 M, 50.0 µL, 5.00 µmol in D_2O) was added and NMR spectra were reacquired. *arabino*-Cytidine ***ara*-4C** (68%), 4-thio-*arabino*-uridine ***ara*-7U** (22%) and 2-thio-*arabino*-cytidine ***ara*-6C** (4%) were observed. (Supplementary Figure 25). **b)** After 7 d, the solution was lyophilised, the lyophilisate dissolved in D_2O and NMR spectra were acquired. Potassium hydrogen phthalate **13** (0.10 M, 50.0 µL, 5.0 µmol in D_2O) was added and NMR spectra were then reacquired. *arabino*-Cytidine ***ara*-4C** (57%), 4-thio-*arabino*-uridine ***ara*-7U** (38%) and 2-thio-*arabino*-cytidine ***ara*-6C** (3%) were observed. The identity of *arabino*-cytidine ***ara*-6C** was confirmed by spiking the NMR sample with an authentic commercial sample and observing increased peak intensities. (Supplementary Figure 25)

*Thiolysis of ribo-2′,2-anhydrocytidine **α-ribo-3C***
ribo-2′,2-Anhydrocytidine **α-*ribo*-3C** (13.1 mg, 50.0 µmol) and disodium sulfide nonahydrate (240 mg, 1.00 mmol) were dissolved in H_2O/D_2O (9:1, 1.60 mL) and submitted to *general procedure A* for 7 d. After 7 d, the solution was lyophilised. The lyophilisate was then dissolved in D_2O and NMR spectra were acquired. Potassium hydrogen phthalate **13** (0.10 M, 50.0 µL, 5.0 µmol in D_2O) was added to the solution and NMR spectra were then reacquired. α-*ribo*-Cytidine **α-*ribo*-4C** (52%) and 4-thio-α-*ribo*-uridine **α-*ribo*-7U** (39%) were observed. The identity of α-*ribo*-cytidine **α-*ribo*-4C** was confirmed by spiking the NMR sample with an authentic sample and observing increased peak intensities. (Supplementary Figure 26).

*Simultaneous thiolysis of 2′,2-anhydrocytidine **3C** and 2′,8-anhydroadenosine **3A***
2′,2-Anhydrocytidine **3C** (13.0 mg, 50.0 µmol) and 2′,8-anhydroadenosine **3A** (13.2 mg, 50.0 µmol) were dissolved in H_2O/D_2O (9:1, 1.20 mL) and NMR spectra were acquired. Disodium sulfide nonahydrate (480 mg, 2.00 mmol) was added to the solution and it was then submitted to *general*

procedure A for 7 d. After 7 d the solution was lyophilised. The lyophilisate was dissolved in D_2O and NMR spectra were acquired. Potassium hydrogen phthalate **13** (0.100 M, 50.0 µL, 5.00 µmol in D_2O) was added and further NMR spectra were acquired. Analysis of the NMR spectra showed 8-mercapto-*arabino*-adenosine ***ara*-5A** (71%), *arabino*-cytidine ***ara*-4C** (70%) and 4-thio-*arabino*-uridine ***ara*-7U** (30%) were furnished. The identity of *arabino*-cytidine ***ara*-4C** was confirmed by spiking the NMR sample with an authentic sample and observing increased peak intensities. (Supplementary Figure 27).

Thiolysis of arabino-cytidine ***ara-4C***

arabino-Cytidine ***ara*-4C** (12.2 mg, 50.2 µmol) and disodium sulfide nonahydrate (240 mg, 1.00 mmol) were dissolved in H_2O/D_2O (9:1, 0.500 mL) and then submitted to *general procedure A*. After 7 d, potassium hydrogen phthalate **13** (0.100 M, 50.0 µL, 5.00 µmol in D_2O) was added and further NMR spectra were acquired. *arabino*-Cytidine ***ara*-4C** (52%) and 4-thio-*arabino*-uridine ***ara*-7U** (40%) were observed. The identity of *arabino*-cytidine ***ara*-4C** was confirmed by spiking the NMR sample with a commercial sample and observing increased peak intensities. (Supplementary Figure 28).

Thiolysis of β-ribo-cytidine ***β-ribo-4C***

Disodium sulfide nonahydrate (480 mg, 2.00 mmol), H_2O/D_2O (2.00 mL, 9:1) and β-*ribo*-cytidine **β-*ribo*-4C** (24.4 mg, 0.100 mmol) were submitted to *general procedure A* for 7 d and then NMR spectra of the resultant solution were acquired. *ribo*-Cytidine **β-*ribo*-4C** (84%) and 4-thio-*ribo*-uridine **β-*ribo*-7U** (16%) were observed. (Supplementary Figure 29).

Thiolysis of α-ribo-cytidine ***α-ribo-4C***

Disodium sulfide nonahydrate (480 mg, 2.00 mmol), H_2O/D_2O (2.00 mL, 9:1) and α-*ribo*-cytidine **α-*ribo*-4C** (24.4 mg, 0.100 mmol) were submitted to *general procedure A* for 8 d and then NMR spectra of the resultant solution were acquired. α-*ribo*-Cytidine **α-*ribo*-4C** (37%) and 4-thio-α-*ribo*-uridine **α-*ribo*-7U** (63%) were observed. (Supplementary Figure 30).

Simultaneous thiolysis of ribo-cytidine ***β-ribo-4C*** *and arabino-cytidine* ***ara-4C***

Disodium sulfide nonahydrate (480 mg, 2.00 mmol), H_2O/D_2O (1.00 mL, 9:1), *ribo*-cytidine **β-*ribo*-4C** (12.2 mg, 0.100 mmol) and *arabino*-cytidine ***ara*-4C** (12.2 mg, 0.100 mmol) were submitted to *general procedure A* for 7 d and then NMR spectra of the resultant solution were acquired. *ribo*-Cytidine **β-*ribo*-4C** (68%), *arabino*-cytidine ***ara*-4C** (41%), 4-thio-*ribo*-uridine **β-*ribo*-7U** (18%) and 4-thio-*arabino*-uridine ***ara*-7U** (52%) were observed. The identity of **β-*ribo*-4C**, ***ara*-4C**, **β-*ribo*-7U** and ***ara*-7U** were confirmed by spiking the NMR sample with authentic samples and observing increased peak intensities. (Supplementary Figure 31).

254 nm irradiation of arabino-cytidine ***ara-4C***

The photochemistry of **α-*ribo*-4C** and ***ara*-4C** has been studied extensively and the photochemical hydrolysis of cytidines to uridines reported.[4-6] Irradiation of ***ara*-4C** (2.00 mM, pH 6.5) formed ***ara*-4U** (14% at λ=254 nm; 20% at λ=300 nm; Supplementary Fig. 32 + 33). Irradiation of ***ara*-7U** and photohydrate relaxation furnished ***ara*-4U** (8% at λ=254 nm; 4% at λ=300 nm). Little (6%) or no residual starting material at λ=254 or 300 nm, respectively (Supplementary Fig. 35 + 36). ***ara*-4U** can be accessed by UV irradiation of ***ara*-4C** or ***ara*-7U**, but these irradiations are destructive due to nucleobase loss and oxazolidinone formation.[4-6] *arabino*-Cytidine ***ara*-4C** (1.41 mg, 6.00 µmol) was dissolved in H_2O/D_2O (9:1, 3.00 mL) and then submitted to *general procedure B* with 254 nm lamps for 16 h. Upon analysis of the resultant solution by NMR spectroscopy *arabino*-cytidine ***ara*-4C** (20%),

arabino-uridine ***ara*-4U** (14%), uracil **14** (2%), cytosine **15** (3%) and *arabino*-oxazolidinone **16** (17%) were observed, alongside a mixture of α-pyrimidines (3%). The identity of *arabino*-cytidine ***ara*-4C**, *arabino*-uridine ***ara*-4U**, uracil **14**, cytosine **15** and *arabino*-oxazolidinone **8** were confirmed by spiking the NMR sample with authentic samples of ***ara*-4U**, ***ara*-4C**, **8**, **14** and **15**, and observing increased peak intensities. (Supplementary Figure 32).

300 nm irradiation of arabino-cytidine ***ara-4C***

arabino-Cytidine ***ara*-4C** (2.92 mg, 12.0 µmol) was dissolved in H_2O/D_2O (9:1, 6.00 mL) and then submitted to *general procedure B* with 300 nm lamps for 16 h. Pyrimidine photo-hydrates were relaxation at 90 °C for 16 h.[4] Upon analysis of the resultant solution by NMR spectroscopy *arabino*-uridine ***ara*-4U** (20%), *arabino*-cytidine ***ara*-4C** (15%), uracil **14** (3%), cytosine **15** (4%), and *arabino*-oxazolidinone **16** (17%) were observed. The identity of *arabino*-uridine ***ara*-4U**, *arabino*-cytidine ***ara*-4C**, uracil **14**, cytosine **15** and *arabino*-oxazolidinone **8** were confirmed by spiking the NMR sample with authentic samples of ***ara*-4U**, ***ara*-4C**, **8**, **14** and **15**, and observing increased peak intensities. (Supplementary Figure 33).

Irradiation of 8-mercapto-arabino-adenosine ***ara-5A*** *and arabino-cytidine* ***ara-4C***

8-Mercapto-*arabino*-adenosine ***ara*-5A** (1.79 mg, 6.00 µmol) and *arabino*-cytidine ***ara*-4C** (1.41 mg, 6.00 µmol) were dissolved in H_2O/D_2O (9:1, 6.00 mL) and NMR spectra were acquired showing an initial 1:1 ratio of ***ara*-5A/*ara*-4C**. The mixture was then submitted to *general procedure B* with 254 nm lamps for 3 d. Pyrimidine photo-hydrates were relaxed at 90 °C for 16 h.[4] Upon analysis of the resultant solution by NMR spectroscopy *arabino*-adenosine ***ara*-4A** (40%), *arabino*-cytidine ***ara*-4C** (23%) and *arabino*-uridine ***ara*-4U** (13%) were observed. The identity of *arabino*-adenosine ***ara*-4A**, *arabino* cytidine ***ara*-4C** and *arabino*-uridine ***ara*-4U** were confirmed by spiking the NMR sample with authentic samples of ***ara*-4A**, ***ara*-4C** and ***ara*-4U** and observing increased peak intensities. (Supplementary Figure 34).

254 nm irradiation of 4-thio-arabino-uridine ***ara-7U***

A solution of 4-thio-*arabino*-uridine ***ara*-7U** (1.52 mM, 2.28 mL, 4.56 µmol, in H_2O) was submitted to *general procedure B* with 254 nm lamps for 16 h. Pyrimidine photo-hydrates were relaxed at 90 °C for 16 h.[4] Upon analysis of the resultant solution by NMR spectroscopy *arabino*-oxazolidinone **8** (4%) and *arabino*-uridine ***ara*-4U** (8%) were observed, whilst 6% of the starting material 4-thio-*arabino*-uridine **ara-7U** was returned. The identity of *arabino*-uridine ***ara*-4U** was confirmed by spiking the NMR sample with a commercial sample of ***ara*-4U** and observing increased peak intensities. (Supplementary Figure 35).

300 nm irradiation of 4-thio-arabino-uridine ***ara-7U***

A solution of 4-thio-*arabino*-uridine ***ara*-7U** (2.00 mM, 3.00 mL, 6.00 µmol, in H_2O) was submitted to *general procedure B* with 300 nm lamps for 16 h. Potassium hydrogen phthalate **13** solution (50 µL, 0.100 M, in D_2O) was added and pyrimidine photo-hydrates were relaxed at 90 °C for 16 h.[4] Upon analysis of the resultant solution by NMR spectroscopy *arabino*-oxazolidinone **8** (4%) and *arabino*-uridine ***ara*-4U** (4%) were observed. (Supplementary Figure 36).

Control for photorelaxation of 4-thio-arabino-uridine ***ara-7U***

4-Thio-*arabino*-uridine ***ara*-7U** (1.27 mg, 4.89 µmol) was dissolved in H_2O (11.00 mL). The solution was stirred overnight at 90 °C and then the solution was lyophilised. The lyophilisate was dissolved in

D_2O (500 µL) and NMR spectra acquired. A solution of potassium hydrogen phthalate **13** (0.100 M, 50.0 µL, 5.00 µmol in D_2O) was added as an internal NMR standard and NMR spectra were acquired. Analysis of the NMR spectra showed *arabino*-uridine ***ara*-4U** (8%) was formed, whilst 85% 4-thio-*arabino*-uridine ***ara*-7U** was returned. The identity of *arabino*-uridine ***ara*-4U** was confirmed by spiking the NMR sample with a commercial sample and observing increased peak intensities. (Supplementary Figure 37).

Oxidation of 4-thio-arabino-uridine ***ara-7U***
4-Thio-*arabino*-uridine ***ara*-7U** (6.50 mg, 25.0 µmol) in H_2O/D_2O (9:1, 450 µL) was submitted to *general procedure C* with H_2O_2 (30% w/w, 7.50 µL, 75.00 µmol) for 2.5 h. Analysis of the resultant solution by NMR spectroscopy showed *arabino*-uridine ***ara*-4U** (78%) had formed. The identity of *arabino*-uridine ***ara*-4U** was confirmed by spiking the NMR sample with a commercial sample of ***ara*-4U** and observing increased peak intensities. (Supplementary Figure 38).

2 Step synthesis of arabino-cytidine ***ara-4C*** *and arabino-uridine* ***ara-4U***
2′,2-Anhydrocytidine **3C** (6.54 mg, 25.0 µmol), disodium sulfide nonahydrate (120 mg, 500 µmol) and potassium hydrogen phthalate **13** (0.100 M, 50.0 µL, 5.00 µmol in D_2O) were dissolved in H_2O/D_2O (9:1, 450 µL). The mixture was submitted to the *general procedure A* for 7 d. The resultant solution was the analysed by NMR spectroscopy which showed *arabino*-cytidine ***ara*-4C** (64%) and 4-thio-*arabino*-uridine ***ara*-7U** (27%) had formed. The reaction mixture was submitted to *general procedure C* with H_2O_2 (30% w/w, 39.0 µL, 375.00 µmol) for 1 h. Analysis of the resultant solution by NMR spectroscopy showed *arabino*-cytidine ***ara*-4C** (62%) and *arabino*-uridine ***ara*-4U** (25%) had been furnished, over 2 steps. The identity of *arabino*-cytidine ***ara*-4C** and *arabino*-uridine ***ara*-4U** were confirmed by spiking the NMR sample with commercial samples of ***ara*-4C** and ***ara*-4U** and observing increased peak intensities. (Supplementary Figure 39).

Oxidiation of 4-thio-ribo-uridine **β-*ribo*-7U**
4-Thio-*ribo*-uridine **β-*ribo*-7U** (7.40 mg, 28.4 µmol) in H_2O/D_2O (9:1, 519 µL) was submitted to *general procedure C* with H_2O_2 (30% w/w, 8.5 µL, 85.0 µmol) for 1 h. Analysis of the resultant solution by NMR spectroscopy showed *ribo*-uridine **β-*ribo*-4U** (78%) had formed. The identity of *ribo*-uridine **β-*ribo*-4U** was confirmed by spiking the NMR sample with a commercial sample of **β-*ribo*-4U** and observing increased peak intensities. (Supplementary Figure 40).

Oxidiation of 4-thio-α-ribo-uridine ***α-ribo-7U***
4-Thio-α-*ribo*-uridine **α-*ribo*-7U** (9.60 mg, 36.9 µmol) in H_2O/D_2O (9:1, 688 µL) was submitted to *general procedure C* with H_2O_2 (11.1 µL, 111 µmol) for 3 h. Analysis of the resultant solution by NMR spectroscopy showed α-*ribo*-uridine **α-*ribo*-4U** (93%) had formed. (Supplementary Figure 41).

Oxidiation of 2-thio-arabino-cytidine ***ara*-6C**
2-Thio-*arabino*-cytidine ***ara*-6C** (6.48 mg, 25.0 µmol) in H_2O/D_2O (9:1, 450 µL) was submitted to *general procedure C* with H_2O_2 (30% w/w, 7.50 µL, 75.0 µmol) for 1 h. Analysis of the resultant solution by NMR spectroscopy showed *arabino*-2′,2-anhydrocytidine **3C** (82%) had formed. The identity of *arabino*-2′,2-anhydrocytidine **3C** was confirmed by spiking the NMR sample with a commercial sample and observing increased peak intensities. (Supplementary Figure 42).

*Oxidiation of 2-thio-α-ribo-cytidine **α-ribo-6C***

2-Thio-α-*ribo*-cytidine ***α-ribo*-6C** (6.48 mg, 25.0 µmol) in H_2O/D_2O (9:1, 450 µL) was submitted to *general procedure C* with H_2O_2 (30% w/w, 7.50 µL, 75.0 µmol) for 2.5 h. Analysis of the resultant solution by NMR spectroscopy showed *ribo*-2′,2-anhydrocytidine ***α-ribo*-3C** (80%) had formed. (Supplementary Figure 43).

*Oxidiation of 2-thio-ribo-cytidine **β-ribo*-6C**

2-Thio-*ribo*-cytidine **β-*ribo*-6C** (5.00 mg, 19.2 µmol) and potassium hydrogen phthalate **13** (0.100 M, 50.0 µL, 5.00 µmol in D_2O) were dissolved in glycine buffer (0.11 M, H_2O/D_2O, 9:1, 450 µL) and the pH of the solution was adjusted to pH 3. H_2O_2 H_2O_2 (30% w/w, 5.80 µL, 57.8 µmol) was then added to the solution and NMR spectra were periodically acquired. Analysis of the acquired NMR spectra showed *ribo*-cytidine **β-*ribo*-4C** (24%) and 4-amino-pyrimidine-riboside **β-*ribo*-9** (76%) had been afforded after 17 h. (Supplementary Figure 44).

2-Thio-*ribo*-cytidine **β-*ribo*-6C** (5.00 mg, 19.2 µmol) and potassium hydrogen phthalate **13** (0.100 M, 50.0 µL, 5.00 µmol in D_2O) were dissolved in phosphate buffer (0.11 M, H_2O/D_2O, 9:1, 450 µL) and the pH of the solution was adjusted to pH 7. Two aliquots of H_2O_2 H_2O_2 (30% w/w, 5.80 µL, 57.8 µmol) were added over 7 h and NMR spectra were periodically acquired. Analysis of the acquired NMR spectra showed rib*o*-cytidine **β-*ribo*-4C** had been afforded in quantitative conversion after 7 h. The identity of *ribo*-cytidine **β-*ribo*-4C** was confirmed by spiking the NMR sample with a commercial sample and observing increased peak intensities. (Supplementary Figure 44).

2-Thio-*ribo*-cytidine **β-*ribo*-6C** (5.00 mg, 19.2 µmol) and potassium hydrogen phthalate **13** (0.100 M, 50.0 µL, 5.00 µmol in D_2O) were dissolved in phosphate buffer (0.11 M, H_2O/D_2O, 9:1, 450 µL) and the pH of the solution was adjusted to pH 9. H_2O_2 H_2O_2 (30% w/w, 5.80 µL, 57.8 µmol) was added the solution and NMR spectra were periodically acquired. Analysis of the acquired NMR spectra showed *ribo*-cytidine **β-*ribo*-4C** had been afforded in 96% conversion after 6 h. (Supplementary Figure 44).

*Simultaneous acid oxidation of 2-thio-ribo-cytidine **β-ribo-6C** and 4-thio-arabino-uridine **ara-7U***

2-Thio-*ribo*-cytidine **β-*ribo*-6C** (2.50 mg, 9.63 µmol) and 4-thio-*arabino*-uridine ***ara*-7U** (2.50 mg, 9.63 µmol) potassium hydrogen phthalate **13** (0.100 M, 50.0 µL, 5.00 µmol in D_2O) were dissolved in glycine buffer (0.11 M, H_2O/D_2O, 9:1, 450 µL) and the pH of the solution was adjusted to pH 3. H_2O_2 (5.80 µL, 57.8 µmol) was then added to the solution and NMR spectra were periodically acquired. After 18 h a further aliquot of H_2O_2 (5.80 µL, 57.8 µmol) was added and NMR spectra were taken after 1 h. Analysis of the acquired NMR spectra showed *arabino*-uridine ***ara*-4U** (92%), *ribo*-cytidine **β-*ribo*-4C** (20%) and 4-amino-pyrimidine-riboside **β-*ribo*-9** (72%) had been furnished. The identity of *ribo*-cytidine **β-*ribo*-4C** and *arabino*-uridine ***ara*-4U** were confirmed by spiking the NMR sample with commercial samples of **β-*ribo*-4C** and ***ara*-4U**, and observing increased peak intensities. (Supplementary Figure 45).

2-step one-pot formation of arabino-nucleosides

2′,2-Anhydrocytidine **3C** (6.50 mg, 25.0 µmol), 2′,8-anhydroadenosine **3A** (6.70 mg, 25.0 µmol) and 2′,8-anhydroguanosine **3G** (6.50 mg, 25.0 µmol) were dissolved in H_2O/D_2O (9:1, 1.35 mL) and combined with a solution of potassium hydrogen phthalate **13** in D_2O (0.100 M, 150.0 µL, 15.00 µmol). NMR spectra were acquired of the resultant solution and an initial 32:34:34 ration of **3C**/**3A**/**3G** was observed. Disodium sulfide nonahydrate (360 mg, 1.50 mmol) was added to the nucleotide solution,

which was then submitted to *general procedure A* for 7 d. After 7 d NMR spectra were acquired, which showed 8-mercapto-*arabino*-adenosine ***ara*-5A** (65%), 8-mercapto-*arabino*-guanosine ***ara*-5G** (62%), *arabino*-cytidine ***ara*-4C** (55%) and 4-thio-*arabino*-uridine ***ara*-7U** (35%) had been furnished. This nucleotide mixture was submitted to *general procedure C* with H_2O_2 H_2O_2 (30% w/w, 115 µL, 1.13 mmol) for 1 h. Analysis of the resultant solution by NMR spectroscopy showed *arabino*-adenosine ***ara*-4A** (53%), *arabino*-guanosine ***ara*-4G** (62%), *arabino*-uridine ***ara*-4U** (35%) and *arabino*-cytidine ***ara*-4C** (47%) had been furnished (over 2 steps). The identity of *arabino*-guanosine ***ara*-4G**, *arabino*-cytidine ***ara*-4C**, *arabino*-uridine ***ara*-4U** and *arabino*-adenosine ***ara*-4A** were all confirmed by spiking the NMR sample with commercial samples of ***ara*-4G**, ***ara*-4C**, ***ara*-4U** and ***ara*-4A** and observing increased peak intensities. (Supplementary Figure 46).

2′,8-Anhydroguanosine (3G)

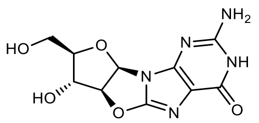

8-Bromo-*arabino*-guanosine ***ara*-16G** (100 mg, 277 µmol) and ammonium formate (72.0 mg, 1.15 mmol) were suspended in H_2O (20.0 mL). The observed pH of the suspension was adjusted to pH 9 with aqueous ammonia solution (28% w/w). The resultant suspension was then stirred at room temperature overnight, after which point all solids had dissolved. The solution was then lyophilised. The resultant lyophilisate was dissolved in water (20.0 mL) and lyophilised three times. The third and final lyophilisation afford 56.5 mg of 2′,8-anhydroguanosine **3G** (73%) as a white powder. δ_H (600 MHz, D_2O): 6.58 (1H, d, *J* = 5.5 Hz, H1′), 5.81 (1H, d, *J* = 5.5 Hz, H2′), 4.66 (1H, m, H3′), 4.37 (1H, m, H4′), 3.56 (1H, ABX, *J* = 12.6, 4.6 Hz, H5′), 3.46 (1H, ABX, *J* = 12.6, 6.0 Hz, H5″); δ_C (151 MHz, D_2O): 158.8 (C8), 158.6, 154.3, 148.5, 117.1 (C5), 99.0 (C2′), 89.6 (C4′), 86.4 (C1′), 75.6 (C4′), 61.5 (C5′). HRMS (ESI): for $C_{10}H_{12}N_5O_5$ $[M+H]^+$ calculated 282.0838; found 282.0841. IR (cm^{-1}): 3121 (OH/NH_2), 1686, 1600 (CO-NH), 1504, 1443 (Ar). $[\alpha]_D^{20.0}$ -59.8 (c = 0.93, DMSO).

***arabino*-Inosine (*ara*-4I)**

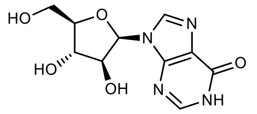

arabino-Adenosine ***ara*-4A**[7] (66.8 mg, 0.250 mmol) was suspended in acetic acid solution (2.00 M, 33.2 mL). $NaNO_2$ (173 mg, 2.50 mmol) was added to the solution (and a gas bubbler containing silicone oil was attached to the reaction exhaust port). The solution was stirred at room temperature and NMR spectra of the solution were periodically acquired. A further portion of $NaNO_2$ (300 mg, 4.35 mmol) was added after 42 h. The reaction was judged to be complete 6 h after the second addition of $NaNO_2$, and the resultant solution was then concentrated *in vacuo* to viscous oil. The residue was co-evaporated with toluene (30.0 mL × 2) and the resultant solids were dissolved in boiling H_2O (10.0 mL). The resultant aqueous solution was allowed to cool to room temperature and a precipitate was observed to form. After 24 h the precipitate was isolated by filtration to afford 31.6 mg of *arabino*-inosine ***ara*-4I** (47%) as white crystals. δ_H (600 MHz, D_2O): 8.37 (1H, s, H8), 8.20 (1H, s, H2), 6.43 (1H, d, *J* = 5.9, H1′), 4.59 (1H, t, *J* = 5.9 Hz, H2′), 4.38 (1H, t, *J* = 5.9 Hz, H3′), 4.09 (1H, m, H4′), 3.99 (1H, ABX, *J* = 12.7, 3.1 Hz, H5′), 3.92 (1H, ABX, *J* = 12.7, 5.0 Hz, H5″); δ_C (151 MHz, D_2O): 159.2 (C6), 149.2 (C4), 146.7 (C2), 141.5 (C8), 124.0 (C5), 85.0 (C1′), 83.5 (C4′), 76.6 (C2′), 74.7 (C3′), 61.1 (C5′). HRMS (ESI): for $C_{10}H_{13}N_4O_5$ calculated 269.0886 [M+H], observed 269.0884. IR (cm^{-1}): 3248 (O-H), 1677-1592 (CO-NH), 1551, 1478 (Ar). M.P.: 230.0-233.8 °C (decomp), (lit. 234-236 °C)[8]. $[\alpha]_D^{20.0}$ +15.00 (c = 0.74, H_2O).

8-Mercapto-*arabino*-adenosine (*ara*-5A)

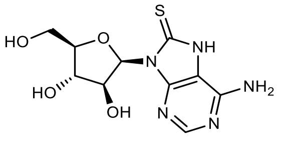

Disodium sulfide nonahydrate (3.60 g, 15.0 mmol), H_2O (15.0 mL) and 2′,8-anhydroadenosine **3A** (349 mg, 1.32 mmol) were submitted to *general procedure A* for 3 d. The product was then purified by FCC (eluting with 5-100% MeOH in $CHCl_3$) and those fractions containing 8-mercapto-*arabino*-adenosine ***ara*-5A** were concentrated *in vacuo*. The resultant solids were then purified by RP-FCC (eluting with 5-100% MeOH in H_2O) and those fractions containing only 8-mercapto-*arabino*-adenosine ***ara*-5A** were concentrated *in vacuo* to afford 175 mg of 8-mercapto-*arabino*-adenine ***ara*-5A** (44%) as a yellow solid. The remaining fractions (collected from RP-FCC) that contained 5',8-anhydroadenosine **10A** were concentrated *in vacuo* and the residue was dissolved in H_2O (2.00 mL). A white crystalline solid was observed to precipitate from this aqueous solution over 24 h. The precipitate was isolated by filtration to afford a further 8.3 mg of 5′,8-anhydroadenosine **10A** (3%) colourless crystals. δ_H (600 MHz, D_2O): 8.13 (1H, s, H2), 6.93 (1H, d, J = 7.0 Hz, H1′), 4.74 (1H, t, J = 7.0 Hz, H3′), 4.70 (1H, t, J = 7.0 Hz, H2′), 4.07 (1H, ddd, J = 7.0, 3.7, 2.7 Hz, H4′), 4.05 (1H, ABX, J = 12.7, 2.7 Hz, H5′), 3.99 (1H, ABX, J = 12.7, 3.7 Hz, H5″); δ_C (151 MHz, D_2O): 171.3 (C8), 156.1 (C2), 155.0 (C6), 154.6 (C4), 117.5 (C5)†, 90.7 (C1′), 86.7 (C4′), 81.7 (C2′), 79.3 (C3′), 65.5 (C5′). † Observed by HMBC. HRMS (ESI): for $C_{10}H_{14}N_5SO_4$ $[M+H]^+$ calculated 300.0761; found 300.0764. IR (cm^{-1}): 3175 (OH/NH_2), 1648 (Ar). M.P.: 170.7 °C (173.5 °C decomp), (lit. 154 °C[2] or 199.5-202.5 °C)[9]. λ_{max}: 305, 298, 238 nm. $[\alpha]_D^{20.0}$ +13.4 (c = 0.07, H_2O).

8-Mercapto-*arabino*-guanosine (*ara*-5G)

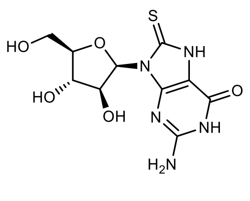

Disodium sulfide nonahydrate (1.50 g, 6.25 mmol) and 2′,8-anhydroguanosine **3G** (110 mg, 391 μmol) were dissolved H_2O (6.25 mL) and then submitted to *general procedure A* for 3.5 d. The product was then purified by FCC (eluting with 5-100%, MeOH in $CHCl_3$) and those fractions containing 8-mercapto-*arabino*-guanosine ***ara*-5G** were concentrated *in vacuo*. The resultant residue was the purified by RP-FCC (eluting with 5-100%, MeOH in H_2O). The fractions containing 8-mercapto-*arabino*-guanosine ***ara*-5G** were concentrated *in vacuo* affording 19.0 mg of 8-mercapto-*arabino*-guanosine ***ara*-5G** (15%) as a white solid. δ_H (600 MHz, *d6*-DMSO): 12.82 (1H, brs, NH), 11.19 (1H, brs, NH), 6.65 (1H, d, J = 7.3, H1′), 6.46 (2H, brs, NH_2), 5.40 (1H, d, J = 5.7, 2′-OH), 5.17 (1H, d, J = 4.9, 3′-OH), 4.80 (1H, brs, 5′-OH), 4.43 (1H, m, H3′), 4.22 (1H, q, J = 6.3, H2′), 3.73 (1H, m, H5′), 3.68 (1H, m, H5″), 3.62 (1H, td, J = 6.9, 1.2, H4′); δ_C (151 MHz, *d6*-DMSO): 164.9, 149.9, 153.5, 151.6, C5 not observed, 84.1 (C1′), 82.8 (C4′), 76.9 (C2′), 75.6 (C3′), 62.0 (C5′). HRMS (ESI): for $C_{10}H_{13}N_5O_5S$ $[M+H]^+$ calculated 316.0710, found 316.0710. IR (cm^{-1}): 3319, 3207, 3115, 2942, 2919 ($OH/NH/NH_2$), 1649 ($CO-NH_2$), 1595-1440 (Ar). $[\alpha]_D^{20.0}$ -17.2 (c = 0.35, DMSO).

8-Mercapto-*arabino*-inosine (*ara*-5I)

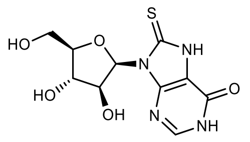

Disodium sulfide nonahydrate (1.50 g, 6.20 mmol) and 2′,8-anhydroinosine **3I** (147 mg, 552 μmol) were dissolved H_2O (6.25 mL) and then submitted to *general procedure A* for 4 d. The product was then purified by FCC (eluting with 5-100%, MeOH in $CHCl_3$) and those fractions containing 8-mercapto-*arabino*-inosine ***ara*-5I** were concentrated *in vacuo*. The residue was then further purified by RP-FCC (eluting with 5-100%, MeOH in H_2O). The fractions containing 8-mercapto-*arabino*-inosine ***ara*-5I** were concentrated *in vacuo* affording 116.4 mg of 8-mercapto-*arabino*-inosine ***ara*-5I** (70%) as a white solid. δ_H (600 MHz, D_2O): 8.20 (1H, s, H2), 7.00 (1H, d, J = 6.9 Hz, H1′), 4.80 (1H, m, H3′), 4.76 (1H,

t, *J* = 6.9 Hz, H2′), 4.09 (1H, m, H4′), 4.07 (2H, m, H5′ + H5″); δ_C (151 MHz, D_2O): 166.9 (C8), 154.1 (C6), 149.1 (C4), 146.0 (C2), 115.8 (C5), 86.0 (C1′), 82.3 (C4′), 76.9 (C2′), 75.3 (C3′), 61.6 (C5′). HRMS (ESI): for $C_{10}H_{13}N_4O_5S$ $[M-H]^-$ calculated 299.0450; found 299.0454. IR (cm^{-1}): 3218, 2845 (O-H), 1680, 1555, 1504 (Ar). λ_{max}: 292, 215 nm. $[\alpha]_D^{20.0}$ +15.77 (c = 0.26, H_2O).

8-Mercapto-*ribo*-adenosine (*ribo*-5A)

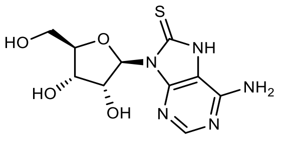

Thiourea (30.0 mg, 0.406 mmol) was added to 8-bromo-*ribo*-adenosine ***ribo*-16A**[10] (100 mg, 0.277 mmol) in ethanol (5.00 mL). The solution was refluxed for 4 h and then concentrated *in vacuo*. The product was then purified by RP-FCC (eluting with 2.5-100%, MeOH in H_2O) affording 80.0 mg of 8-mercapto-*ribo*-adenosine ***ribo*-5A** (93%) as an off white powder. δ_H (600 MHz, D_2O): 8.09 (1H, s, H2), 6.57 (1H, dd, *J* = 7.0, 1.6 Hz, H1′), 5.03 (1H, m, H2′), 4.50 (1H, m, H3′), 4.30 (1H, d, *J* = 2.5 Hz, H4′), 3.95 (1H, ABX, *J* = 12.9, 2.5 Hz, H5′), 3.86 (1H, ABX, *J* = 12.9, 2.5 Hz, H5″); δ_C (151 MHz, D_2O): 167.8 (C8), 152.7 (C2), 149.1 (C6), 148.6 (C4), 109.2 (C5), 89.7 (C1′), 86.9 (C4′), 72.9 (C2′), 71.7 (C3′), 62.8 (C5′). HRMS (ESI): for $C_{10}H_{13}N_5O_4S$ $[M+H]^+$ calculated 300.0761 [M+H], found 300.0766. IR (cm^{-1}): 3323 broad (OH/NH_2), 1648, 1555, 1478, 1449 (Ar). λ_{max}: 306, 299, 237 nm. $[\alpha]_D^{20.0}$ -39.8 (c = 0.57, H_2O).

8-Mercapto-*ribo*-guanosine (*ribo*-5G)

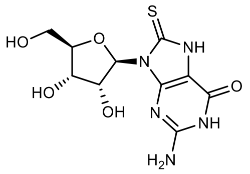

Thiourea (167 mg, 2.20 mmol) was added to 8-bromo-*ribo*-guanosine ***ribo*-16G** (400 mg, 1.1 mmol) in ethanol (4.0 mL). The solution was refluxed overnight and then concentrated *in vacuo*. The product was then recrystallized from H_2O (30.0 mL), affording 340 mg of 8-mercapto-*ribo*-guanosine ***ribo*-5G** (Quant.) as an off white powder. *All data consistent with literature.*[11]

8-Mercapto-*ribo*-inosine (*ribo-5I*)

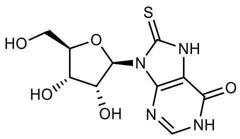

Tri-acetyl-8-bromo-*ribo*-inosine **18**[10] (498 mg, 1.05 mmol) was dissolved in methanolic ammonia (7.00M, 10.0 mL) and incubated for 6 h. The product was observed to crystallise directly from solution and was isolated by filtration in two crops to give a 189.5 mg crude 8-bromo-*ribo*-inosine ***ribo*-16I** (52% crude), which was used without further purification. A portion of 8-bromo-*ribo*-inosine ***ribo*-16I** (126.8 mg, 0.365 mmol) and thiourea (80.0 mg, 1.05 mmol) were suspended in ethanol (5.00 mL) and refluxed overnight. The solution was cooled to room temperature and concentrated *in vacuo*. The crude product was then purified by RP-FCC (eluting with 5-100% MeCN in H_2O). The fractions containing 8-mercapto-*ribo*-inosine ***ribo*-5I** were concentrated *in vacuo* and the product was recrystallised from H_2O (4.00 mL), affording 100 mg 8-mercapto-*ribo*-inosine ***ribo*-5I** (47% over 2 steps; 91% for 2nd step) as a white powder. δ_H (600 MHz, noesygppr1d, D_2O): 8.24 (1H, s, H2), 6.67 (1H, d, *J* = 5.9 Hz, H1′), 5.16 (1H, t, *J* = 5.9 Hz, H2′), 4.63 (1H, dd, *J* = 5.9, 3.9 Hz, H3′), 4.32 (1H, m, H4′), 4.00 (1H, ABX, *J* = 12.7, 2.9 Hz, H5′), 3.94 (1H, ABX, *J* = 12.7, 4.3 Hz, H5″). δ_C (151 MHz, D_2O): 167.9 (C8), 153.4 (C6), 148.1 (C4), 146.7 (C2), 114.9 (C5), 89.8 (C1′), 86.2 (C4′), 72.8 (C2′), 71.2 (C3′), 62.5 (C5′). HRMS (ESI+): for $C_{10}H_{13}N_4O_5S$ $[M+H]^+$ calculated 301.0601, found 301.0600. IR (cm^{-1}): 3348, 2921 (OH/NH_2), 1664 (CO-NH_2), 1566, 1502, 1468, 1444 (Ar). M.P.: 239.4 °C (decomp) (lit. 182 - 185 °C, decomp).[12] $[\alpha]_D^{20.0}$ -33.1 (c = 0.57, DMSO).

2-Thio-*arabino*-cytidine (*ara*-6C)

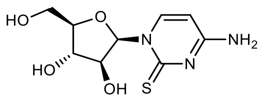

Sodium hydrogen sulfide hydrate (81.0 mg, 0.764 mmol), ammonium bicarbonate (60.0 mg, 0.764 mmol) and *arabino*-2′,2-anhydrocytidine **3C** (50.0 g, 0.191 mmol) were dissolved in DMF (400 µL) and stirred at room temperature overnight. The solution was then concentrated *in vacuo* and the product purified by FCC (eluting with a gradient of 0-100%, EtOAc in petroleum ether, then eluting with a gradient of 0-50%, MeOH in EtOAc). The fractions containing 2-thio-*arabino*-cytidine ***ara*-6C** were concentrated *in vacuo* and then the product purified by RP-FCC (eluting with 5-100% MeOH in H_2O). The fractions containing 2-thio-*arabino*-cytidine ***ara*-6C** were concentrated *in vacuo* to afford 34.8 mg of 2-thio-*arabino*-cytidine ***ara*-6C** (68%) as a colourless film. δ_H (600 MHz, D_2O): 7.91 (1H, d, *J* = 7.7 Hz, H6), 6.94 (1H, d, *J* = 4.5 Hz, H1′), 6.27 (1H, d, *J* = 7.7 Hz, H5), 4.60 (1H, dd, *J* = 4.5, 3.7 Hz, H2′), 4.08 (1H, dd, *J* = 4.7, 3.7 Hz, H3′), 4.01 (1H, m, H4′), 3.88 (1H, ABX, *J* = 12.5, 3.5 Hz, H5′), 3.80 (1H, ABX, *J* = 12.5, 5.9 Hz, H5″); δ_C (151 MHz, D_2O): 179.4 (C2), 161.8 (C4) 143.7 (C6), 99.6 (C5), 91.0 (C1′), 84.4 (C4′), 76.2 (C3′), 75.4 (C2′), 61.3 (C5′). HRMS (ES+): for $C_9H_{14}N_3O_4S$ $[M+H]^+$ calculated 260.0705; found 260.0694. IR (cm^{-1}): 3308, 3201 (OH/NH_2), 1638, 1532, 1475 (Ar). $[\alpha]_D^{20.0}$ +196.5 (c = 0.20, H_2O).

4-Thio-*arabino*-uridine (*ara*-7U)

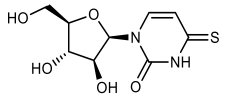

Disodium sulfide nonahydrate (4.80 g, 20.0 mmol), H_2O/D_2O (15.0 mL, 9:1) and *arabino*-cytidine ***ara*-4C** (243.2 mg, 1.00 mmol) were submitted to *general procedure A* for 7 d. The product was then purified by FCC (eluting with a gradient of 0-60%, MeOH in $CHCl_3$) affording separation of 4-thio-*arabino*-uridine ***ara*-7U** and *arabino*-cytidine ***ara*-4C**. Each fraction was separately purified further by RP-FCC (eluting with a gradient of 5-100% MeOH in H_2O), respectively affording 99.2 mg 4-thio-*arabino*-uridine ***ara*-7U** (38%) as a yellow film and 141.4 mg of *arabino*-cytidine ***ara*-4C** (58%) as a white solid, the identity of which was confirmed by spiking with a commercial sample of *arabino*-cytidine ***ara*-4C**. δ_H (600 MHz, D_2O): 7.69 (1H, d, *J* = 7.4 Hz, H6), 6.62 (1H, d, *J* = 7.4 Hz, H5), 6.19 (1H, d, *J* = 5.3 Hz, H1′), 4.44 (1H, t, *J* = 5.3 Hz, H2′), 4.14 (1H, t, *J* = 5.3 Hz, H3′), 4.04 (1H, td, *J* = 5.3, 3.3 Hz, H4′), 3.94 (1H, ABX, *J* = 12.5, 3.3 Hz, H5′), 3.85 (1H, ABX, *J* = 12.5, 5.3 Hz, H5″); δ_C (151 MHz, D_2O): 193.4 (C4), 152.2 (C2), 138.4 (C6), 113.7 (C5), 86.6 (C1′), 83.9 (C4′), 76.0 (C2′), 75.6 (C3′), 61.2 (C5′). HRMS (ESI): for $C_9H_{13}N_2O_5S$ $[M+H]^+$ calculated 261.0545; found 261.0559. IR (cm^{-1}): 3341, 2965 (OH/NH_2), 1699, 1611 (CO-NH). λ_{max}: 333, 245, 196 nm. $[\alpha]_D^{20.0}$ +259.8 (c = 0.31, H_2O).

4-Thio-*ribo*-uridine (β-*ribo*-7U)

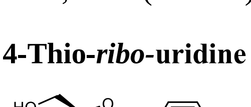

Disodium sulfide nonahydrate (480 mg, 2.00 mmol), H_2O/D_2O (2.00 mL, 9:1) and *ribo*-cytidine **β-*ribo*-4C** (24.4 mg, 0.100 mmol) were submitted to *general procedure A* for 7 d. The product was then purified by FCC (eluting with a gradient of 0-50%, MeOH in $CHCl_3$) affording 3.67 mg 4-thio-*ribo*-uridine **β-*ribo*-7U** (14%) as a yellow film. δ_H (600 MHz, D_2O): 7.72 (1H, d, *J* = 7.6 Hz, H6), 6.52 (1H, d, *J* = 7.6 Hz, H5), 5.83 (1H, d, *J* = 3.8 Hz, H1′), 4.30 (1H, dd, *J* = 5.1, 3.8 Hz, H2′), 4.16 (1H, m, H3′), 4.10 (1H, m, H4′), 3.89 (1H, ABX, *J* = 12.9, 2.6 Hz, H5′), 3.77 (1H, ABX, *J* = 12.9, 4.4 Hz, H5″); δ_C (151 MHz, D_2O): 191.1 (C4), 149.9 (C2), 137.5 (C6), 114.2 (C5), 90.7 (C1′), 84.9 (C4′), 74.6 (C2′), 69.8 (C3′), 61.2 (C5′). HRMS (ES+): for $C_9H_{12}N_2O_5SNa$ $[M+Na]^+$ calculated 283.0365; found 283.0377. IR (cm^{-1}): 3315 (OH), 2923 (SH), 1694, 1612 (Ar). $[\alpha]_D^{20.0}$ +32.1 (c = 0.01, H_2O).

4-Thio-α-*ribo*-uridine (*α-ribo*-7U)

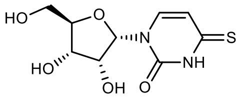

Disodium sulfide nonahydrate (480 mg, 2.00 mmol), H_2O/D_2O (2.00 mL, 9:1) and α-*ribo*-cytidine ***α-ribo*-4C** (24.4 mg, 0.100 mmol) were submitted to *general procedure A* for 8 d. The product was then purified by FCC (eluting with a gradient of 0-50%, MeOH in $CHCl_3$). The fractions containing 4-thio-α-*ribo*-uridine (***α-ribo*-7U**) were concentrated *in vacuo* affording 12.8 mg 4-thio-α-*ribo*-uridine ***α-ribo*-7U** (42%) as a yellow film. δ_H (600 MHz, D_2O): 7.68 (1H, d, J = 7.5 Hz, H6), 6.61 (1H, d, J = 7.5 Hz, H5), 6.16 (1H, d, J = 4.2 Hz, H1′), 4.53 (1H, t, J = 4.2 Hz, H2′), 4.33 (2H, m, H3′ + H4′), 3.93 (1H, ABX, J = 12.8, 2.4 Hz, H5′), 3.74 (1H, ABX, J = 12.8, 4.5 Hz, H5″); δ_C (151 MHz, D_2O): 191.5 (C4), 149.7 (C2), 139.0 (C6), 113.1 (C5), 87.8 (C1′), 84.1 (C4′), 71.3 (C2′), 70.7 (C3′), 61.4 (C5′). HRMS (ES+): for $C_9H_{13}N_2O_5S$ $[M+H]^+$ calculated 261.0545; found 261.0550. IR (cm^{-1}): 3221 (OH), 2924 (SH), 1682, 1601, 1459 (Ar). $[\alpha]_D^{20.0}$ -130.5 (c = 0.16, H_2O).

8-Oxo-3′,3-anhydroguanosine (12)

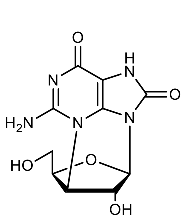

2′,8-Anhydroguanosine **3G** (50.0 mg, 177 µmol) was dissolved in NaOD solution (1.00 mL, 1 M, D_2O) and heated at 80 °C for 30 min. The solution pD was adjusted to pD 9 with DCl and the product purified by RP-FCC (eluting with 5% MeOH in H_2O). The fractions containing product were lyophilised and then the product was recrystallized from hot H_2O affording 7.6 mg of 8-oxo-3′,3-anhydroguanosine **12** (15%) as a white powder. δ_H (600 MHz, DMSO): 10.53 (1H, brs, NH), 6.90 (2H, brs, NH_2), 6.46 (1H, brs, 2′-OH), 5.43 (1H, s, H1′), 5.01 (1H, t, J = 5.1, 5′-OH), 4.99 (1H, dd, J = 3.5, 1.1, H3′), 4.66 (1H, brs, H2′), 4.46 (1H, ddd, J = 6.7, 5.0, 3.5, H4′), 3.41 (1H, m, H5′), 3.31 (1H, m, H5″); δ_C (151 MHz, D_2O): 158.8 (C6), 149.2 (C2), 148.6 (C8), 132.3 (C4), 100.1 (C5), 84.7 (C4′), 81.3 (C1′), 73.7 (C2′), 60.2 (C5′), 58.9 (C3′). HRMS (ESI): $C_{10}H_{11}N_5O_5$ predicted mass 282.0838 [M+H], found 282.0829. IR (cm^{-1}): 3282, 3177 (OH), 1720, 1687 (CO), 1589, 1565, 1512 (Ar). $[\alpha]_D^{20.0}$ +5.51 (c = 1.00, DMSO).

8-Bromo-*arabino*-adenosine (*ara*-16A)

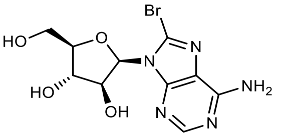

arabino-Adenosine ***ara*-4A**[12] (1.00 g, 3.74 mmol) was dissolved in sodium acetate buffer (1.00 M, 150 mL, pH 4). Bromine (13.1 mmol, 0.678 mL) was added and the solution was stirred at room temperature for 2 h. The H_2O was partially evaporated overnight by passing a steady stream of air over the solution, which resulted in the crystallization of ***ara*-16A**. The crystals (735 mg) were isolated by filtration and dissolved in boiling H_2O (2.00 mL). 10% Na_2SO_3 solution (2.00 mL) was added until the solution became colourless. The solution was cooled to room temperature resulting in the crystallization of ***ara*-16A**. The crystals (685 mg) were filtered which afforded 8-bromo-*arabino*-adenosine ***ara*-16A** (53%) as an off-white powder. δ_H (600 MHz, *d*6-DMSO): 8.07 (1H, s, H2), 7.46 (2H, bs, NH_2), 6.23 (1H, d, J = 6.6 Hz, H1′), 5.65 (1H, d, J = 6.6 Hz, 2′-OH), 5.50 (1H, d, J = 5.6 Hz, 3′-OH), 5.35 (1H, dd, J = 6.3, 4.5 Hz, 5′-OH), 4.45 (1H, q, J = 6.6 Hz, H3′), 4.35 (1H, q, J = 6.6 Hz, H2′), 3.75 (3H, m, H4′ + H5′ + H5″); δ_C (151 MHz, *d*6-DMSO): 155.2 (C6), 151.8 (C2), 150.5 (C4), 126.6 (C8), 119.3 (C5), 86.3 (C1′), 82.9 (C4′), 76.6 (C2′), 74.3 (C3′), 61.1 (C5′). HRMS (ESI): for $C_{10}H_{13}BrN_5O_4$ $[M+H]^+$ calculated 346.0145; found 346.0145. IR (cm^{-1}): 3372, 3306, 3173 (broad, OH/NH_2), 1665, 1601, 1574 (Ar). M.P.: 212.3 °C (decomp) (lit. 202-204 °C)[12]. $[\alpha]_D^{20.0}$ -16.4 (c = 1.00, DMSO).

8-Bromo-*arabino*-guanosine (*ara*-16G)

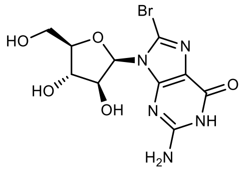

Saturated aqueous bromine (30.0 mL) was added to a stirred slurry of *arabino*-guanosine ***ara*-4G**[13] (875 mg, 3.09 mmol) in H_2O (20.0 mL) over 20 min at room temperature. The slurry was stirred for a further 1 h and then the solids were separated by filtration. The solids were recrystallized from boiling H_2O/DMSO (3:1, 30.0 mL) affording 982 mg of 8-bromo-*arabino*-guanosine ***ara*-16G** (85%) as a white powder. δ_H (600 MHz, *d*6-DMSO): 10.80 (1H, s, N1-H), 6.46 (2H, br s, NH_2), 6.06 (1H, d, *J* = 6.5 Hz, H1′), 5.55 (1H, d, *J* = 6.5 Hz, 2′-OH), 5.35 (1H, d, *J* = 6.5 Hz, 3′-OH), 4.85 (1H, t, *J* = 5.8 Hz, 5′-OH), 4.30 (1H, q, *J* = 6.5 Hz, H3′), 4.22 (1H, q, *J* = 6.5 Hz, H2′), 3.67 (3H, m, H4′ + H5′ + H5″). δ_C (151 MHz, *d*6-DMSO): 155.5 (C6), 153.2 (C2), 152.2 (C8), 120.5 (C4), 117.0 (C5), 85.1 (C1′), 83.1 (C4′), 76.7 (C2′), 75.3 (C3′), 61.7 (C5′). HRMS (ESI+): for $C_{10}H_{13}N_5O_5Br$ $[M+H]^+$ calculated 362.0095; found 362.0100. IR (cm^{-1}): 3340, 3155 (OH/NH_2), 1651, 1599, 1577, 1450 (Ar). M.P.: 215.7 °C NMR (decomp). $[\alpha]_D^{20.0}$ -12.3 (c = 0.92, DMSO).

8-Bromo-*ribo*-adenosine (*ribo*-16A)

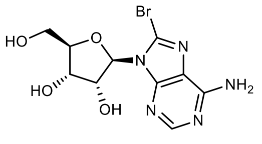

ribo-Adenosine ***ribo*-4A** (4.00 g, 14.98 mmol) and acetic acid (1.70 mL, 29.8 mmol) were dissolved in H_2O (300 mL) and pH of the solution was adjusted to pH 4 with NaOH (4.00 M). Bromine (2.68 mL, 52.4 mmol) was added to the solution and the solution was stirred at room temperature overnight. The pH of the solution was regularly measured and further NaOH (4.00 M) was added as necessary to keep the solution at pH 4. Completion of the reaction was monitored by NMR spectroscopy. Upon complete consumption of ***ribo*-4A**, saturated $NaHSO_3$ solution was added until the solution became colourless. Crystals of product were observed to form, these crystal were isolated by filtration and repeatedly suspended with toluene (10.0 mL × 3) and concentrated *in vacuo*. This afforded 799 mg of 8-bromo-*ribo*-adenosine ***ribo*-16A** (16%) as an off white powder. δ_H (600 MHz, D_2O): 8.11 (1H, s, H2), 6.08 (1H, d, *J* = 7.1 Hz, H1′), 4.98 (1H, dd, *J* = 7.1, 5.4 Hz, H2′), 4.43 (1H, dd, *J* = 5.4, 2.5 Hz, H3′), 4.27 (1H, q, *J* = 2.5 Hz, H4′), 3.89 (1H, ABX, *J* = 13.0, 2.5 Hz, H5′), 3.80 (1H, ABX, *J* = 13.0, 2.5 Hz, H5″); δ_c (151 MHz, D_2O): 155.3 (C6), 152.9 (C2), 150.0 (C4), 128.8 (C8), 120.7 (C5), 91.1 (C1′), 73.5 (C2′), 72.2 (C3′), 88.0 (C4′), 62.7 (C5′). HRMS (ESI): for $C_{10}H_{13}BrN_5O_4$ $[M+H]^+$ calculated 346.0145, found 346.0146. IR (cm^{-1}): 3375 broad (OH/NH_2), 1638, 1578, 1470 (Ar). M.P.: 178.8 °C (decomp) (lit. >200 °C).[12] $[\alpha]_D^{20.0}$ -23.8 (c = 0.93, H_2O).

8-Bromo-*ribo*-guanosine (*ribo*-16G)

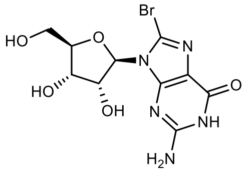

Saturated bromine H_2O (30.0 mL) was added to a stirred slurry of *ribo*-guanosine ***ribo*-4G**[13] (1.06 g, 3.74 mmol) in H_2O (20.0 mL) over 20 min at room temperature. The slurry was stirred for a further 5 min and then the solids were isolated by filteration. The solids were then recrystallized from boiling H_2O (30.0 mL) affording 1.28 g of 8-bromo-*ribo*-guanosine ***ribo*-16G** (94%) as white crystals. δ_H (600 MHz, *d*6-DMSO): 10.84 (1H, s, H1), 6.52 (2H, bs, NH_2), 5.68 (1H, d, *J* = 6.1 Hz, H1′), 5.46 (1H, d, *J* = 6.1 Hz, 2′-OH), 5.10 (1H, d, *J* = 5.3 Hz, 3′-OH), 5.01 (1H, q, *J* = 6.1 Hz, H2′), 4.93 (1H, t, *J* = 6.1 Hz, 5′-OH), 4.13 (1H, m, H3′), 3.85 (1H, m, H4′), 3.65 (1H, m, H5′), 3.51 (1H, m, H5″). δ_C (151 MHz, *d*6-DMSO): 155.5, 153.5 , 152.2 (C4), 121.5 (C8), 117.9 (C5), 89.7 (C1′), 86.0 (C4′), 70.6 (C3′), 70.3 (C2′), 62.3 (C5′). HRMS (ESI+): for $C_{10}H_{13}N_5O_5Br$ $[M+H]^+$ calculated 362.0095; found 362.0096. IR (cm^{-1}): 3095

(broad, OH/NH$_2$), 2931 (C-H) 1678, 1618, 1590, 1562, 1521 (Ar). M.P.: 173.1 °C (decomp) (lit. 201-203 °C).[11] $[\alpha]_D^{20.0}$ -63.5 (c = 1.00, H_2O).

Tri-acetyl-*ribo*-inosine (17)

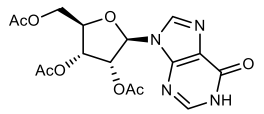

The reaction was adapted from a literature procedure.[14] *ribo*-Adensoine ***ribo*-4A** (5.00 g, 18.7 mmol) was dissolved in acetic acid (2.00 M, 217 mL). $NaNO_2$ (6.45 g, 93.5 mmol) was added and the solution was stirred overnight at room temperature. The progress of the reaction was monitored by NMR spectroscopy. Another portion of $NaNO_2$ (6.45 g, 93.5 mmol) was added after 16 h and the solution was stirred overnight at room temperature. The solution was concentrated *in vacuo* and the residue coevaporated with toluene (100 mL × 2). The resultant solids were suspended in pyridine (163 mL), cooled to 0 °C and acetic anhydride (21.2 mL, 224 mmol) was added. The syolution was stirred overnight at room temperature. Ethanol (40.0 mL) was added and the solution was stirred for 1 h. The mixture was concentrated *in vacuo*, coevaporated with toluene (100 mL × 2) and diluted with H_2O (60.0 mL). After 30 min stirring, a precipitate was observed. The precipitate was isolated by filtration and then washed with H_2O (10 mL) and EtOAc (1 mL). The solid was dried in a vacuum desiccator overnight to afford 7.40 g of tri-acetyl-*ribo*-inosine **17** (Quant.) as an off white powder, which was used without further purification. δ_H (600 MHz, $CDCl_3$): 8.16 (1H, s, H2), 8.00 (1H, s, H8), 6.16 (1H, d, J = 5.3 Hz, H1′), 5.87 (1H, t, J = 5.3 Hz, H2′), 5.60 (1H, t, J = 5.3 Hz, H3′), 4.46 (1H, obs. m, H4′), 4.44 (1H, ABX, J = 12.1, 3.2 Hz, H5′), 4.38 (1H, ABX, J = 12.1, 4.2 Hz, H5″), 2.16 (3H, s, C$\underline{H}_3$), 2.15 (3H, s, C$\underline{H}_3$), 2.11 (3H, s, 2′-OCOC$\underline{H}_3$); δ_C (151 MHz, D_2O): 170.5 (C5′-O$\underline{C}$O), 169.8 (C3′-O$\underline{C}$O), 165.5 (C2′-O$\underline{C}$O), 158.9 (C6), 148.7 (C4), 145.3 (C2), 138.8 (C8), 125.6 (C5), 86.7 (C1′), 80.5 (C4′), 73.6 (C2′), 70.7 (C3′), 63.1 (C5′), 21.0 (CH_3), 20.7 (CH_3), 20.6 (CH_3). HRMS (ESI): for $C_{16}H_{19}N_4O_8$ $[M+H]^+$ calculated 395.1197; found 395.1193. IR (cm^{-1}): 3050, 2963, 2803 (OH/NH), 1744, 1702 (CO), 1589, 1554 (Ar). $[\alpha]_D^{20.0}$ -68.3 (c = 0.89, $CHCl_3$).

Tri-acetyl-8-bromo-*ribo*-inosine (18)

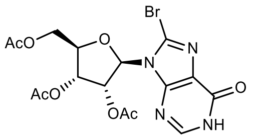

Disodium hydrogen phosphate (7.50 g, 52.8 mmol) and bromine (2.00 mL, 39.0 mmol) were dissolved in H_2O (75.0 mL). The bromine solution was then added to a solution of tri-acetyl-*ribo*-inosine **17**[14] (1.97 g, 5.00 mmol) in 1,4-dioxane (75.0 mL). The solution was stirred for 4 d and analysed by TLC. A further portion of bromine (2.00 mL, 39.0 mmol) was added and the solution was stirred for 3 d. The solution was then cooled to 0 °C and saturated $NaHSO_3$ solution was added dropwise until the solution turned colourless. The product was extracted with CH_2Cl_2 (3 × 75 mL). The combined organic layers were washed with H_2O (50.0 mL), dried over magnesium sulfate, filtered and concentrated *in vacuo*. The crude product was purified by FCC (eluting with 0-5% MeOH in CH_2Cl_2) affording 676 mg of tri-acetyl-8-bromo-*ribo*-inosine **18** (29%) as off-white foam. δ_H (600 MHz, $CDCl_3$): 12.66 (1H, brs, NH), 8.30 (1H, s, H2), 6.14 (1H, dd, J = 6.0, 4.8 Hz, H2′), 6.01 (1H, d, J = 4.8 Hz, H1′), 5.70 (1H, t, J = 6.0 Hz, H3′), 4.39 (1H, ABX, J = 11.9, 3.8 Hz, H5′), 4.30 (1H, ddd, J = 6.1, 6.0, 3.8 Hz, H4′), 4.21 (1H, ABX, J = 11.9, 6.1 Hz, H5″), 2.04 (3H, s, C3′-OCOC$\underline{H}_3$), 2.00 (3H, s, C2′-OCOC$\underline{H}_3$), 1.96 (3H, s, C5′-OCOC$\underline{H}_3$); δ_C (151 MHz, D_2O): 170.6 (C5′-O$\underline{C}$O), 169.7 (C3′-O$\underline{C}$O), 169.5 (C2′-O$\underline{C}$O), 156.9 (C6), 149.6 (C4), 144.5 (C2), 126.5 (C8), 125.8 (C5), 88.9 (C1′), 80.3 (C4′), 72.2 (C2′), 70.5 (C3′), 63.2 (C5′), 20.9 (C5′-OCO$\underline{C}H_3$), 20.7 (CH_3), 20.5 (CH_3). HRMS (ESI+): for $C_{16}H_{18}N_4O_8Br$ $[M+H]^+$ calculated

473.0303; found 473.0302. IR (cm^{-1}): 3117, 3061, 2934, 2866 (OH/NH), 1745, 1686 (CO), 1587, 1548 (Ar). $[\alpha]_D^{20.0}$ -7.4 (c = 1.41, $CHCl_3$).

Crystal growth and X-ray diffraction studies

Single crystals of ***ara*-5A** suitable for X-ray diffraction analyses were obtained by dissolving compound ***ara*-5A** in water at a concentration of 66.9 mM. The solution was incubated at room temperature in a sealed vial. The formation of yellow needles was observed after 1 d. Single crystals of **12** suitable for X-ray diffraction analyses were obtained by slowing cooling a saturating solution of **12** in hot water to room temperature. The formation of colourless needles was observed after 1 d. Crystallographic and refinement parameters for the crystal structures of ***ara*-5A** and **12** are given in Supplementary Table 3. The molecular structure of ***ara*-5A** is shown in Supplementary Figure 120 and the molecular structure of **12** is shown in Supplementary Figure 121.

Supplementary Discussion

Ground state geometries of ***ara*-5A, *ara*-5I** and ***ara*-5G** were optimized at the MP2/cc-pVTZ level of theory. In order to choose a reasonable starting conformer we tested several possibilities including the C2′-endo and C3′-endo conformers of arabinosides. All of the optimizations of the C2′-endo initial geometries converged to the C3′-endo structure, indicating dominant stability of the latter conformer. We further considered the energies the different orientations of the 8-mercapto-nucleobase with respect to the sugar moiety. Typically in the case of purine nucleosides the *syn*- orientation of the nucleobase enables the formation of a C5′-OH...N3 hydrogen bond, which additionally stabilizes this molecular arrangement. This intramolecular hydrogen bond is formed in all the studied 8-mercapto-arabinosides and as a result the *syn* conformers are lower in energy by approximately 0.25 – 0.3 eV than the *anti* conformers. Therefore, we considered the C3′-endo-*syn* conformers in the excited-state calculations of 8-mercapto-arabinosides ***ara*-5A**, ***ara*-5I** and ***ara*-5G**, however in the ESA spectra calculations the *anti* conformer of ***ara*-5A** showed better correlation between the experiment and simulated bands.

Vertical excitation energies calculated for the C3′-endo-*syn* conformers of the studied 8-mercapto-*arabino*-purine are shown in Supplementary Table 1. Arabinosides ***ara*-5A**, ***ara*-5I** and ***ara*-5G** were predicted to exhibit significant absorption at the excitation wavelength of 300 nm used in the synthesis experiments. These results suggested that in the case of ***ara*-5A** the $\pi\pi_{CS}$* excitation should be the dominant excited state populated during the irradiation at 300 nm. In contrast, we expect the initial population of a mixture of $\pi\pi_{ring}$* and $\pi\pi_{CS}$* states during the irradiation of ***ara*-5I** and ***ara*-5G**.

Spin-orbit coupling matrix elements (SOCMEs) calculated at the CASPT2 level are presented in Supplementary Table 2. All the SOCMEs were computed in the respective minima of the initial state indicated below. We did not calculate the corresponding SOCMEs with the manifold of triplet electronic states, since the local minimum corresponding to the $S_1(\pi\pi_{ring}*)$ state coincides with the conical intersection with the ground state. However, we expect that the $S_1(\pi\pi_{ring}*) \rightarrow T_n$ intersystem crossings will contribute to the overall triplet population in ***ara*-5I**, during the initial photodynamics, *i.e.* before this conical intersection is reached.

S_1 minimum energy geometries corresponding to the singlet $\pi\pi_{CS}$* state are presented in Supplementary Figure 47. The $\pi\pi_{CS}$* excitation is localized on the thiocarbonyl group, therefore these geometries are

characterized by lengthening of the C=S bond (by ~0.1 A) and significant pyramidalization of the C8 carbon atom. The location of the unpaired electron on C8 and S in fact results in the formation of a single C—S bond instead of the double bond characteristic for the ground-state geometry. This indicates the the states having the $\pi\pi_{CS}$* character might become precursors of the sulfur loss process. As indicated above, the minima of the $S_1(\pi\pi_{ring}{}^*)$ coincide with the S_1/S_0 conical intersections, and an example geometry of such state crossing is provided in Supplementary Figure 48.

The S_1/S_0 conical intersection geometries, which could enable photodeactivation to the electronic ground state and prevent the population of triplet states, are presented in Supplementary Figure 48. All the $^1\pi\pi_{CS}{}^*/S_0$ state crossing geometries found in the 8-mercapto-*arabino*-purines exhibit the same features independently of the nucleobase. In particular, the sloped topography of these conical intersections indicates that once the $S_1(\pi\pi_{CS}{}^*)$ minimum is populated, the photodeactivation to the electronic ground-state would not be an immediate process considering that the $^1\pi\pi_{CS}{}^*/S_0$ state crossings are approximately 0.3 eV higher in energy. Even though this energy difference is modest, it may facilitate the population of triplet states by hindering the direct repopulation of the closed-shell electronic ground state.

***ara*-5I** and ***ara*-5G** are characterized by low-lying singlet $\pi\pi_{ring}$* states and we expect that the corresponding photorelaxation channels on the $\pi\pi_{ring}$* hypersurface might play an important role in these nucleosides. The $^1\pi\pi_{ring}{}^*/S_0$ state crossing of **ara-5I** presented below, is characterized by peaked topography, which means that the S1 minimum coincides with this conical intersection geometry and the corresponding photodeactivation to the electronic groud-state occuring is an efficient and barrierless process. Despite this result, the TAS spectra showed sizeable population of the triplet states, which further confirms that intersystem crossing in 8-mercapto-*arabino*-purines is an ultrafast process, *i.e.* occuring on a sub-picosecond timescale. Our tentative calculations for ***ara*-5G** suggest that an analogous $^1\pi\pi_{ring}{}^*/S_0$ state crossing should be expected in this nucleoside.

In addition to the ESA spectra presented in the main article, we also simulated ESA spectra of the electronic states of which temporary population could be expected before performing the TAS measurements. Supplementary Figure 49 presents the ESA bands characteristic for the the $S_1(\pi\pi_{CS}{}^*)$ of ***ara*-5I**. Although we were able to confirm the initial population of the $S_1(\pi\pi_{CS}{}^*)$ state in ***ara*-5A**, we did not observe the characteristic spectral feature at 460 nm associated with the $S_1(\pi\pi_{CS}{}^*)$ state in ***ara*-5I** during the initial picoseconds of the TAS measurement. While such a feature is visible in the TAS spectrum of ***ara*-5I** after 3 ps of the measurement, we assigned it to the triplet $T_1(\pi\pi_{ring}{}^*)$ state due to its appeareance at longer time-delays and long persistence in the spectrum. This practically rules out that this singlet $\pi\pi_{CS}$* state is populated in ***ara*-5I**, and suggests that the dominant singlet excitation populated soon after the photoexcitation of this nucleoside is the $\pi\pi_{ring}$* state. This interpretation is consistent with the vertical excitation energies of ***ara*-5I** provided in Supplementary Table 1 which show that the $\pi\pi_{ring}$* state is the lowest lying singlet excitation available energetically near 315 nm. Our simulation of the ESA for the $S_1(\pi\pi_{ring}{}^*)$ state was hindered because the corresponding minimum energy geometry coincides with the S_1/S_0 conical intersection. However, our ESA simulations performed at this conical intersection geometry indicate that two distinctive absorption bands should be expected in a very similar spectral region to the characteristic ESA peak simulated for the triplet $T_1(\pi\pi_{CS}{}^*)$ state of ***ara*-5I**, e.g. 350 to 400 nm (see Supplementary Figure 50). Therefore, the TAS signal assigned to the $T_1(\pi\pi_{CS}{}^*)$ state in the main article, with the maximum at 350 nm, could in fact represent mixed

population of the $S_1(\pi\pi_{ring}*)$ and $T_1(\pi\pi_{CS}*)$ states. However, we emphasize that the theoretical ESA spectrum simulated at the S_1/S_0 conical intersection should be treated with caution due to the potentially multiconfigurational character of the wave-function in this region of the excited state potential energy surface and the fact that the system is not expected to linger for longer periods in the vicinity of this conical intersection.

According to our ADC(2)/cc-pVTZ geometry optimizations performed in the gas phase, both the ring-puckered and S out-of-plane minima on the T_1 hypersurface of ***ara*-5G** are nearly isoenergetic. This is schematically demonstrated in Supplementary Figure 51. The energy barrier separating these minima was obtained by the optimization of the T_2/T_1 minimum energy crossing point, which according to nonadiabatic transition state theory, corresponds to the saddle point (transition state) on the potential energy surface of the lower adiabatic state. This energy barrier amounts to ~0.53 eV and is sufficiently low to enable efficient interconversions between the two identified T_1 minima, after the lowest lying triple state is populated. Consequently, high availability of the S out-of-plane minimum should result in efficient sulfur loss and it should protect the nucleoside from being trapped in the long-lived $T_1(\pi\pi_{ring}*)$ ring-puckered minimum which was proposed as the origin of the destructive chemistry observed in ***ara*-5I**.

Supplementary References

1. Chattopadhyaya, J. B. & Reese, C. B. Interconversion of 8,2′-O-Cycloadenosine and 2′,3′-Anhydro-8-Oxyadenosine. *J. Chem. Soc. Chem. Commun.* 860–862 (1976).

2. Ikehara, M. & Ogiso, Y. Studies of Nucleosides and Nucleotides—LIV. Purine Cyclonucleosides - 19. Further Investigations on the Cleavage of the 8,2′-O-Anhydro Linkage. A New Synthesis of 9-β-D-Arabinofuranosyladenine. *Tetrahedron* **28,** 3695–3704 (1972).

3. Stairs, S. *et al.* Divergent Prebiotic Synthesis of Pyrimidine and 8-Oxo-Purine Ribonucleotides. *Nat. Commun.* **8,** 15270 (2017).

4. Powner, M. W. *et al.* On the Prebiotic Synthesis of Ribonucleotides: Photoanomerisation of Cytosine Nucleosides and Nucleotides Revisited. *ChemBioChem* **8,** 1170–1179 (2007).

5. Sanchez, R. A. & Orgel, L. E. Studies in Prebiotic Synthesis. V. Synthesis and Photoanomerization of Pyrimidine Nucleosides. *J. Mol. Biol.* **47,** 531–543 (1970).

6. Powner, M. W. Studies Towards a Chemical Origin of RNA. (PhD Thesis, The University of Manchester, 2009).

7. Ikehara, M. & Muraoka, M. Studies of Nucleosides and Nucleotides. LXVIII. Purine Cyclonucleosides. (29). Synthesis and Properties of O-Cyclonucleosides Derived From Hypoxanthine, Mercaptopurine, Methylmercaptopurine and Purine. *Chem. Pharm. Bull.* **24,** 672–682 (1976).

8. Chattopadhyaya, J. B. & Reese, C. B. Convenient Preparations of 9-β-D-Arabinofuranosyl guanine, 9-B-D-Arabinofuranosylhypoxanthine and Derivatives. *Synthesis (Stuttg).* **1978,** 908–910 (1978).

9. Reist, E. J., Calkins, D. F., Fisher, L. V. & Goodman, L. The Synthesis and Reactions of Some 8-Substituted Purine Nucleosides. *J. Org. Chem.* **33,** 1600–1603 (1968).

10. Holmes, E., Robins, K. & E, C. F. T. H. Purine Nucleosides. VII. Direct Bromination of Adenosine, Deoyadenosine, Guanosine and Related Purine Nucleosides. *J. Am. Chem. Soc.* **86,**

1242–1245 (1964).

11. Lin, T., Cheng, J., Ishiguro, K. & Sartorelli, A. C. 8-Substituted Guanosine and 2′-Deoxyguanosine Derivatives as Potential Inducers of the Differentiation of Friend Erythroleukemia Cells. *J. Med. Chem.* **28,** 1194–1198 (1985).

12. Lin, T. S., Cheng, J. C., Ishiguro, K. & Sartorelli, A. C. Purine and 8-substituted Purine Arabinofuranosyl and Ribofuranosyl Nucleoside Derivatives as Potential Inducers of the Differentiation of the Friend Erythroleukemia. *J. Med. Chem.* **28,** 1481–1485 (1985).

13. Long, R. A., Robins, R. K. & Townsend, L. B. *In Synthetic Procedures in Nucleic Acid Chemistry; Zorbach, W. W., Tipson, R. S., Eds.; Wiley-Interscience: New York, NY, Vol. 1,* (1968).

14. Stairs, S. & Powner, M. Scalable Synthesis of 2,2′-Anhydro-arabinofuranosyl Imidazoles. *Synlett* **28,** 2650–2654 (2017).
